# Preparative and Catalytic Properties of Mo^VI^ Mononuclear and Metallosupramolecular Coordination Assemblies Bearing Hydrazonato Ligands

**DOI:** 10.3390/ijms25031503

**Published:** 2024-01-25

**Authors:** Mirna Mandarić, Edi Topić, Dominique Agustin, Jana Pisk, Višnja Vrdoljak

**Affiliations:** 1 Department of Chemistry, Faculty of Science, University of Zagreb, Horvatovac 102a, 10000 Zagreb, Croatia; mirna.mandaric@chem.pmf.hr (M.M.); edi.topic@chem.pmf.hr (E.T.); jana.pisk@chem.pmf.hr (J.P.); 2 IUT P. Sabatier, Department of Chemistry, University of Toulouse, Av. G. Pompidou, BP20258, 81104 Castres CEDEX, France; dominique.agustin@iut-tlse3.fr; 3 CNRS (Centre National de la Recherche Scientifique), LCC (Laboratoire de Chimie de Coordination), 205 Route de Narbonne, BP44099, 31077 Toulouse CEDEX 4F, France

**Keywords:** molybdenum, hydrazone, metallosupramolecular, dinuclear, metallacycles, polymers, epoxidation, cyclooctene, catalysis, DFT

## Abstract

A series of polynuclear, dinuclear, and mononuclear Mo(VI) complexes were synthesized with the hydrazonato ligands derived from 5-methoxysalicylaldehyde and the corresponding hydrazides (isonicotinic hydrazide (H_2_L^1^), nicotinic hydrazide (H_2_L^2^), 2-aminobenzhydrazide (H_2_L^3^), or 4-aminobenzhydrazide (H_2_L^4^)). The metallosupramolecular compounds obtained from non-coordinating solvents, [MoO_2_(L^1,2^)]*_n_* (**1** and **2**) and [MoO_2_(L^3,4^)]_2_ (**3** and **4**), formed infinite structures and metallacycles, respectively. By blocking two coordination sites with cis-dioxo ligands, the molybdenum centers have three coordination sites occupied by the *ONO* donor atoms from the rigid hydrazone ligands and one by the N atom of pyridyl or amine-functionalized ligand subcomponents from the neighboring Mo building units. The reaction in methanol afforded the mononuclear analogs [MoO_2_(L^1-4^)(MeOH)] (**1a**–**4a**) with additional monodentate MeOH ligands. All isolated complexes were tested as catalysts for cyclooctene epoxidation using *tert*-butyl hydroperoxide (TBHP) as an oxidant in water. The impact of the structure and ligand lability on the catalytic efficiency in homogeneous cyclooctene epoxidation was elucidated based on theoretical considerations. Thus, dinuclear assemblies exhibited better catalytic activity than mononuclear or polynuclear complexes.

## 1. Introduction

Metallosupramolecular compounds are species formed from metal ions and organic ligands in coordination-driven self-assembly processes [1,2,3,4,5]. Aesthetically fascinating architectures can be self-assembled from the same building blocks depending on metal coordination geometry, ligand structure, metal-to-ligand ratio, solvent, concentration, and presence of guests. The formation of zero-dimensional, polyhedral, or infinite structures reflects a complex interplay between kinetic and thermodynamic contributions [6,7,8,9]. 

The interest in this class of compounds comes not only from the challenges related to the development of reliable synthetic strategies and rational design but also from their wide-ranging potential. Thus, those assemblies can find application in catalysis [10,11], optics [12,13,14], magnetism [15,16,17], gas storage separation [18,19], and as sensors [20,21]. Considering the importance of integrating green chemistry principles and sustainability into research, the development of novel catalysts is essential, particularly those that can exhibit high activity and selectivity at mild operating conditions, which is of high interest.

The catalytic properties of molybdenum coordination compounds [22,23,24,25] have led to an increased interest in their metallosupramolecular chemistry in years. As a result, the investigation has grown from essential design principles enabling the formation of the first coordination polymers and metallocycles [26,27,28,29] to exploration of their structural and compositional fine-tuning in order to obtain catalysts with excellent properties [30,31,32,33,34,35]. Accordingly, constrained cyclic Mo(VI) metallosupramolecular complexes were recently found to be among the most effective catalysts for olefin epoxidation [20,21]. 

There is still a promising pool of catalysts based on metallosupramolecular or simple mononuclear (dioxo)Mo(VI) coordination assemblies. Such materials can utilize the advantages of organic components for fine structural tuning and, consequently, their properties. In particular, the compounds with hemilabile coordination bonds are of interest as they could serve as high-efficiency catalysts. For example, the high performance of hexacoordinated Mo(VI) (pre)catalysts closely correlates with their possibility of converting into the pentacoordinated counterparts [34,36].

**Scheme 1:**
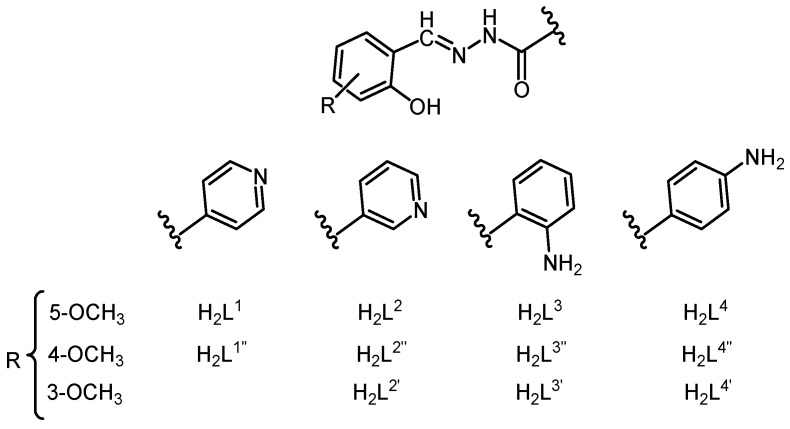
Hydrazones derived from 5-methoxysalicylaldehyde and the corresponding hydrazides: isonicotinic hydrazide (H_2_L^1^), nicotinic hydrazide (H_2_L^2^), 2-aminobenzhydrazide (H_2_L^3^), or 4-aminobenzhydrazide (H_2_L^4^). Previously published ligands derived from 3- or 4-methoxysalicylaldehyde are denoted as H_2_L′ and H_2_L′′, respectively.

To explore the (dioxo)Mo(VI) complexes and their catalytic properties, four hydrazone ligands have been selected (Scheme 1) where the subunit derived from 5-methoxysalicylaldehyde remained unchanged. It was expected that the catalytic activity could be modulated by the hydrazide moiety. Self-assembly of {MoO_2_}^2+^ units with ligands bearing (iso)nicotinoyl or aminobenzoyl moieties in dichloromethane yielded metallosupramolecular polymers and dimers, respectively, based on metal–nitrogen linkages. The reaction in methanol gave rise to mononuclear coordination compounds containing methanol (MeOH) ancillary ligands with metal–oxygen bonds. Crystal and molecular structures of the two hydrazones, one dinuclear, and three mononuclear coordination compounds were determined using the single-crystal X-ray diffraction method. All compounds were additionally characterized using elemental analysis, powered X-ray diffraction, thermogravimetric, and spectroscopic methods.

The molybdenum-catalyzed epoxidation of alkenes is an important process as it allows the conversion of simple hydrocarbon substrates into high-value fine chemicals. We, therefore, explored the effects of Mo–N (N being a pyridyl or amine nitrogen atom) or Mo–O (O being a MeOH oxygen atom) bond liability on the catalytic performances of materials obtained in the oxidation of cyclooctene. As in our previous works, we focused on catalytic reactions under environmentally friendly conditions without organic solvents. The catalytic efficiency of the Mo-coordination entities was evaluated using *tert*-butyl hydroperoxide as the oxidant in water. Catalytic results are discussed in relation to the complex nuclearity, coordination environment, and ligand structure. The obtained results were compared to the previously published results utilizing corresponding H_2_L′ and H_2_L′′ ligands, respectively (Scheme 1).

## 2. Results and Discussion

### 2.1. Synthesis and Structural Characteriation

#### 2.1.1. Hydrazones

Hydrazones H_2_L^1^–H_2_L^4^ (Scheme 1) were afforded by reacting equimolar amounts of 5-methoxysalicylaldehyde with the corresponding hydrazides (isonicotinic hydrazide, nicotinic hydrazide, 2-aminobenzhydrazide, or 4-aminobenzhydrazide) in a methanolic solution. In the case of H_2_L^1^ and H_2_L^2^, the reagents were heated under reflux. The synthesis of H_2_L^3^ and H_2_L^4^ required lowering the reaction temperature primarily to avoid the secondary condensation reaction of the aldehyde with the amine functional group. All compounds, except H_2_L^4^, remained stable in the solid state for a longer time at room temperature. However, upon exposure to the air, hydrazone H_2_L^4^ transformed over time to the more stable hydrate H_2_L^4^·H_2_O. 

**Figure 1:**
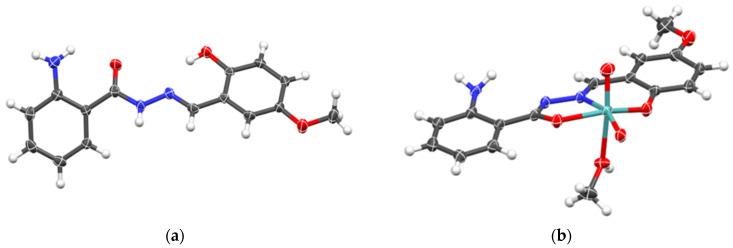

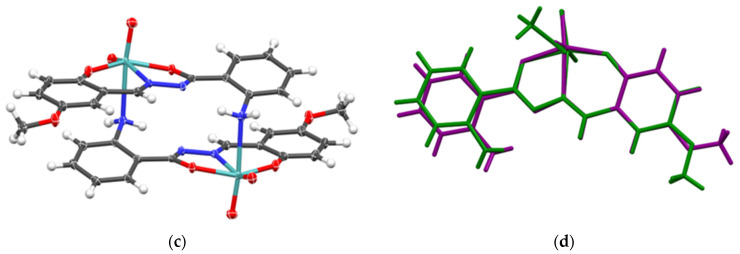
Molecular structure of (**a**) H_2_L^3^, (**b**) **3a**, (**c**) **3**, with atoms represented as thermal ellipsoids at 50% probability level. Structural overlay of mononuclear complex **3a** (green) and complex dimer **3** (purple) is shown in (**d**), showing the similarity of ligand conformation in both cases.

The crystal structures of H_2_L^1^ and H_2_L^1^·H_2_O were reported previously [37,38]. Ligands H_2_L^3^ and H_2_L^4^ crystallize in orthorhombic *Pna*21 and triclinic *P*-1 space groups, respectively (Figure 1a, Supplementary Materials, Figure S1, Table S1). The crystal structure of H_2_L^3^ contains one molecule of ligand in the asymmetric unit, which packs in an alternating zig-zag fashion (Supplementary Materials, Figure S2), as dictated by a singular intermolecular hydrogen bond between hydrazide N–H donor and aryl hydroxy group acceptor (Supplementary Materials, Figure S3a). Meanwhile, in the crystal structure of H_2_L^4^, one can find two symmetrically inequivalent ligand molecules, which form a complex hydrogen-bonded network via interactions between hydrazide and aryl amine donors, and amide keto and aryl hydroxy group acceptors (Supplementary Materials, Figure S3b). However, both ligands crystallize in keto-amino tautomeric form (Supplementary Materials, Scheme S1), as evidenced by C2–O2, C2–N2, and N2–N1 bond lengths (Supplementary Materials, Table S2), which correspond to lengths expected for double, single and double bond, respectively. Additionally, both ligands are in *E* conformation (Supplementary Materials, Table S3). Hydrogen bond parameters in the crystal structure of H_2_L^3^ and H_2_L^4^ are given in Table S4 (Supplementary Materials).

#### 2.1.2. Metallosupramolecular Coordination Assemblies

The Mo(VI) metallosupramolecular complexes **1**–**4** were synthesized via the reaction of [MoO_2_(acac)_2_] (acac = acetylacetonate) with H_2_L^1^–H_2_L^4^, respectively, in CH_2_Cl_2_ at room temperature. The formulae of [MoO_2_(L^1,2^)]*_n_* (**1** and **2**) and [MoO_2_(L^3,4^)]_2_ (**3** and **4**), containing the corresponding ligand in a doubly deprotonated form, are consistent with elemental, thermogravimetric analysis, spectroscopic, and X-ray diffraction data. Their formation is in accordance with the energy-related principle of “maximum site occupancy”, according to which species with all the coordination sites occupied are more stable than those with vacant sites [39]. 

Synthesis with ligands H_2_L^1^ or H_2_L^2^ bearing nitrogen-containing heterocyclic ring resulted in the coordination polymers, comprising Mo coordinated by one hydrazone ligand through *ONO* donor atoms (phenolic-oxygen, azomethine-nitrogen, and hydrazidic-oxygen) and the second ligand through the isonicotinoyl *N* atom from the adjacent Mo complex unit. Although polymer **1** has been previously reported (CSD refcode ZILVIB) [40], it was prepared under different conditions. The polymer forms infinite chains, the topology of which is dictated by the rigid geometry of the isonicotinoyl fragment and {MoO_2_}^2+^ core. The only degree of freedom in this chain is the Mo–N_pyridyl_ bond length, which is slightly longer than expected for similar complexes with *ONO* ligand coordinated on the {MoO_2_}^2+^ core and pyridyl fragment coordinated on the free axial site. Particularly, the bond length in **1** is found to be 2.455 Å as opposed to an average of 2.378 ± 0.082 Å for 102 structures in the CSD database [41]. This indicates significant steric impedance in the assembly of **1** polymer.

Ligands modified by incorporating the amino functionality, H_2_L^3^ and H_2_L^4^, gave rise to the cyclic dimers [MoO_2_(L)^3,4^]_2_ (**3** and **4**). The molecular structure of **3** is shown in Figure 1c. Selected geometric data are given in Supplementary Materials, Table S2. The formation of the dimeric compound [MoO_2_(L^4^)]_2_ (**4**) was proposed based on literature data [34]. Additionally, the favored formation of dimers over polymers is related to the preferred formation of smaller molecules containing fewer building blocks [42].

The structure of **3** (Supplementary Materials, Figures S1, S2 and S4) exhibits a distorted octahedral geometry around the Mo(VI) metal center (Supplementary Materials, Tables S6 and S7). The *ONO* ligand chelates in a meridional fashion occupying three coordination sites. Two *cis*-oriented oxido ligands and the amino *N* atom from the adjacent Mo complex unit complete the coordination sphere. Similar to **1**, one can analyze the oligomerization “propensity” based on the observed Mo–N_amine_ length. In **3**, that length is 2.536(2) Å, whereas for the 11 known examples in the CSD database, the mean length is 2.534 ± 0.098 Å. One must observe that complexes derived from H_2_L^3^ and H_2_L^4^ have greater conformational flexibility when considering the coordination of the amine nitrogen atom, compared to the coordination of the pyridyl nitrogen atom in **1** and **2**. The PXRD patterns of **1** and **3** were in good agreement with the simulated patterns of the corresponding crystal structures (Supplementary Materials, Figure S5).

#### 2.1.3. Mononuclear Coordination Assemblies

In the reaction between the [MoO_2_(acac)_2_] complex and hydrazone ligands in methanol, the coordination of the pyridyl/amine moiety was not observed. Instead, the [MoO_2_(L^1–4^)(MeOH)] (**1a**–**4a**) compounds crystallized the additional monodentate MeOH ligand (Figure 1b, Supplementary Materials, Figure S1). Complexes **1a**–**4a** were obtained in higher yield (64–89%) than the metallosupramolecular species **1**–**4** (31–38%). A single-crystal X-ray study of **1a**, **2a**, and **3a** confirmed the structures to be mononuclear. Experimental powder diffractograms of all obtained samples matched well with the calculated ones (Supplementary Materials, Figure S6). Complexes **1a**–**4a** were stable in the solid state at room temperature. However, their quantitative conversion to polynuclear **1** and **2** or dinuclear forms **3** and **4** was observed upon heating in acetone or acetonitrile. 

The crystal structures of **1a**, **2a**, and **3a** (Supplementary Materials, Figures S1 and S2) show features already well established for the majority of [MoO_2_(L)(MeOH)] complexes. The {MoO_2_}^2+^ core has the same coordinative environment as in polymers (see Section 2.1.2), except for the axial coordination site occupied with oxygen atoms from methanol molecule. Sterically undemanding methanol ligand has a well-defined coordinative bond length of 2.343 ± 0.035 Å based on 206 examples in the CSD database, and complexes **1a**, **2a** and **3a** have this length in the same ballpark, albeit a bit shorter (2.313(2), 2.295(7), and 2.287(4) Å, respectively). Selected bond lengths and angles are given in the Supplementary Materials, Tables S6 and S7.

Crystal packing (Supplementary Materials, Figure S2) and supramolecular interactions in prepared methanol complexes are dictated by a small number of hydrogen bond donors (namely, methanol OH and amine NH_2_ moieties), forming relatively simple supramolecular chains. However, due to the presence of an amino group donor, supramolecular chain in **3a** is built upon supramolecular dimers, akin to coordinative dimers of **3**, whereas supramolecular chains in **1a** and **2a** are made of monomers interacting solely through methanol OH∙∙∙N_pyridyl_ hydrogen bond (Supplementary Materials, Figure S4, Table S8).

### 2.2. Spectroscopic Characterization

The infrared spectra confirmed the presence of the amide tautomer, Supplementary Materials, Figure S7. The intense absorptions in the range of 1695-1653 cm^−1^ and 1654–1626 cm^−1^ are attributed to the C=O and C=N imine bond vibrations, respectively. Hydrazones H_2_L^1^ and H_2_L^2^ exhibited bands at approximately 3260 cm^−1^ and 3220 cm^−1^ assigned to –O–H and =N–NH, whereas these bands appeared at approximately 3500 cm^−1^ and 3400 cm^–1^ for H_2_L^3^ and H_2_L^4^. The medium intensity band observed at 1163 cm^−1^–1115 cm^−1^ was assigned to N–N stretching vibrations [43].

The stretch bands of C=O, –O–H, and =N–NH that were characteristic of free hydrazones disappeared upon metal complexation. The presence of bands in **1**–**4** belonging to C–O_hydrazone_ (at 1320–1345 cm^–1^), C–O_phenolic_ (at 1260–1275 cm^–1^), and C=N (at 1600–1610 cm^–1^) indicated hydrazone functionality tautomerization N–NH–C=O → N–N=C–OH, deprotonation, and the molybdenum atom coordination through hydrazone *ONO* donor atoms. Strong absorption bands were characteristic of the {MoO_2_}^2+^ core in the range of 935–908 cm^–1^ and weaker intensity bands for Mo–N bonds below 900 cm^–1^ corroborated the metallosupramolecular complex formation [34]. The methanol coordination in **1a**–**4a** was supported by a new absorption band typical of C–O_MeOH_ at around 1030 cm^−1^. Additionally, the mononuclear complexes exhibited stretching frequencies O=Mo–O_MeOH_ around 900 cm^−1^. The –NH_2_ bond vibrations of the mononuclear complexes present as two medium-intensity bands (3356–3263 cm^–1^) are higher in energy than those of the dinuclear complexes (3334–3227 cm^–1^), Supplementary Materials, Figures S8 and S9. A similar position of these bands was found in the spectra of the previously published complexes [MoO_2_(L^4^′)]_2_ and [MoO_2_(L^4^′′)]_2_ [34].

The proton and carbon nuclear magnetic resonance (NMR) chemical shifts of H_2_L^1^–H_2_L^4^ (Supplementary Materials, Tables S9–S12, Schemes S2–S5, Figures S10–S13) were assigned by ^1^H, ^13^C{^1^H} attached proton test (APT), ^1^H–^13^C heteronuclear multiple quantum coherence (HMQC), and heteronuclear multiple bond correlation (HMBC) experiments in dimethyl sulfoxide-*d*_6_ (DMSO-*d*_6_). Downfield shifts in the range of 11.02–10.66 ppm and 12.37-11.88 ppm suggested the presence of –OH and =N–NH groups, respectively, thus indicating the amide form =N–NH–(C=O)– in solution. Singlets at 6.54 and 5.89 confirmed the presence of –NH_2_ protons in the spectra of H_2_L^3^ and H_2_L^4^, respectively [44]. The OH, N=NH, and NH_2_ signals were broadened to some extent due to their involvement in hydrogen bonding. Singlets at 8.75-8.63 ppm and 3.77–3.73 ppm were assigned to the CH=N and OCH_3_ groups, respectively. The aromatic moieties gave signals in the range of 9.19–6.65 ppm [45]. In the ^13^C NMR spectra of H_2_L^1^-H_2_L^4^, the py and phenyl hydrazone moiety signals C5–C10 were observed in the range of 151.90–121.06 ppm and 152.03–112.09 ppm, respectively. The signals in the range of 164.59–160.94 ppm and 148.07–145.95 ppm were attributed to C4 of the C=O group and C1 carbon of the CH=N group, respectively.

Due to the donor properties of the solvent DMSO-*d*_6_, the metallosupramolecular nature of complexes **1**–**4** and the presence of ancillary ligand in the mononuclear complexes **1a**–**4a** were disrupted, and complexes [(MoO_2_)_2_(L^1–4^)(DMSO)] appeared instead. The signals for the (iso)nicotinoyl and aminobenzoyl protons did not exhibit any appreciable change in the chemical shift. In the ^1^H NMR spectra, the absence of –OH and =N–NH protons in the low field region indicated their deprotonation upon complexation. Multiple groups of signals in the range of 6.57–9.15 ppm were due to aromatic ring protons. The most significant coordination-induced difference between signals was noticed for imine CH=N, which was up to 0.3 ppm. In the ^13^C NMR spectra, the complexation-induced differences in the chemical shifts were the most pronounced for C1 and C4 carbons, which experience deshielding effects up to 9.28 ppm and 6.74 ppm, respectively. The chemical shifts for phenolic C12 carbons differed less noticeably, which was up to 2.6 ppm. 

### 2.3. Thermal Analysis 

The first mass loss in the thermogravimetric (TG) curves of **H_2_L^4^∙H_2_O** is related to the removal of a water molecule from a crystalline hydrate (starting at 105 °C). The data from the differential scanning calorimetry (DSC) measurement shows a broad endothermic peak due to the evaporation of the water during the dehydration process. From DSC measurements, the melting onset point temperature for anhydrous hydrazone **H_2_L^1^** is at 197 °C, **H_2_L^2^** at 111 °C, **H_2_L^3^** at 159 °C, and **H_2_L^4^** at 191 °C with a 10 °C/min scanning rate. They start to decompose at 270 °C, 283 °C, 211 °C, and 289 °C, respectively.

The thermal behavior of the complexes was studied in the temperature region from 25 to 600 °C in an oxygen atmosphere. The polynuclear and dinuclear complexes **1**–**4** demonstrated thermal stability of up to 300 °C. Afterwards, the decomposition of complexes occurred in one step. The TG curves of **1a**–**4a** showed two distinct mass losses: the first one due to MeOH removal at temperatures around 140 °C, and the second one due to decomposition (with onset temperatures of 333 °C, 317 °C, 286 °C, and 281 °C, respectively). 

### 2.4. Cyclooctene Epoxidation with TBHP 

**Figure 2:**
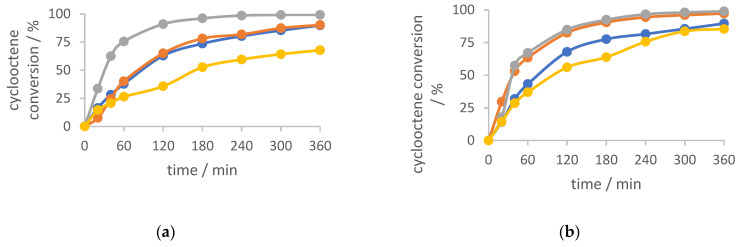
Kinetic profiles of cyclooctene conversion with catalysts (**a**) **1**–**4** and (**b**) **1a**–**4a**. The blue curve presents the reaction with catalysts **1** (left) and **1a** (right), the orange curve presents the reaction with catalysts **2** (left) and **2a** (right), the grey curve presents the reaction with catalysts **3** (left) and **3a** (right), and the yellow curve presents the reaction with catalysts **4** (left) and **4a** (right). TBHP in water was used as an oxidizing agent.

Cyclooctene epoxidation reactions were performed with *tert*-butylhydroperoxide (TBHP) in water as an oxidizing agent. The idea was to test the prepared molybdenum complexes as catalysts, following the protocol previously established, to compare the activity and selectivity parameters with the results already published. For that reason, the green chemistry protocol was applied. Even though TBHP is commercially available in decane and water, and the catalytic results with the assistance of TBHP in decane are often much better, the choice of TBHP in water justifies the green catalytic concept. All the complexes were tested as catalysts with 0.25 % catalyst loading. The obtained results are shown in Table 1 and the catalytic profiles are presented in Figure 2.

Due to the distinct organic and aqueous phases in the catalytic reaction, the analysis focused solely on the organic phase using gas chromatography (GC). The presence of epoxide in the organic phase was identified, with the selectivity parameter ranging from 80 to 98%. Consistent with previous reports, the GC analysis did not extend to the aqueous layer. Drawing from our earlier studies, it has been established that the epoxide may undergo opening to form the corresponding diol, contingent on the presence of the diol in the water phase.

All the tested catalysts showed very good activity, with cyclooctene conversion being more than 85 %, with the exception of the dinuclear catalyst **4** (67 %). TOF_20 min_ parameter is the highest for dinuclear catalyst **3**, meaning that it converts to the catalytically active species in the shortest time, and it is the lowest for catalyst **2**. It can also be noticed that the catalytic profiles of catalysts **1a** and **2a** had a very similar pattern, directly implying that the position of the N atom on the hydrazide part of the ligand (third or fourth atom) had almost no effect on the catalytic behavior. Furthermore, a similar trend was also noticed for catalysts **3a** and **4a**, indicating that the position of the amino group (on the second or fourth C atom) did not influence the final activity outcome. What is quite interesting is the comparison of the TOF parameter. It is obvious that the dinuclear compounds **3** and **4**, with the TOF_20 min_ values of 415 and 279, respectively, are more easily converted to the catalytically active species than the polynuclear compounds **1** and **2**. On the contrary, the mononuclear complexes **1a** and **2a** have very similar TOF_20 min_ values (around 350), almost twice higher than the TOF_20 min_ values for **3a** and **4a**.

All the obtained results can be compared to the similar published results compiled in Table 2. The hydrazone derived from 3- or 4-methoxysalicylaldehyde with the corresponding hydrazides (nicotinic hydrazide, 2-aminobenzhydrazide or 4-aminobenzhydrazide) used for the coordination to the molybdenum center are presented in Scheme 1.

As noted, complex **2** can be compared to the complexes [MoO_2_(L^2′^)]*_n_* and [MoO_2_(L^2′′^)]*_n_*, showing the activity trend **2** > [MoO_2_(L^2′′^)]*_n_* > [MoO_2_(L^2′^)]*_n_*. Furthermore, the selectivity parameter followed the same trend, meaning that the position of the –OMe group on the aldehyde part of the ligands has a strong impact on the catalytic performances, favoring the fifth position on the benzene ring.

On the other side, the influence of the –OMe group on the aldehyde part of the ligands was not so pronounced when comparing catalysts **3** and **3a** with [MoO_2_(L^3′^)]_2_, [MoO_2_(L^3′′^)(MeOH)] and [MoO_2_(L^3′′^)]_2_. For the TOF and activation energy, there is a bigger difference between **3** (the highest TOF) and **3a** (the lowest one) compared to the three other complexes with relatively similar TOF for both dimers and slightly higher for the methanol-coordinated species. This behavior seems to indicate that the possibility of the decoordination and formation of the pentacoordinated active species has a strong impact on the mechanisms.

However, the –OMe position impacts the activity of the complexes **4** and **4a** when correlated with [MoO_2_(L^4′^)(MeOH)], [MoO_2_(L^4′′^)(MeOH)], [MoO_2_(L^4′^)]_2_, and [MoO_2_(L^4′′^)]_2_. The methanol coordinated compounds, [MoO_2_(L^4′^)(MeOH)] and [MoO_2_(L^4′′^)(MeOH)], have lower conversion and TOF than catalyst **4a**, while the cyclooctene conversion for dinuclear catalysts has an unusual trend [MoO_2_(L^4′′^)]_2_ > [MoO_2_(L^4′^)]_2_ > **4** but with very close TOF values. In this case, we can conclude that there is an effect of methanol that has to be investigated in order to explain the mechanism. However, in general, it can be concluded that the catalysts bearing –OMe group on the fifth C atom of the benzene ring show better catalytic performances, except in the case when NH_2_ is in the fourth position on its aromatic ring, complex **4**.

### 2.5. Density Functional Theory (DFT) Calculations

It is interesting to see if the experimental results can be validated by theoretical calculations to understand the mechanisms and the effect of the ligand on the catalytic activity. The main points responsible for such activity and the correlation of the catalytic activity with the structure are possible to be answered when the ligand functionalization is very different. However, subtle changes, as in the presented research, are sometimes difficult to analyze using DFT calculations.

The mechanism proposed several years ago [36] postulated that [MoO_2_(L)(D)]or [MoO_2_(L)]*_n_* species turned into a pentacoordinated species [MoO_2_(L)] before reacting with TBHP to create the transient species [MoO_2_(L)(TBHP)], the latter permitting an easier epoxide formation through the O_β_ of the epoxide. It has been shown that the presence of substituents with strong electronic effects on the ligands had dramatic effects on the catalysis [36]. These results were linked to different electronic effects that could even be assessed using the geometrical features of the calculated species. 

In the present case, the differences between the four ligands lie in the hydrazide nature: N atom is within the aromatic ring (in the case of complexes with L^1–^ and L^2–^ ligands) or bonded to the aromatic ring as NH_2_ (in the case of complexes with L^3–^ and L^4^ ligands). In the case of coordination polymers **1** and **2**, an insight into decoordination through DFT would be too complicated. Therefore, we limited the discussion about the decoordination to complexes **3** and **4**. The crystal structure of compound **3** was determined as a dimer using the X-ray diffraction method, and therefore, the calculations for compound **4** were adopted according to the known structure. Further calculations included mononuclear complexes **1a**-**4a**. 

**Scheme 2:**
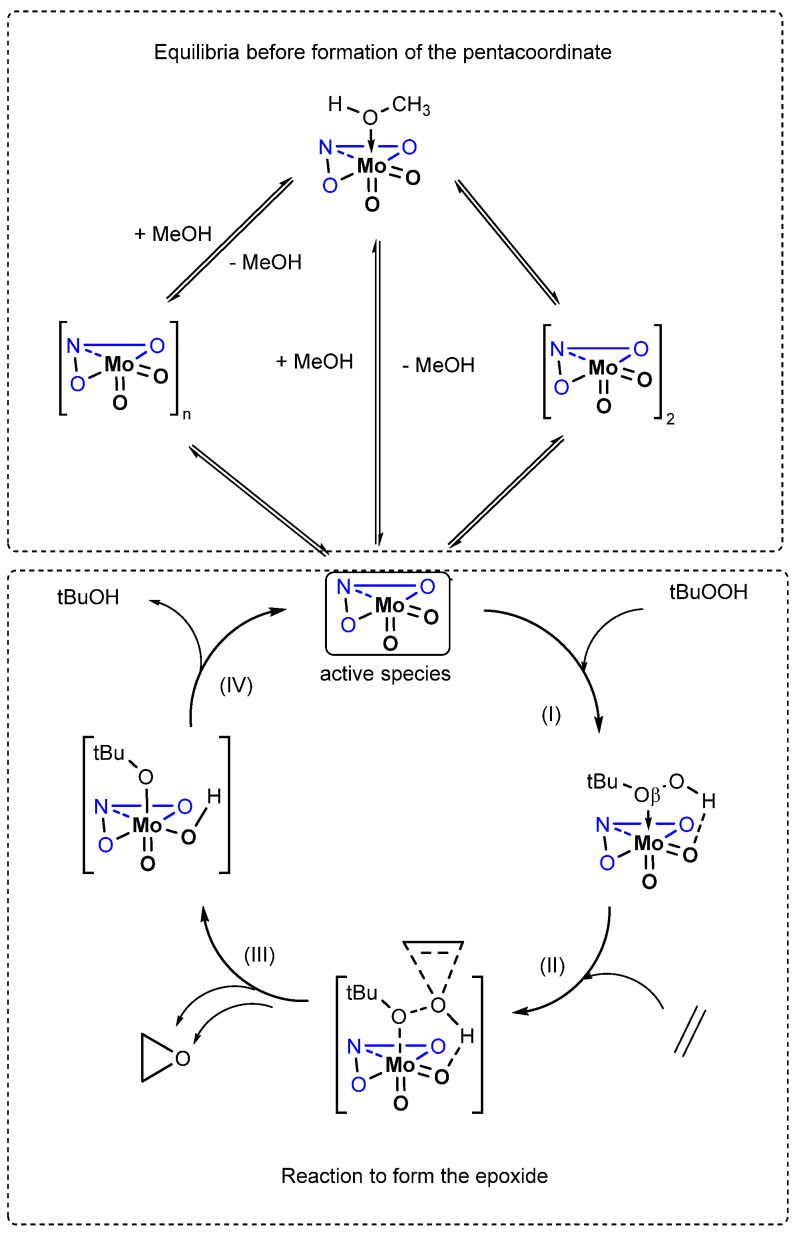
Presentation of the postulated mechanism for the epoxidation olefin. The *ONO* atoms (blue) and lines symbolize the ligands. The different steps (I–IV) are indicated by numbers close to the arrows.

We limited the calculations to the gas phase since trends can be observed from these conditions [47], and the data are shown in Table 3. All other calculation steps are shown in Scheme 2. The optimized geometries are listed in Supplementary Materials, Table S14.

Experimentally, MeOH coordination does not lower the activity in the case of [MoO_2_(L^1^)(MeOH)] (**1a**) and [MoO_2_(L^2^)(MeOH)] (**2a**), but lowers it in the case of [MoO_2_(L^3^)(MeOH)] (**3a**) and [MoO_2_(L^4^)(MeOH)] (**4a**). It has to be considered that, once the methanol decoordinated, the resulting pentacoordinated complex [MoO_2_(L)] can have several options: (i) turning back into the mononuclear complex [MoO_2_(L)(D)], (ii) oligomerize or (iii) interact with TBHP. From the absolute enthalpy values obtained by calculations for each step (Table 3), the decoordination of the methanol (step 0) lies in the range of 10.3 to 11.1 kcal and these values do not provide a clear conclusion. In an identical manner, in the case of the stabilization of the formed pentacoordinate species [MoO_2_(L)] with TBHP on the Mo center (step I), the values are also very close for all four ligands. On the other side, TS enthalpies (step II) were ligand-dependent and the trend observed was TS^2^ < TS^1^ < TS^3^ < TS^4^. The lower the TS, the more easily the active species are formed, and consequently, TOF_20 min_ should be higher. This is in relative accordance with the experimentally obtained TOF values for the complexes **1a**–**4a**, TOF^2a^ ≈ TOF^1a^ > TOF^3a^ >TOF^4a^. 

In contrast, for the dinuclear complexes **3** and **4**, the order of the experimental TOFs, TOF^4^ < TOF^3^ cannot follow the calculated TS values, TS^3^ > TS^4^. It is experimentally expected that dimers **3** and **4** are transformed more easily than polymers **1** and **2**. Even if it is not simple to draw conclusions between dimers and polymers, a comparison between dimers and methanol-coordinated monomers **3** and **3a** (and **4** and **4a**) can be performed. At this point, the experimental TOF values can be linked to the TS shift considering the enthalpy of the starting molecule. The enthalpy to maintain those dimers is smaller than that for the methanol-coordinated ones, and this difference provokes a relative TS around 5 kcal lower for the polymers. This seems to be relevant enough to give some ideas about the TOF difference in this case.

It is interesting to see whether these activities can be deduced from the calculated geometric data. The relevant distances and dihedral angles in absolute values (related to ligand planarity) around the molybdenum atom are listed in Table 4. The experimental results showed that the [MoO_2_(L^3^)]_2_ (**3**) species seemed to be the best catalyst. Thus, as shown in the energy diagram, the Mo–O_β_ interatomic distance parameter mainly influenced the TS path (step II). As explained previously, a lower TS value (higher activity) is linked to a shorter distance. This is the case if we compare the analog complexes with L^3^ and L^4^ ligands. Interesting facts appeared with the N1 bonded to Mo, such as the shorter Mo-N^1^ distances in the TS were for the least active compounds.

Ligand distortion seemed to have an influence. While the [MoO_2_(L)(MeOH)] dihedral angle lies between 9.5 and 11.3°, the removal of the MeOH molecule favors the planarity (5.6 to 6.3) addition of TBHP increases the dihedral angle and, in TS, distortion is the highest.

## 3. Materials and Methods

### 3.1. Preparative Part

Elemental analyses were provided by the Analytical Services Laboratory of the Ruđer Bošković Institute, Zagreb. NMR data are given in Tables S9–S12 and Figures S5–S8 in the Supplementary Materials. All reagents and solvents were commercially available (Alpha-Aesar (Karlsruhe, Germany), Sigma-Aldrich (Chemie GmbH, München, Germany)) and were used without further purification. [MoO_2_(acac)_2_] was prepared according to the published procedure [48].

### 3.2. Synthesis of H_2_L^1^ and H_2_L^2^

A mixture of 5-methoxysalicylaldehyde (0.76 g, 5 mmol) and isonicotinic or nicotinic hydrazide (0.69, 5 mmol) in 100 mL of methanol was heated at reflux temperature with continuous stirring for 3 h. The resulting solution was concentrated under reduced pressure to one-quarter of its volume and left at room temperature for several days. The obtained product H_2_L^1^ or H_2_L^2^ was filtered and dried to a constant weight. Yield: 1.05 g, 77% (H_2_L^1^); 1.14 g, 84% (H_2_L^2^).

### 3.3. Synthesis of H_2_L^3^ and H_2_L^4^

5-methoxysalicylaldehyde (0.76 g, 5 mmol) dissolved in 40 mL of methanol was added dropwise to a solution of 2- or 4-aminobenzhydrazide (0.75 g, 5 mmol in 75 mL of methanol), stirred at room temperature for 30 min, and then heated at 40 °C for 4 h. The resulting solution was concentrated to 10 mL in vacuo and left at room temperature for several days. The obtained product was filtered and dried in a desiccator to a constant weight. Yield: 0.99 mg, 70% (H_2_L^3^); 1.15 mg, 81% (H_2_L^4^). 

### 3.4. Synthesis of ***1***–***4***

[MoO_2_(acac)_2_] (0.05 g, 0.15 mmol) was dissolved in dichloromethane (50 mL) and H_2_L^1-4^ (0.042 g H_2_L^1,2^ or 0.087g H_2_L^3,4^; 0.15 mmol) was added. Each suspension was shaken (at 50 rpm) for 6 hours at room temperature and left overnight. The resulting red product was collected via filtration and washed with a small amount of dichloromethane.

[MoO_2_(L^1^)]*_n_* (**1**): Yield: 42 mg, 34%. Anal. Calcd. for C_14_H_11_MoN_3_O_5_ (397.194): C, 42.33; H, 2.79; N, 10.58. Found: C, 42.19; H, 2.57; N, 10.39%. TG: MoO_3_, 35.94% (Calcd. 36.05%). Selected IR data (cm^−1^): 1600 (C=N), 1344 (C–O_hydrazone_), 1270 (C–O_phenolate_), 935, 917 (MoO_2_), and 904 (Mo–N).

[MoO_2_(L^2^)]*_n_* (**2**): Yield: 38 mg, 31%. Anal. Calcd. for C_14_H_11_MoN_3_O_5_ (397.194): C, 42.33; H, 2.79; N, 10.58. Found: C, 42.14; H, 2.55; N, 10.36%. TG: MoO_3_, 35.94% (Calcd. 36.02%). Selected IR data (cm^−1^): 1615, 1601 (C=N), 1334 (C–O_hydrazone_), 1265 (C–O_phenolate_), 934, 915 (MoO_2_), and 901 (Mo–N).

[MoO_2_(L^3^)]_2_ (**3**): Yield: 48 mg, 38%. Anal. Calcd. for C_30_H_26_Mo_2_N_6_O_10_ (822.440): C, 43.81; H, 3.19; N, 10.22. Found: C, 43.65; H, 3.02; N, 10.03%. TG: calcd. for MoO_3_, 35.00%, found 35.18%. Selected IR data (cm^−1^): 3285, 3227 (NH_2_), 1610, 1598 (C=N), 1332 (C–O_hydrazone_), 1270 (C–O_phenolate_), 925, 917 (MoO_2_^2+^), 887, and 861 (Mo–N).

[MoO_2_(L^4^)]_2_ (**4**): Yield: 46 mg, 37%. Anal. Calcd. for C_30_H_26_Mo_2_N_6_O_10_ (822.440): C, 43.81; H, 3.19; N, 10.22. Found: C, 43.58; H, 2.89; N, 10.05%. TG: calcd. for MoO_3_, 35.00%, found 34.74%. Selected IR data (cm^−1^): 3334, 3257 (NH_2_), 1613, 1602 (C=N), 1324 (C–O_hydrazone_), 1266 (C–O_phenolate_), 919, 908, (MoO_2_^2+^), 887, and 868 (Mo–N).

### 3.5. Synthesis of ***1a***–***4a***

Hydrazone H_2_L^1–4^ (0.083 g H_2_L^1,2^ or 0.087g H_2_L^3,4^; 0.31 mmol) was dissolved in 20 mL methanol and [MoO_2_(acac)_2_] (0.1 g, 0.31 mmol) was added to the resulting solution. In the reaction with H_2_L^1,2^, the reaction mixture was refluxed for two hours, whereas in the case of H_2_L^3,4^, it was heated at 40 °C. The resulting red product was collected via filtration and washed with a small amount of cold methanol. 

[MoO_2_(L^1^)(MeOH)] (**1a**). Yield: 85 mg, 64%. Anal. Calcd. for C_15_H_15_MoN_3_O_6_ (429.241): C, 41.97; H, 3.52; N, 9.79. Found C, 41.72; H, 3.29; N, 9.63%. TG: calcd. for MoO_3_, 33.53%, found: 33.42%; calcd. for CH_3_OH, 7.46%, found: 7.34%. Selected IR data (cm^−1^): 1621 (C=N)_py_, 1601 (C=N), 1341 (C–O_hydrazone_), 1275 (C–O_phenolate_), 1117 (C–O_MeOH_), 925, 913 (MoO_2_), and 897(O=Mo–O_MeOH_).

[MoO_2_(L^2^)(MeOH)] (**2a**). Yield: 108 mg, 82%. Anal. Calcd. for C_15_H_15_MoN_3_O_6_ (429.241): C, 41.97; H, 3.52; N, 9.79. Found C, 41.76; H, 3.30; N, 9.65%. TG: calcd. for MoO_3_, 33.53%, found: 33.17%; calcd. for CH_3_OH, 7.46%, found: 7.62%. Selected IR data (cm^−1^): 1620 (C=N)_py_, 1607 (C=N), 1333 (C–O_hydrazone_), 1267 (C–O_phenolate_), 1115 (C–O_MeOH_), 923, 913 (MoO_2_), and 900 (O=Mo–O_MeOH_).

[MoO_2_(L^3^)(MeOH)] (**3a**). Yield: 120 mg, 89%. Anal. Calcd. for C_16_H_17_MoN_3_O_6_ (443.262): C, 43.35; H, 3.87; N, 9.48. Found: C, 43.18; H, 3.66; N, 9.28%. TG: calcd. for MoO_3_, 32.47%, found: 32.65%; calcd. for CH_3_OH, 7.23%, found 6.89%. Selected IR data (cm^−1^): 3344, 3267 (NH_2_), 1611, 1599 (C=N), 1328 (C–O_hydrazone_), 1263 (C–O_phenolate_), 1163 (C–O_MeOH_), 926, 914 (MoO_2_^2+^), 895, and 874 (O=Mo–O_MeOH_).

[MoO_2_(L^4^)(MeOH)] (**4a**). Yield: 110 mg, 81%. Anal. Calcd. for C_16_H_17_MoN_3_O_6_ (443.262): C, 43.35; H, 3.87; N, 9.48. Found: C, 43.12; H, 3.63; N, 9.24%. TG: calcd. for MoO_3_, 32.47%, found 32.69%; calcd. for CH_3_OH, 7.23%, found 6.95%. Selected IR data (cm^−1^): 3456, 3356 (NH_2_), 1628, 1600 (C=N), 1321 (C–O_hydrazone_), 1275 (C–O_phenolate_), 1155 (C–O_MeOH_), 923, 914 (MoO_2_^2+^), 896, and 868 (O=Mo–O_MeOH_).

### 3.6. Physical Methods

Thermogravimetric (TG) analyses were carried out using a Mettler-Toledo TGA/SDTA851e thermobalance (Mettler Toledo, Columbus, OH, USA) with alumina crucibles under oxygen flow (10 mL min^−1^) in temperatures ranging from 25 °C to 600 °C at a heating rate of 10 °C min^−1^. Differential scanning calorimetry (DSC) measurements were performed under a nitrogen stream (10 mL min^−1^) on a TA Discovery DSC 25 (New Castle, DE, USA) instrument in temperatures ranging from 25 to 400 °C using Tzero aluminium pans and lids. Heating rates of 10 K min^−1^ were used for all investigations. The results of TG and DSC measurements were evaluated using the Mettler STAR^e^ Software (version 16.10) and TA Instruments Trios (v5.1.1.46572), respectively.

^1^H, ^13^C, and 2D NMR spectra were acquired at 298 K using a Bruker Avance III HD 400 spectrometer (Bruker Corporation, Karlsruhe, Germany) operating at 400 MHz. The spectra were recorded in DMSO-*d*_6_ with a sample concentration of 20 mg mL^−1^ using TMS as an internal standard. The Bruker Topspin™ software 4.0.6. was used for data analysis and signal integrations. 

Attenuated Total Reflectance Infrared (ATR-IR) spectra were acquired using a Perkin Elmer Spectrum Two spectrometer (Waltham, MA, USA).

The powder X-ray diffraction (PXRD) for qualitative phase analysis data was collected using a Malvern Panalytical Aeris diffractometer (Malvern Panalytical B.V., Almelo, The Netherlands) in the Bragg-Brentano geometry with a PIXcel^1D^ detector using Cu*K_α_* radiation (*λ* = 1.5406 Å). Samples were then placed on a Si sample holder. Powder patterns were collected at room temperature in the range of 5–40° (2*θ*) and visualized utilizing the Malvern Panalytical HighScore Software Suite (version 4.8.) [49].

Single crystals of ligands H_2_L^3^ and H_2_L^4^ and complexes **1a**, **2a**, **3a**, and **3** of suitable quality were selected for the diffraction experiments. Data were collected using *ω*-scans: (1) for ligands H_2_L^3^ and H_2_L^4^ on an Oxford Xcalibur diffractometer (Oxford Diffraction Ltd., Abingdon, UK) equipped with a 4-circle kappa geometry goniometer, CCD Sapphire 3 detector and graphite-monochromated Mo K_α_ radiation (*λ* = 0.71073 Å) at 150(2) K, (2) for complexes **1a**, **2a**, **3a**, and **3** on a Rigaku XtaLAB Synergy-S diffractometer were armed with a Dualflex source (Cu *K*α radiation, *λ* = 1.54184 Å) and a HyPix detector at 170 K. The data analysis was conducted using the CrysAlis software suite (version 1.171.43.90) [50]. Detailed crystallographic information, including data acquisition and structural refinement specifics, can be found in the Supplementary Materials, specifically in Tables S1 and S5. Structure solutions were derived using the dual-space approach with the aid of SHELXT [51]. Refinement was executed through a least-squares method on *F*^2^, encompassing all reflections and anisotropic displacement parameters for every non-H atom. The hydrogen atoms linked to carbon were set in idealized positions and adjusted based on the riding model. Their *U*_iso_ values were set at 1.2 times the *U*_eq_ of the respective carbon atoms, or at 1.5 times for CH_3_ groups. Hydrogen atoms connected to other atoms were initially positioned using a Fourier map during the final refinement phase, with their positions being refined freely while maintaining fixed N–H and O–H bond lengths at 0.86(2) and 0.82(2) Å, respectively. All refinement processes utilized SHELXL [52], operated within the Olex2 suite [53]. The generation of molecular graphics and geometric analysis was carried out using Mercury [41]. 

Agilent 7820A chromatograph, equipped with an FID detector and an HP5-MS capillary column (30 m × 0.32 mm × 0.25 µm), was employed to generate chromatograms. The GC parameters were assessed using authentic samples of reactants and products. The conversion of olefins and the formation of the corresponding epoxides were determined based on calibration curves, with acetophenone as the internal standard.

### 3.7. Theoretical Calculations

The geometries of the investigated species were optimized using the DFT approach with Gaussian 09 rev. D01 program suite [54] using the B3LYP three-parameter functional [55,56,57] in conjunction with the 6-31G* basis set [58,59,60,61] for light atoms (O, N, C, H) and the CEP-31G set for Mo atoms [62,63]. The geometries of the complexes and intermediates were optimized using geometries inspired by X-ray diffraction. All coordinates are listed in the Supplementary Materials (Table S14). The frequency analysis confirmed that the optimized geometries were local minima. The full optimization of the transition states was guided by a preliminary scan of the relevant internal coordinate. By frequency analyses, all optimized geometries were confirmed to be stationary points and local minima for stable molecules or reaction intermediates and first-order saddle points for TSs. For TSs, the analysis of the imaginary frequency confirmed the expected motion along the chosen reaction coordinate. These values and related schemes are shown in Table S13. The calculated frequencies were also used to obtain the thermochemical parameters at 298 K according to the standard approximations (ideal gas, rigid rotor, and harmonic oscillator).

## 4. Conclusions

The structural formations of molybdenum complexes can vary based on the specific hydrazide employed. Depending on this hydrazide moiety (aminobenzohydrazide vs. (iso)nicotinic hydrazide), structural assemblies such as dinuclear or polynuclear molybdenum complexes can be attained. Furthermore, the selection of the solvent plays a pivotal role as it determines whether a mononuclear (from methanol) or dinuclear/polynuclear (from dichloromethane) complex can be isolated. The catalytic performances of the synthesized complexes were evaluated under environmentally friendly green chemistry conditions. All the examined compounds exhibited remarkable catalytic capabilities for cyclooctene conversion and selectivity toward epoxide production. Moreover, dinuclear compounds demonstrated a superior rate of conversion to catalytically active species compared to polynuclear catalysts. Conversely, mononuclear catalysts derived from (iso)nicotinic hydrazide displayed a higher propensity for conversion into the pentacoordinated active species compared to complexes derived from aminobenzohydrazides. Future investigations will focus on the catalytic oxidations of bio-derived substrates, particularly emphasizing the exploration of dinuclear catalysts.

## Figures and Tables

**Scheme 3:**
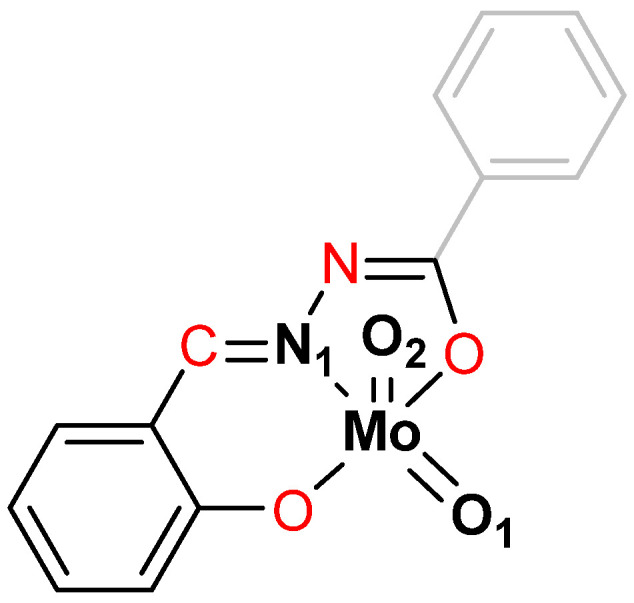
Representation of the MoO_2_L complex with a coordination sphere, highlighting the considered atoms taken into consideration for bond lengths (bold black) and for dihedral angles (red). The O_β_ is shown in Scheme 2.

**Table 1:** Catalytic results of *cis*-cyclooctene epoxidation. Reaction conditions: time, 6 h; temperature, 80 °C, *n*(catalyst)/*n*(cyclooctene)/*n*(oxidant) = 0.05 mmol/20 mmol/40 mmol.

Catalyst	Con. ^a^/%	Sel. ^b^/%	TON ^c^	TOF_20min_^d^
**1**	89	78	348	191
**2**	90	84	360	91
**3**	99	85	399	415
**4**	67	90	180	279
**1a**	89	89	359	350
**2a**	97	85	389	356
**3a**	99	92	395	214
**4a**	85	98	341	191

^a^ cyclooctene consumed at the end of the reaction. ^b^ Formed epoxide per converted olefin at the end of the reaction. ^c^ *n*(cyclooctene) transformed/*n*(catalyst)/time(h) at 20 min. ^d^ *n*(cyclooctene) transformed/*n*(catalyst) at the end of reaction.

**Table 2:** Comparison of the catalytic parameters (conversion, selectivity, TOF_20min_, and TON) or the previously published catalysts.

Catalyst	Con./%	Sel./%	TON	TOF_20min_	Ref.
[MoO_2_(L^2′^)]*_n_*	27	56	113	72	[46]
[MoO_2_(L^2″^)]*_n_*	49	67	192	119	[46]
][MoO_2_(L^3′^)]_2_	90	92	372	343	[33]
[MoO_2_(L^3″^)(MeOH)]	85	90	349	383	[33]
[MoO_2_(L^3″^)]_2_	87	91	330	345	[33]
[MoO_2_(L^4′^)(MeOH)]	56	86	229	75	[34]
[MoO_2_(L^4″^)(MeOH)]	63	85	257	97	[34]
[MoO_2_(L^4′^)]_2_	84	90	380	295	[34]
[MoO_2_(L^4″^)]_2_	89	86	386	298	[34]

**Table 3:** Enthalpy values (Δ*H*) (in kcal mol^−1^) for each step depicted in Scheme 2 (n.c. = non-calculated).

		Absolute Enthalpy (Relative) Values			
	Steps	[MoO_2_(L^1^)]	[MoO_2_(L^2^)]	[MoO_2_(L^3^)]	[MoO_2_(L^4^)]
(0)	Dimer decoordination	n.c.	n.c.	5.9	5.5
(0)	MeOH decoordination	10.6	11.1	10.8	10.3
(I)	TBHP coordination	−8.7	−8.7	−8.4	−8.9
(II)	TS barrier	23.5	23.2	23.8	24.6
(III)	Release of epoxide	−51.3	−51.1	−51.4	−51.7
(IV)	Regeneration of catalyst	−4.9	−4.9	−5.4	−5.4

**Table 4:** Interatomic distances (Å) and dihedral angle (°) for the calculated structures [MoO_2_L^i^(D)] (i = 1–4) and related TS according to Scheme 3.

		[MoO_2_L(D)]			
D		MeOH	no	TBHP	TS
Interatomic distances (Å)					
[Mo–O_β_]	L^1^	2.552	-	2.990	2.394
	L^2^	2.499	-	2.993	2.397
	L^3^	2.504	-	3.018	2.412
	L^4^	2.509	-	3.041	2.428
N^1^–Mo	L^1^	2.283	2.274	2.268	2.278
	L^2^	2.296	2.274	2.269	2.278
	L^3^	2.293	2.273	2.268	2.275
	L^4^	2.294	2.275	2.265	2.273
Mo=O^1^ (±plane)	L^1^	1.709	1.697	1.714	1.733
	L^2^	1.705	1.697	1.714	1.733
	L^3^	1.706	1.698	1.715	1.734
	L^4^	1.708	1.699	1.716	1.735
Mo–O^2^ (⊥plane)	L^1^	1.694	1.696	1.691	1.693
	L^2^	1.695	1.696	1.691	1.693
	L^3^	1.696	1.697	1.692	1.693
	L^4^	1.696	1.697	1.693	1.695
Interatomic angle (absolute values in °)					
OCNO Dihedral angle	L^1^	9.99	6.16	11.05	15.69
	L^2^	10.36	6.28	11.18	17.10
	L^3^	10.45	6.05	11.99	16.39
	L^4^	10.63	6.10	12.19	15.71

## Data Availability

Crystallographic data sets for the structures H_2_L^3^, H_2_L^4^, **1a**, **2a**, **3a,** and **3** are available through the Cambridge Structural Database with deposition numbers CCDC 2310499-2310504. These data can be obtained free of charge at https://www.ccdc.cam.ac.uk/conts/retrieving.html (or from the CCDC, 12 Union Road, Cambridge CB2 1EZ, UK; Fax: +44-1223-336033; E-mail: deposit@ccdc.cam.ac.uk) (accessed on 27 November 2023).

## Supplementary Materials

The supporting information can be downloaded at: https://www.mdpi.com/article/10.3390/ijms25031503/s1.
