# Supplementary material for: Preparative and Catalytic Properties of MoVI Mononuclear and Metallosupramolecular Coordination Assemblies Bearing Hydrazonato Ligands
Source: Int J Mol Sci. 2024 Jan 25;25(3):1503. doi: 10.3390/ijms25031503 (PMC10855701; doi:10.3390/ijms25031503)
Supplement: Supplementary file 1 [file ijms-25-01503-s001.zip › ijms-2813772-supplementary.pdf]

Supplementary Materials

Preparative and catalytic properties of Mo^{VI} mononuclear and metallosupramolecular coordination assemblies bearing hydrazonato ligands

Mirna Mandarić, Edi Topić, Dominique Agustin, Jana Pisk, Višnja Vrdoljak*

Contents

Scheme 2
X-ray diffraction.. 3
IR-ATR spectra 22
NMR spectroscopy.. 25
Imaginary frequency (cm^{-1}) of the calculated transition states shown in Table S14.............. 33
DFT Coordinates of all species. 33

Scheme

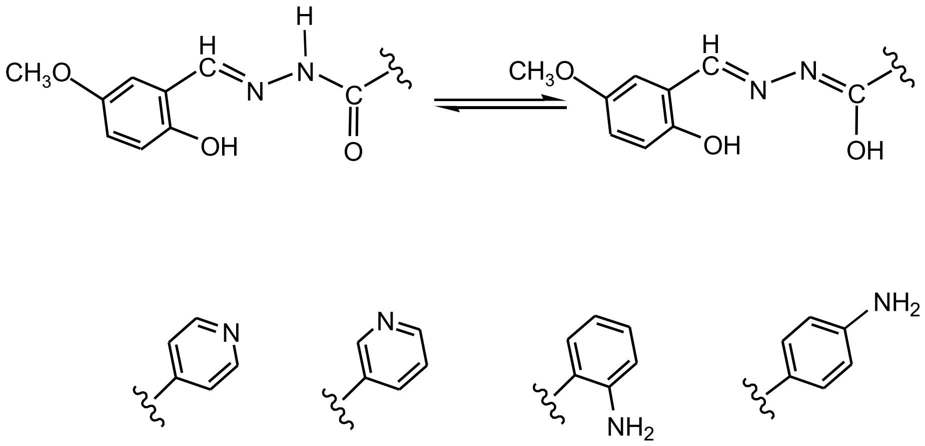

Scheme S1. Hydrazidato and hydrazonato tautomeric forms.

X-ray diffraction

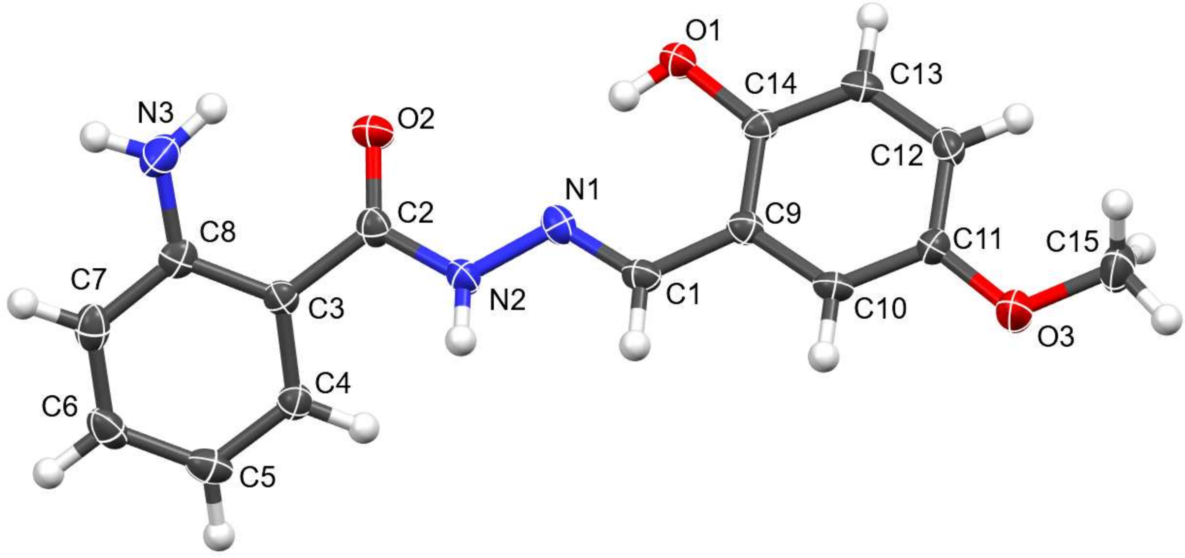

(a)

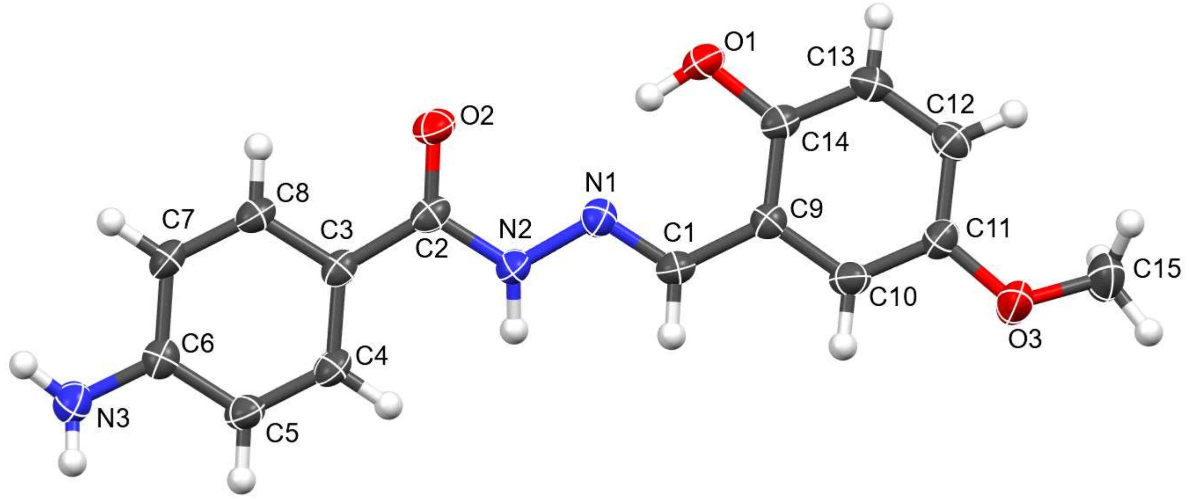

(b)

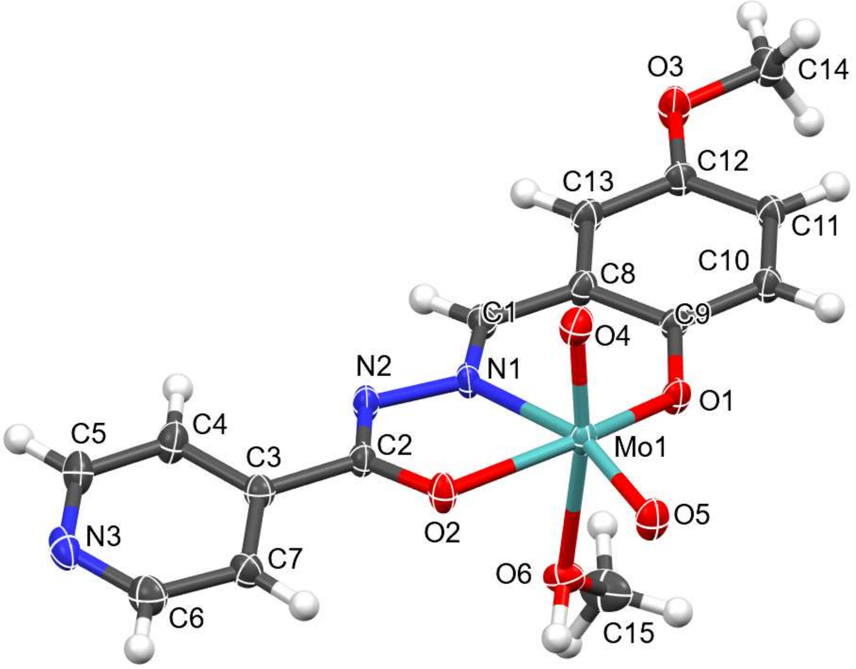
O3
C14
C12
C13
C11
C10
C8
C1
C9
O4
N2
N1
O1
C5
C4
Mo1
C3
C2
N3
O5
O2
C7
O6
C15
C6

(c)

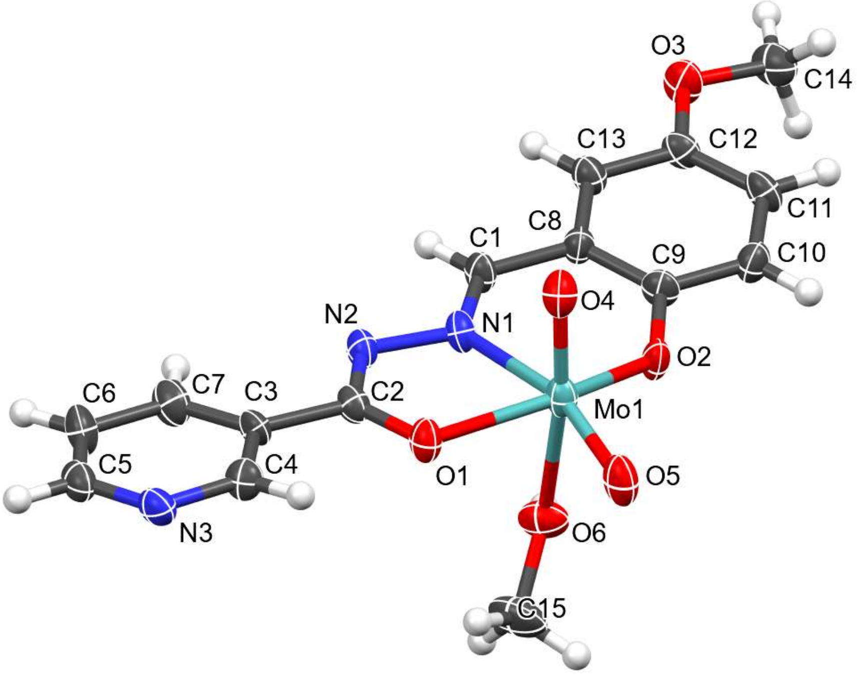
O3
C14
C13
C12
C11
C1
C8
C9
C10
N2
O4
N1
O2
C6
C7
C3
C2
Mo1
C5
C4
O1
O5
N3
O6
C15

(d)

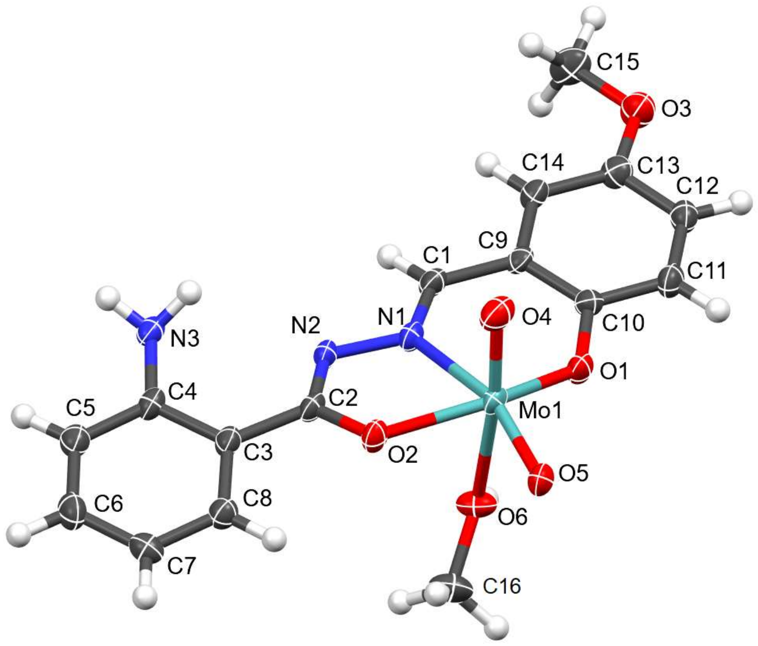

(e)

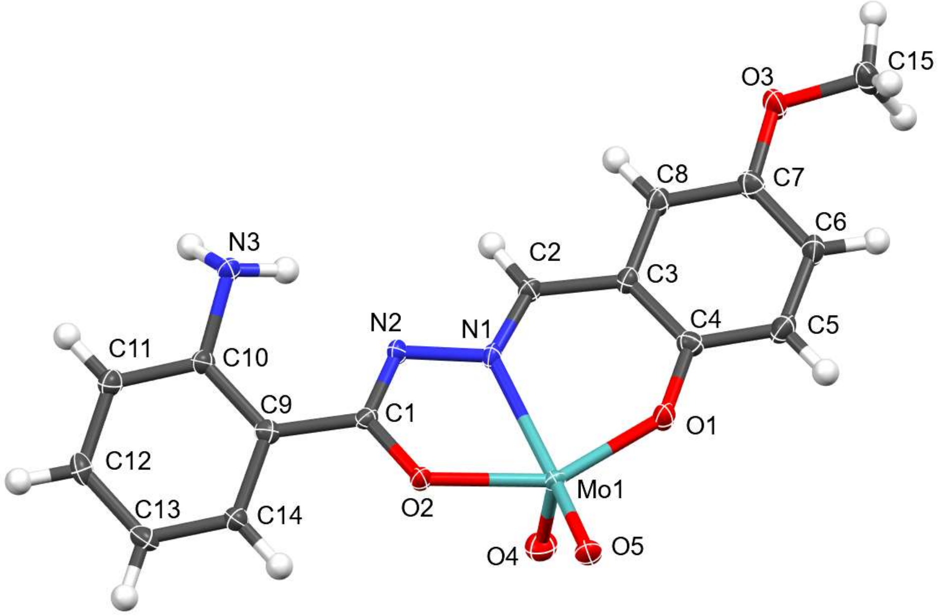

(f)

Figure S1. Molecular structure and enumeration scheme for **(a) H_2L^3, (b) H_2L^4, (c) 1a, (d) 2a, (e) 3a, (f) 3.** Non-hydrogen atoms are shown as thermal ellipsoids at 30% probability. The second symmetrically inequivalent molecule in (b) is omitted for clarity. In (f), only symmetrically unique molecular fragment is shown.

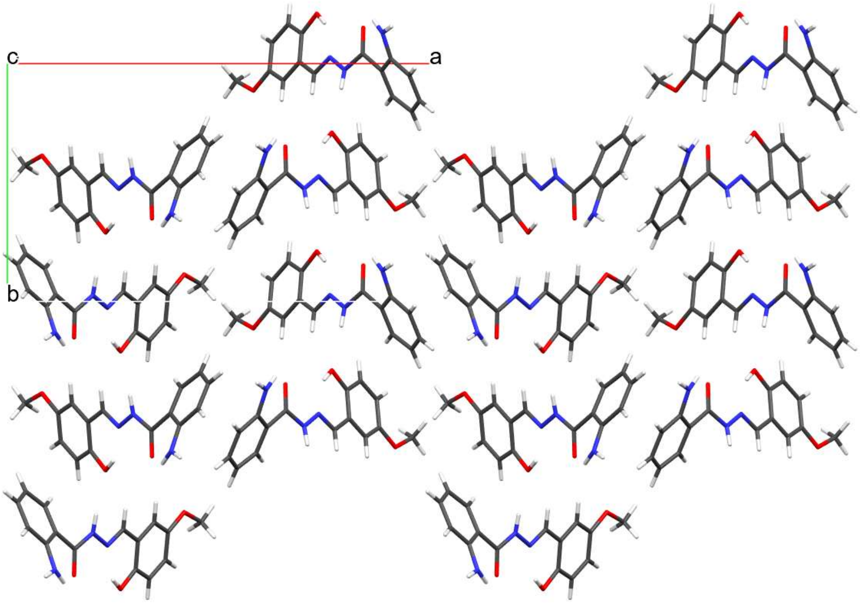

(a)

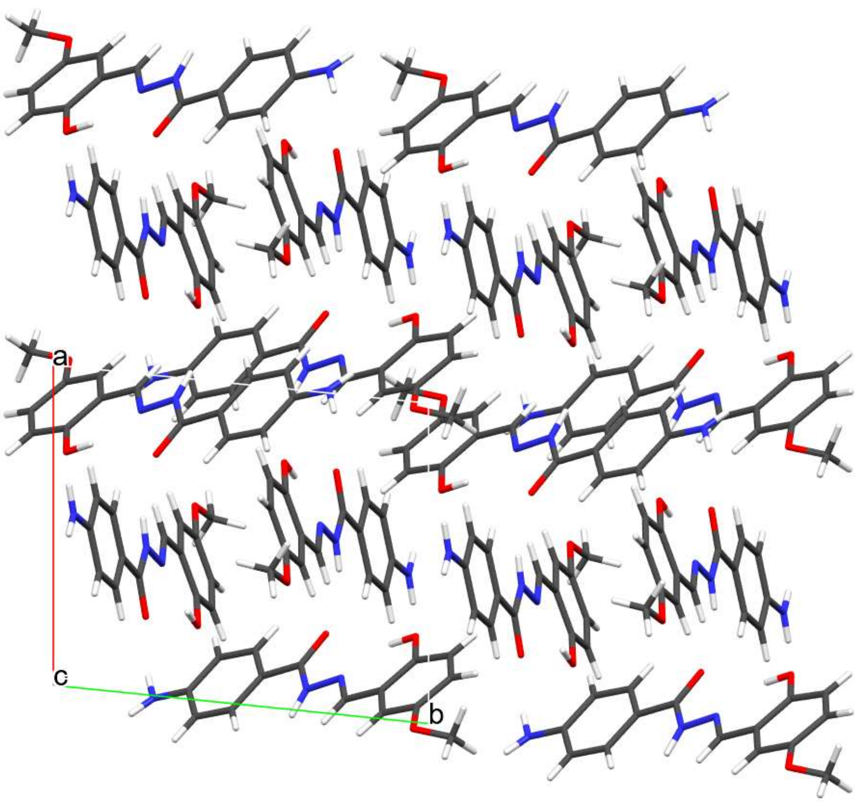

(b)

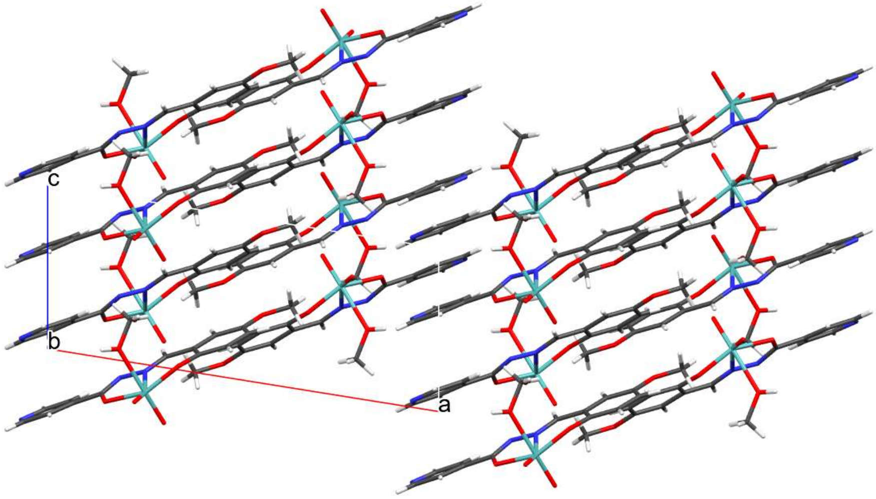

(c)

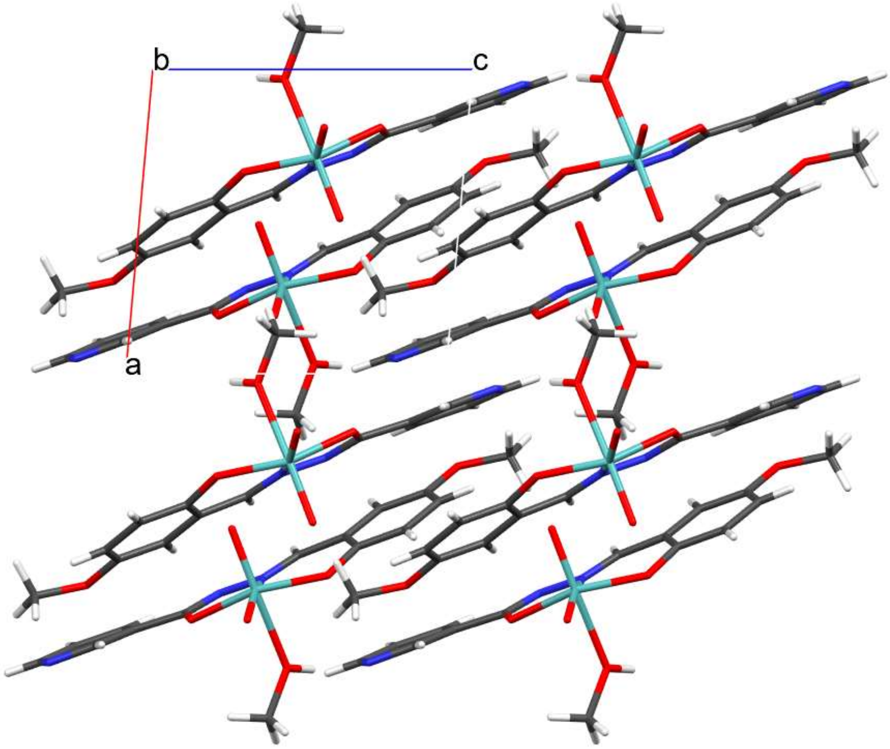

(d)

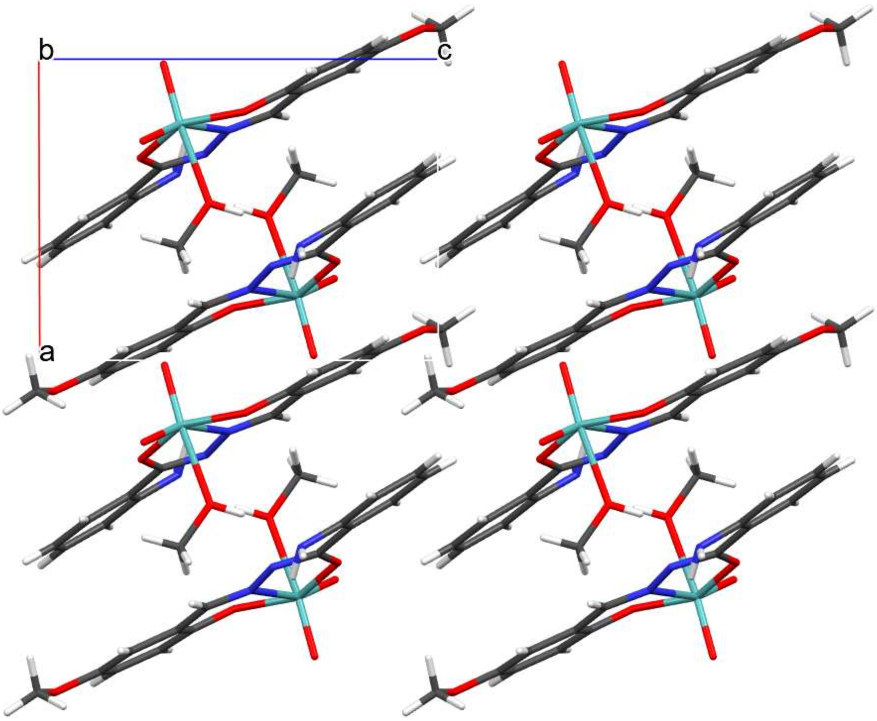

(e)

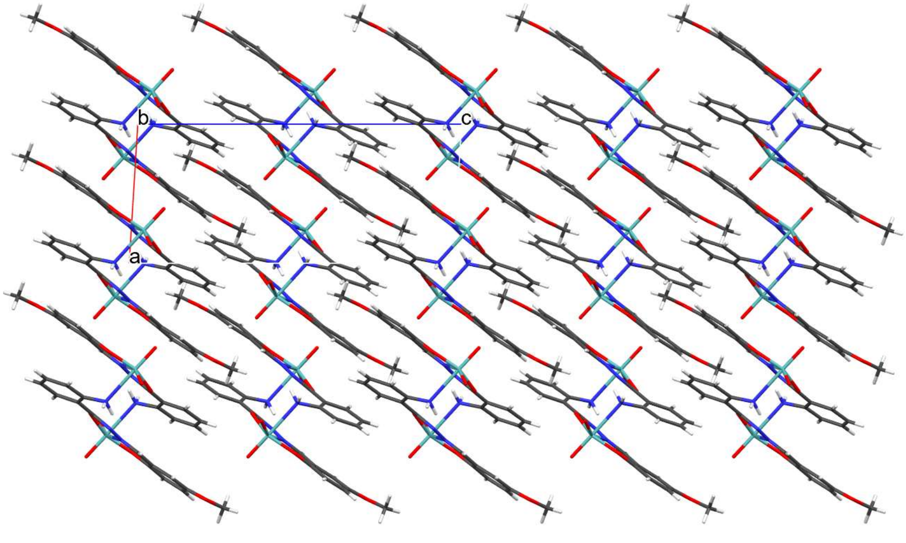

(f)

Figure S2. Packing of molecules in the crystal structure of **(a) H_2L^3, (b) H_2L^4, (c) 1a, (d) 2a, (e) 3a, (f) 3**, shown along *c*-axis for ligands (a) and (b) and along *b*-axis for complexes (c)-(f).

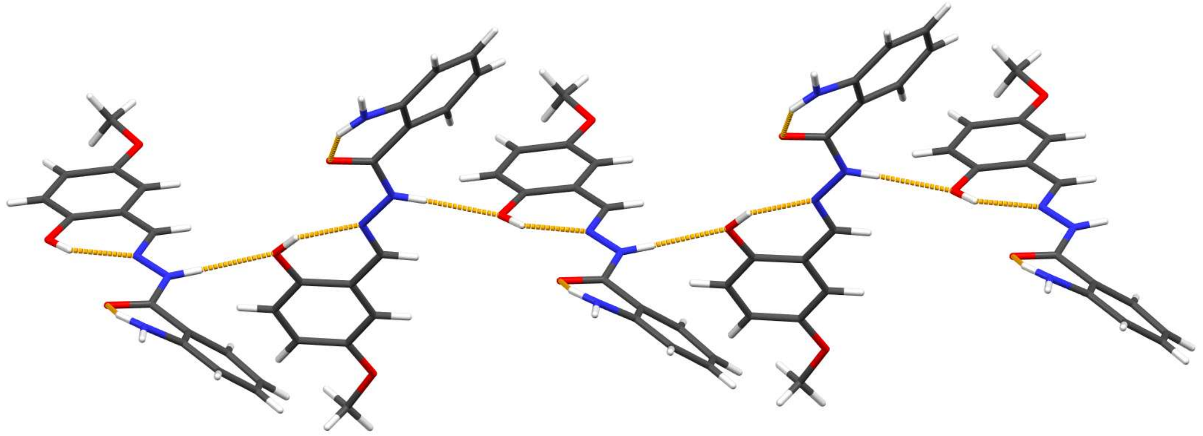

(a)

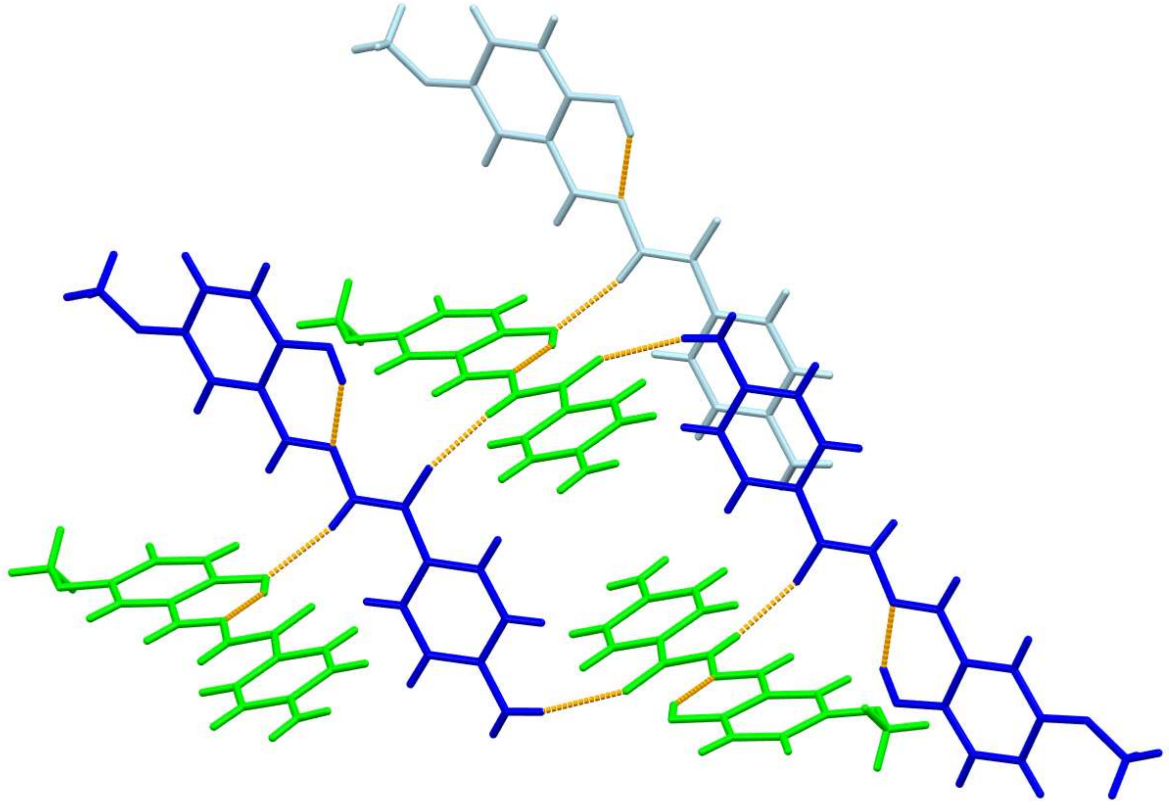

(b)

Figure S3. Supramolecular environment of ligand molecules in **(a) H_2L^3** and **(b) H_2L^4**. In (a), ligand molecules form supramolecular chains through hydrazide N–H···O_{ar} hydrogen bond. In (b), symmetrically inequivalent molecules (shown as green and blue) form a complicated hydrogen bond network.

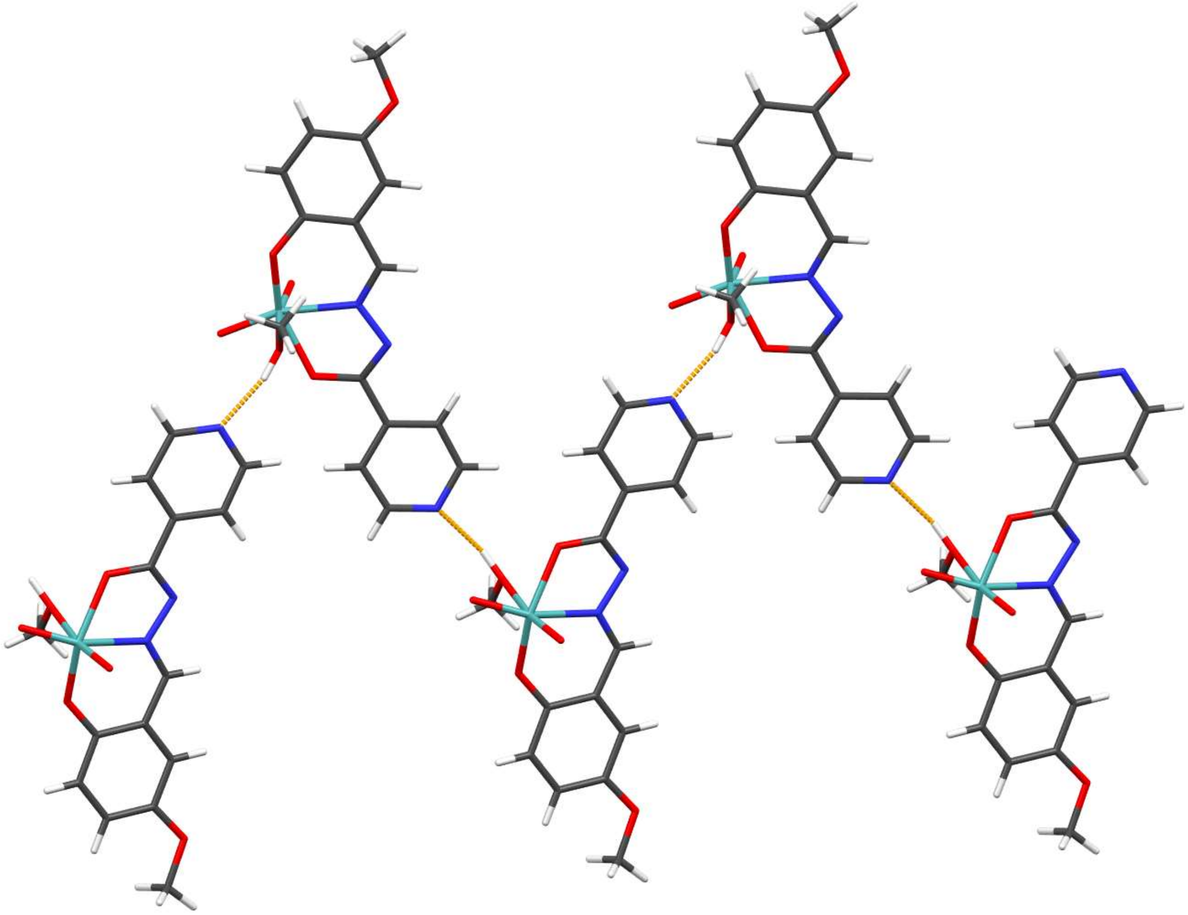

(a)

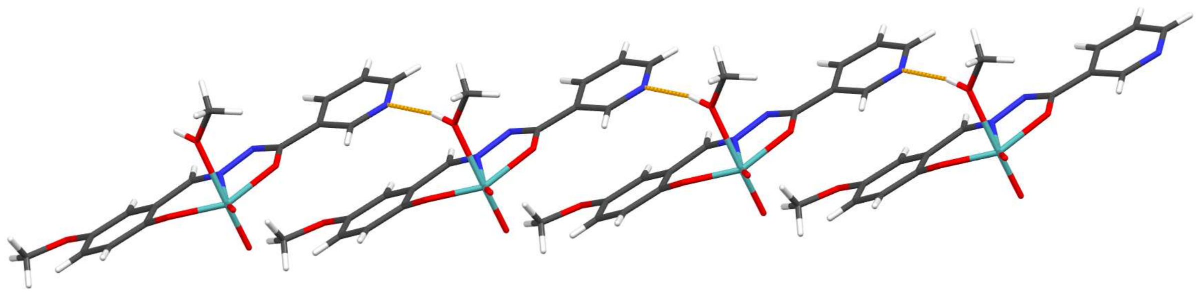

(b)

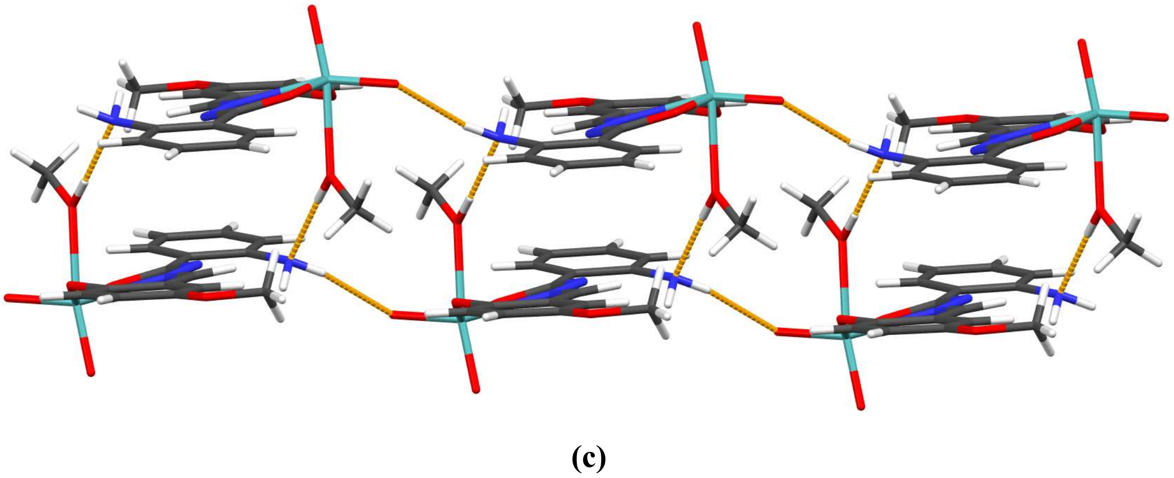

(c)

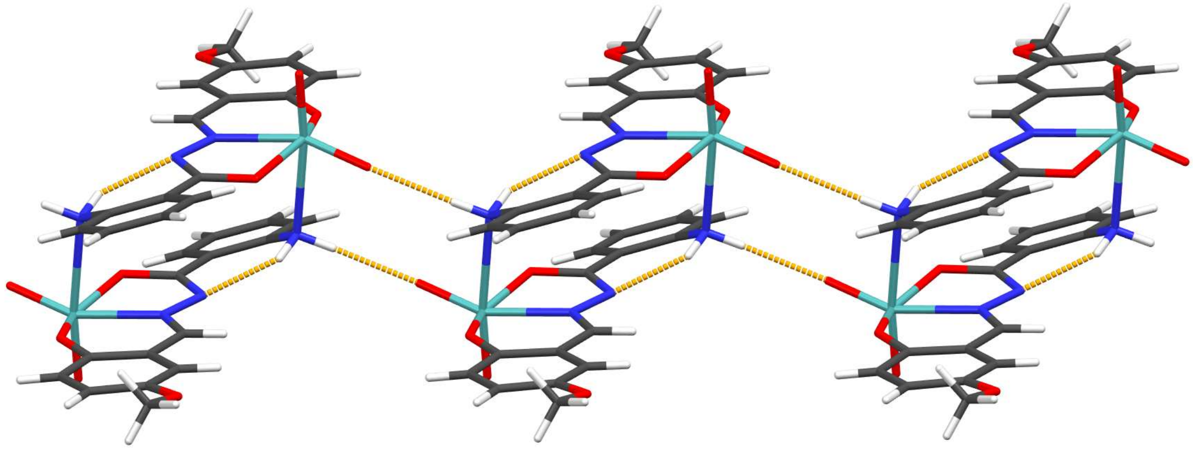

(d)

Figure S4. Supramolecular environment of complex molecules in **(a) 1a, (b) 2a, (c) 3a**, and **(d) 3**. All complexes have few available hydrogen bond donors (methanol OH moiety or NH2 moiety on the aromatic ring), therefore observed supramolecular networks are relatively simple. In (a) and (b) supramolecular chains are formed between methanol OH group and pyridyl nitrogen atom. In (c), supramolecular dimers are established through methanol OH and amine nitrogen atom, which are further connected in chains through N-H···O_{eq}=Mo hydrogen bond. Finally, in (d), only the similar situation is found as in (c), except that dimers are formed through coordination of amine to the MoO_2 core.

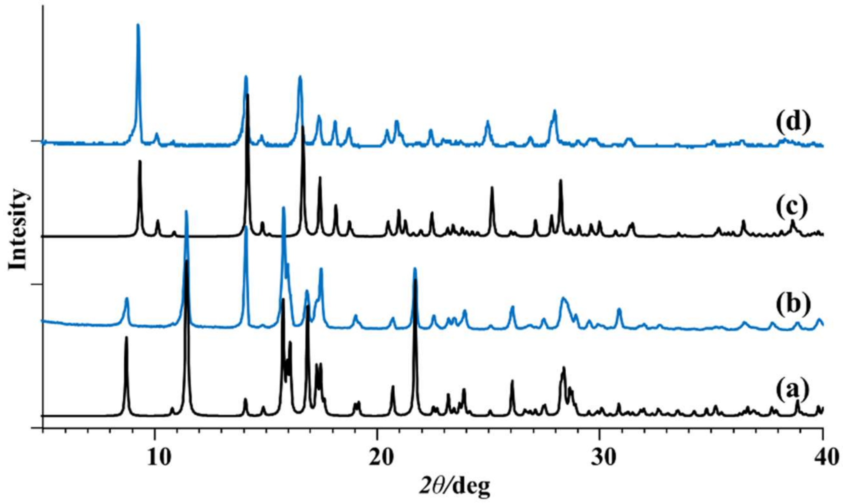

Figure S5. Comparison of the measured PXRD patterns (blue) and patterns calculated from the X-ray single-crystal structures (black): (a and b) $[MoO_2(L^1)]_n$, (c and d) $[MoO_2(L^3)]_2$.

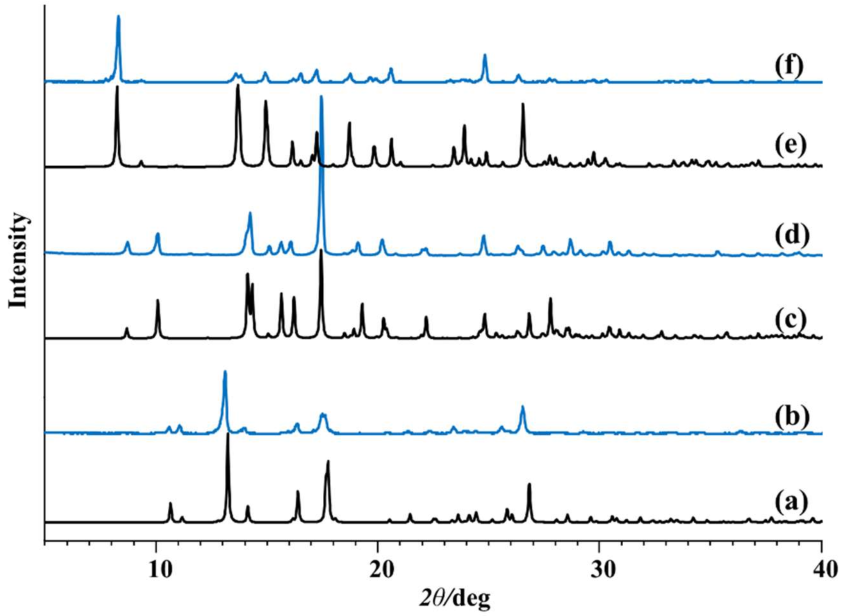

Figure S6. Comparison of the measured PXRD patterns (blue) and patterns calculated from the X-ray single-crystal structures (black): (a and b) $MoO_2(L^1)(MeOH)]$, (c and d) $[MoO_2(L^2)(MeOH)]$, and (e and f) $[MoO_2(L^3)(MeOH)]$.

Table S1. Experimental and crystallographic data for ligands $\mathbf{H_2L^3}$ and $\mathbf{H_2L^4}$.

Identifier	$\mathbf{H_2L^3}$	$\mathbf{H_2L^4}$
Empirical formula	$C_{15}H_{15}N_3O_3$	$C_{15}H_{15}N_3O_3$
M_r	285.30	285.30
T/K	150.00	150.00
Crystal system	orthorhombic, yellow block	triclinic, yellow prism
Space group	$P\ n\ a\ 2_1$	$P\ –1$
a/Å	19.964(2)	9.6923(6)
b/Å	11.2493(9)	11.4322(6)
c/Å	6.1009(8)	12.8069(7)
α/°	90	95.232(4)
β/°	90	93.942(5)
γ/°	90	95.427(4)
V/Å^3	1370.1(3)	1402.52(14)
Z	4	4
ρ_{calc}/g cm^{-3}	1.383	1.351
μ/mm^{-1}	0.099	0.096
$F(000)$	600	600
Crystal size/mm^3	0.7×0.2×0.02	0.4×0.2×0.2
Radiation	MoKα (λ = 0.71073Å)	MoKα (λ = 0.71073Å)
2Θ range/°	8.628 to 51.982	8.284 to 51.998
Index ranges	$-24 \leq h \leq 24$,<br>$-13 \leq k \leq 13$,<br>$-7 \leq l \leq 7$	$-7 \leq h \leq 11$,<br>$-14 \leq k \leq 14$,<br>$-15 \leq l \leq 15$
Reflections collected	12063	12837
Independent reflections	2645 [R_{int} = 6.2%, R_{sigma} = 6.71 %]	5486 [R_{int} = 5.3%, R_{sigma} = 3.11 %]
Data/restraints/parameters	2645/-/203	5486/-/405
g_1, g_2 in w^a	0.0313, 0	0.0546, 0
Goodness–of–fit on F^2, S^b	1.069	1.008
Final R and wR^c values [$I \geq 2\sigma(I)$]	R_1 = 5.29%, wR_2 = 8.4%	R_1 = 4.92%, wR_2 = 10.86%
Final R and wR^c values [all data]	R_1 = 7.08%, wR_2 = 8.95%	R_1 = 8.0%, wR_2 = 12.55%
Largest diff. peak/hole / e Å^{-3}	0.147/–0.155	0.206/–0.211
Flack parameter	0.1(10)	/

$^a w = 1/[\sigma F_o^2 + (g_1P)^2 + g_2P]$ where $P = (F_o^2 + 2F_c^2)/3$

$^b S = \{\Sigma[w(F_o^2 - F_c^2)2]/(N_r - N_p)\}^{1/2}$ where N_r = number of independent reflections, N_p = number of refined parameters.

$^c R = \Sigma||F_o| - |F_c|/\Sigma|F_o|$; $wR = \{\Sigma[w(F_o^2 - F_c^2)2]/\Sigma[w(F_o^2)2]\}^{1/2}$

Table S2. Selected bond lengths in the crystal structures of ligands $\mathbf{H_2L^3}$ and $\mathbf{H_2L^4}$.

$\mathbf{H_2L^3}$					
Atoms	Bond length/Å	Atoms	Bond length/Å	Atoms	Bond length/Å
C4–C5	1.382(5)	C11–C12	1.386(5)	N3–H3B	0.85(3)
C5–C6	1.394(6)	N1–C1	1.290(4)	O1–C14	1.378(4)
C6–C7	1.373(5)	N1–N2	1.375(4)	O1–H1	0.83(4)
C7–C8	1.408(5)	N2–C2	1.374(4)	O2–C2	1.233(4)
C9–C10	1.398(5)	N2–H2	0.88(2)	O3–C11	1.384(4)
C9–C14	1.410(5)	N3–C8	1.371(5)	O3–C15	1.420(5)
C10–C11	1.381(5)	N3–H3A	0.89(4)		
$\mathbf{H_2L^4}$					
Atoms	Bond length/Å	Atoms	Bond length/Å	Atoms	Bond length/Å
C4–C5	1.376(3)	C25–C26	1.388(2)	N6–H6A	0.87(2)
C5–C6	1.394(3)	C26–C27	1.386(3)	N6–H6B	0.859(18)
C6–C7	1.394(3)	N1–C1	1.279(2)	O1–C14	1.365(2)
C7–C8	1.376(3)	N1–N2	1.378(2)	O1–H1	0.904(19)
C9–C10	1.393(2)	N2–C2	1.370(2)	O2–C2	1.231(2)
C9–C14	1.406(3)	N2–H2	0.906(19)	O3–C11	1.376(2)
C10–C11	1.383(3)	N3–C6	1.381(2)	O3–C15	1.397(2)
C11–C12	1.389(3)	N3–H3A	0.872(19)	O4–C29	1.374(2)
C19–C20	1.381(3)	N3–H3B	0.89(2)	O4–H4	0.849(19)
C20–C21	1.394(3)	N4–C16	1.282(2)	O5–C17	1.238(2)
C21–C22	1.395(3)	N4–N5	1.375(2)	O6–C26	1.370(2)
C22–C23	1.375(2)	N5–C17	1.367(2)	O6–C30	1.426(2)
C24–C25	1.392(2)	N5–H5	0.847(17)		

Table S3. Selected bond angles in the crystal structures of ligands $\mathbf{H_2L^3}$ and $\mathbf{H_2L^4}$.

**H_2L^3**					
Atoms	Bond angle/°	Atoms	Bond angle/°	Atoms	Bond angle/°
N1–C1–C9	120.6(3)	N3–C8–C3	121.7(3)	N2–N1–C1	117.5(2)
N2–C2–C3	114.4(3)	N3–C8–C7	119.9(3)	C2–N2–H2	121(3)
O2–C2–C3	123.9(3)	C1–C9–C10	118.6(3)	N1–N2–C2	119.4(2)
O2–C2–N2	121.7(3)	C1–C9–C14	122.5(3)	N1–N2–H2	119(3)
C2–C3–C4	120.2(3)	C10–C9–C14	118.9(3)	C8–N3–H3A	120(2)
C2–C3–C8	120.4(3)	C9–C10–C11	121.0(3)	C8–N3–H3B	114(2)
C4–C3–C8	119.2(3)	C10–C11–C12	120.0(3)	H3A–N3–H3B	121(3)
C3–C4–C5	121.3(3)	O3–C11–C10	116.0(3)	C14–O1–H1	106(2)
C4–C5–C6	119.3(3)	O3–C11–C12	124.0(3)	C11–O3–C15	117.5(3)
C5–C6–C7	120.6(3)	C11–C12–C13	119.7(3)		
C6–C7–C8	121.1(4)	C12–C13–C14	121.0(3)		
C3–C8–C7	118.3(3)	O1–C14–C9	122.9(3)		
**H_2L^4**					
Atoms	Bond angle/°	Atoms	Bond angle/°	Atoms	Bond angle/°
N1–C1–C9	120.61(16)	O1–C14–C9	121.99(16)	C27–C28–C29	121.25(18)
N2–C2–C3	117.03(16)	N4–C16–C24	120.02(17)	O4–C29–C24	121.88(15)
O2–C2–C3	122.84(16)	N5–C17–C18	116.57(17)	N2–N1–C1	117.30(15)
O2–C2–N2	120.10(16)	O5–C17–C18	123.06(16)	C2–N2–H2	122.8(13)
C2–C3–C4	124.96(16)	O5–C17–N5	120.37(16)	N1–N2–C2	117.91(15)
C2–C3–C8	117.16(16)	C17–C18–C19	125.24(16)	N1–N2–H2	118.7(13)
C4–C3–C8	117.85(16)	C17–C18–C23	117.18(17)	C6–N3–H3A	115.7(13)
C3–C4–C5	121.17(17)	C19–C18–C23	117.58(16)	C6–N3–H3B	116.0(14)
C4–C5–C6	120.65(17)	C18–C19–C20	121.09(17)	H3A–N3–H3B	120.5(19)
C5–C6–C7	118.61(16)	C19–C20–C21	120.93(18)	N5–N4–C16	118.08(16)
N3–C6–C5	121.34(17)	C20–C21–C22	118.18(17)	C17–N5–H5	124.7(15)
N3–C6–C7	120.02(16)	N6–C21–C20	121.51(18)	N4–N5–C17	118.30(16)
C6–C7–C8	120.51(16)	N6–C21–C22	120.30(18)	N4–N5–H5	117.0(15)
C3–C8–C7	121.21(17)	C21–C22–C23	120.81(17)	C21–N6–H6A	118.0(13)
C1–C9–C10	118.40(16)	C18–C23–C22	121.41(18)	C21–N6–H6B	120.7(14)
C1–C9–C14	122.64(15)	C16–C24–C25	119.18(17)	H6A–N6–H6B	121.3(19)
C10–C9–C14	118.92(16)	C16–C24–C29	121.90(16)	C14–O1–H1	108.2(13)
C9–C10–C11	121.13(17)	C25–C24–C29	118.92(16)	C11–O3–C15	118.37(15)
C10–C11–C12	119.73(16)	C24–C25–C26	121.32(17)	C29–O4–H4	106.4(13)
O3–C11–C10	115.64(16)	C25–C26–C27	119.42(17)	C26–O6–C30	117.01(15)
O3–C11–C12	124.62(16)	O6–C26–C25	115.71(17)		

Table S4. Hydrogen bond parameters in the crystal structure of **H_2L^3** and **H_2L^4**

**H_2L^3**					
D–H···A	D-H	H···A	D···A	∠D–H···A	Symmetry code
N2–H2···O1	0.88(2)	2.19(2)	3.069(3)	174(4)	–3/2–*x*, –1/2+*y*, –1/2+*z*
C10–H10···O2	0.95	2.47	3.404(4)	167	–3/2–*x*, –1/2+*y*, 1/2+*z*
**H_2L^4**					
D–H···A	D-H	H···A	D···A	∠D–H···A	Symmetry code
N2–H2···O4	0.906(19)	2.077(19)	2.930(2)	156.4(18)	–1+*x*, *y*, *z*
N3–H3A···O3	0.872(19)	2.586(19)	3.160(2)	124.2(16)	–*x*, 1–*y*, 1–*z*
N3–H3B···O5	0.89(2)	2.208(19)	3.075(2)	166.0(18)	1–*x*, 1–*y*, 2–*z*
N5–H5···O2	0.847(17)	2.014(17)	2.823(2)	159(2)	*x*, *y*, *z*
N6–H6A···O1	0.87(2)	2.30(2)	3.121(2)	157.1(18)	1–*x*, 2–*y*, 2–*z*
C4–H4A···O4	0.95	2.59	3.445(2)	150	–1+*x*, *y*, *z*
C15–H15B···O5	0.98	2.40	3.310(3)	154	1–*x*, 2–*y*, 1–*z*
C15–H15C···O2	0.98	2.59	3.476(3)	151	–*x*, 2–*y*, 1–*z*
C16–H16···O2	0.95	2.52	3.238(2)	133	*x*, *y*, *z*
C19–H19···O2	0.95	2.55	3.444(2)	158	*x*, *y*, *z*

Table S5. Experimental and crystallographic data for dioxomolybdenum(VI) complexes **1a, 2a, 3a** and **3.**

Identifier	**1a**	**2a**	**3a**	**3**
Empirical formula	$C_{15}H_{15}MoN_3O_6$	$C_{15}H_{15}MoN_3O_6$	$C_{16}H_{17}MoN_3O_6$	$C_{30}H_{26}Mo_2N_6O_{10}$
M_r	429.24	429.24	443.26	822.45
T/K	169.98(10)	169.99(10)	169.99(10)	170.00(10)
Crystal system	monoclinic, orange needle	monoclinic, orange plate	triclinic, orange plate	monoclinic, red block
Space group	P 1 $2_1/c$ 1	P 1 2_1 1	P –1	P 1 $2_1/c$ 1
a/Å	17.7236(4)	7.9094(3)	8.1385(3)	8.06221(12)
b/Å	12.4615(2)	12.4768(4)	9.7144(3)	9.71973(14)
c/Å	7.32534(17)	8.2941(2)	10.9563(4)	18.7390(3)
α/°	90	90	78.178(3)	90
β/°	99.127(2)	95.092(3)	88.831(3)	93.5334(14)
γ/°	90	90	85.144(3)	90
V/Å^3	1597.41(6)	815.26(5)	844.79(5)	1465.64(4)
Z	4	2	2	2
ρ_{calc}/g cm^{-3}	1.785	1.749	1.743	1.864
μ/mm^{-1}	7.087	6.943	6.722	7.641
F(000)	864	432	448	824
Crystal size/mm^3	0.15×0.1×0.05	0.48×0.14×0.06	0.12×0.08×0.03	0.15×0.12×0.1
Radiation	CuKα (λ = 1.54184Å)			
2Θ range/°	8.712 to 155.574	10.708 to 154.896	8.244 to 156.32	9.458 to 155.358
Index ranges	$-22 \le h \le 22$, $-13 \le k \le 15$, $-8 \le l \le 9$	$-9 \le h \le 10$, $-15 \le k \le 15$, $-8 \le l \le 10$	$-10 \le h \le 10$, $-11 \le k \le 12$, $-13 \le l \le 13$	$-10 \le h \le 10$, $-11 \le k \le 12$, $-23 \le l \le 23$
Reflections collected	17049	6361	13040	15002
Independent reflections	3395 [R_{int} = 5.45%, R_{sigma} = 7.58 %]	2812 [R_{int} = 5.71%, R_{sigma} = 5.9 %]	3517 [R_{int} = 6.81%, R_{sigma} = 7.14 %]	3095 [R_{int} = 4.61%, R_{sigma} = 7.69 %]
Data/restraints/parameters	3395/-/232	2812/-/231	3517/-/246	3095/-/219
g_1, g_2 in w^a	0.0855, 0	0.1140, 0	0.0001, 7.1347	0.0822, 0.3951
Goodness–of–fit on F^2, S^b	1.086	1.113	1.099	1.078
Final R and wR^c values [$I \ge 2\sigma(I)$]	R_1 = 4.22%, wR_2 = 11.35%	R_1 = 5.51%, wR_2 = 14.78%	R_1 = 5.5%, wR_2 = 13.71%	R_1 = 4.1%, wR_2 = 11.38%
Final R and wR^c values [all data]	R_1 = 4.77%, wR_2 = 12.32%	R_1 = 5.64%, wR_2 = 15.22%	R_1 = 5.89%, wR_2 = 13.88%	R_1 = 4.18%, wR_2 = 11.52%
Largest diff. peak/hole / e Å^{-3}	1.227/–1.461	1.821/–1.786	1.413/–1.063	1.384/–1.229
Flack parameter	/	0.00(2)	/	/

$^a w = 1/[\sigma F_o^2 + (g_1P)^2 + g_2P]$ where $P = (F_o^2 + 2F_c^2)/3$
$^b S = \{\Sigma[w(F_o^2 - F_c^2)2]/(N_r - N_p)\}^{1/2}$ where N_r = number of independent reflections, N_p = number of refined parameters.
$^c R = \Sigma||F_o| - |F_c|/\Sigma|F_o|$; $wR = \{\Sigma[w(F_o^2 - F_c^2)2]/\Sigma[w(F_o^2)2]\}^{1/2}$

Table S6. Selected bond lengths in the crystal structures of **1a, 2a, 3a** and **3.**

**1a**					
Atoms	Bond length/Å	Atoms	Bond length/Å	Atoms	Bond length/Å
C4–C5	1.373(5)	Mo1–O1	1.916(2)	N3–C6	1.334(5)
C6–C7	1.392(5)	Mo1–O2	2.038(2)	O1–C9	1.351(4)
C8–C9	1.421(4)	Mo1–O4	1.704(2)	O2–C2	1.323(3)
C8–C13	1.392(4)	Mo1–O5	1.706(2)	O3–C12	1.364(4)
C9–C10	1.392(5)	Mo1–O6	2.313(2)	O3–C14	1.425(4)
C10–C11	1.384(5)	N1–C1	1.281(4)	O6–C15	1.419(4)
C11–C12	1.399(4)	N1–N2	1.398(4)	O6–H6	0.88(3)
C12–C13	1.385(4)	N2–C2	1.289(4)		
Mo1–N1	2.244(2)	N3–C5	1.335(5)		
**2a**					
Atoms	Bond length/Å	Atoms	Bond length/Å	Atoms	Bond length/Å
C5–C6	1.381(14)	Mo1–O1	2.024(6)	N3–C5	1.327(14)
C6–C7	1.395(14)	Mo1–O2	1.918(5)	O1–C2	1.308(12)
C8–C9	1.402(13)	Mo1–O4	1.707(7)	O2–C9	1.349(12)
C8–C13	1.408(12)	Mo1–O5	1.704(9)	O3–C12	1.371(12)
C9–C10	1.399(12)	Mo1–O6	2.295(7)	O3–C14	1.432(12)
C10–C11	1.404(13)	N1–C1	1.286(12)	O6–C15	1.413(13)
C11–C12	1.405(14)	N1–N2	1.409(10)	O6–H6	0.88(11)
C12–C13	1.391(12)	N2–C2	1.287(11)		
Mo1–N1	2.226(8)	N3–C4	1.343(12)		
**3a**					
Atoms	Bond length/Å	Atoms	Bond length/Å	Atoms	Bond length/Å
C5–C6	1.376(9)	Mo1–N1	2.243(4)	N3–H3B	0.88(5)
C6–C7	1.396(9)	Mo1–O1	1.919(4)	O1–C10	1.355(7)
C7–C8	1.386(9)	Mo1–O2	2.001(4)	O2–C2	1.338(6)
C9–C10	1.404(8)	Mo1–O4	1.700(5)	O3–C13	1.369(8)
C9–C14	1.414(8)	Mo1–O5	1.711(4)	O3–C15	1.422(8)
C10–C11	1.392(8)	Mo1–O6	2.287(4)	O6–C00O	1.425(9)
C11–C12	1.379(9)	N1–N2	1.389(6)	O6–H6	0.872(16)
C12–C13	1.383(8)	N3–H3A	0.85(4)		
**3**					
Atoms	Bond length/Å	Atoms	Bond length/Å	Atoms	Bond length/Å
C3–C4	1.410(4)	C11–C12	1.385(4)	N1–C2	1.291(3)
C3–C8	1.395(4)	C12–C13	1.386(4)	N1–N2	1.391(3)
C4–C5	1.389(4)	C13–C14	1.383(4)	O1–C4	1.351(4)
C5–C6	1.390(4)	Mo1–N1	2.238(2)	O2–C1	1.329(3)
C6–C7	1.396(4)	Mo1–N3_a	2.536(2)	O3–C7	1.364(3)
C7–C8	1.388(4)	Mo1–O1	1.912(2)	O3–C15	1.411(4)
C9–C10	1.409(4)	Mo1–O2	2.0197(18)		
C9–C14	1.402(4)	Mo1–O4	1.705(3)		
C10–C11	1.393(4)	Mo1–O5	1.714(2)		

Table S7. Selected bond angles in the crystal structure of **1a, 2a, 3a** and **3.**

**1a**					
Atoms	Bond angle/°	Atoms	Bond angle/°	Atoms	Bond angle/°
N1–C1–C8	123.6(2)	O1–C9–C10	118.6(2)	O4–Mo1–O6	168.65(10)
N2–C2–C3	117.3(3)	C9–C10–C11	121.2(3)	O5–Mo1–N1	160.79(11)
O2–C2–C3	117.5(3)	C10–C11–C12	120.2(3)	O5–Mo1–O6	85.17(10)
O2–C2–N2	125.3(3)	O3–C12–C11	125.3(3)	O6–Mo1–N1	77.73(9)
C2–C3–C4	119.7(3)	O3–C12–C13	115.4(3)	Mo1–N1–C1	128.5(2)
C2–C3–C7	122.0(3)	O1–Mo1–N1	81.64(9)	Mo1–N1–N2	115.35(17)
C4–C3–C7	118.3(3)	O1–Mo1–O2	149.36(9)	N2–N1–C1	115.8(2)
C3–C4–C5	118.7(3)	O1–Mo1–O4	98.87(11)	N1–N2–C2	109.2(2)
N3–C5–C4	123.8(4)	O1–Mo1–O5	104.62(10)	C5–N3–C6	117.6(3)
N3–C6–C7	122.9(3)	O1–Mo1–O6	81.58(9)	Mo1–O1–C9	134.36(19)
C3–C7–C6	118.8(3)	O2–Mo1–N1	72.05(9)	Mo1–O2–C2	117.62(19)
C1–C8–C9	122.7(3)	O2–Mo1–O4	97.09(10)	C12–O3–C14	117.9(2)
C1–C8–C13	117.9(3)	O2–Mo1–O5	95.99(10)	C15–O6–H6	107.6(15)
C9–C8–C13	119.4(3)	O2–Mo1–O6	77.78(8)	Mo1–O6–C15	127.8(2)
C8–C9–C10	118.6(3)	O4–Mo1–N1	91.09(10)	Mo1–O6–H6	118.4(15)
O1–C9–C8	122.8(3)	O4–Mo1–O5	105.57(11)		
**2a**					
Atoms	Bond angle/°	Atoms	Bond angle/°	Atoms	Bond angle/°
N1–C1–C8	123.2(9)	O2–C9–C10	116.9(9)	O4–Mo1–O6	169.1(3)
N2–C2–C3	118.2(9)	C9–C10–C11	120.1(8)	O5–Mo1–N1	160.8(3)
O1–C2–C3	116.9(7)	C10–C11–C12	119.6(8)	O5–Mo1–O6	85.3(3)
O1–C2–N2	124.9(8)	O3–C12–C11	124.3(8)	O6–Mo1–N1	77.6(3)
C2–C3–C4	118.9(9)	O3–C12–C13	115.0(9)	Mo1–N1–C1	128.3(6)
C2–C3–C7	123.3(8)	O1–Mo1–N1	71.9(3)	Mo1–N1–N2	115.4(5)
C4–C3–C7	117.8(8)	O1–Mo1–O2	149.4(3)	N2–N1–C1	115.8(7)
N3–C4–C3	122.2(9)	O1–Mo1–O4	96.6(3)	N1–N2–C2	108.7(7)
N3–C5–C6	123.3(8)	O1–Mo1–O5	96.8(3)	C4–N3–C5	118.7(8)
C5–C6–C7	118.6(10)	O1–Mo1–O6	80.0(3)	Mo1–O1–C2	118.6(5)
C3–C7–C6	119.4(9)	O2–Mo1–N1	81.8(4)	Mo1–O2–C9	134.1(6)
C1–C8–C9	122.8(8)	O2–Mo1–O4	99.5(3)	C12–O3–C14	117.4(9)
C1–C8–C13	116.9(8)	O2–Mo1–O5	103.6(4)	C15–O6–H6	110(2)
C9–C8–C13	120.3(8)	O2–Mo1–O6	79.2(2)	Mo1–O6–C15	129.4(7)
C8–C9–C10	119.9(8)	O4–Mo1–N1	91.5(3)	Mo1–O6–H6	121(2)
O2–C9–C8	123.1(8)	O4–Mo1–O5	105.5(4)		
**3a**					
Atoms	Bond angle/°	Atoms	Bond angle/°	Atoms	Bond angle/°
N1–C1–C9	123.5(5)	O1–C10–C9	122.9(5)	O4–Mo1–O6	170.5(2)
N2–C2–C3	123.1(5)	O1–C10–C11	117.8(5)	O5–Mo1–N1	159.37(19)
O2–C2–C3	114.7(5)	C10–C11–C12	120.7(5)	O5–Mo1–O6	83.73(18)
O2–C2–N2	122.2(5)	C11–C12–C13	121.0(6)	O6–Mo1–N1	77.55(17)
C2–C3–C4	123.2(5)	C12–C13–C14	119.6(6)	Mo1–N1–C1	127.9(4)
C2–C3–C8	118.4(5)	O3–C13–C12	115.7(5)	Mo1–N1–N2	115.5(3)
C4–C3–C8	118.4(5)	O3–C13–C14	124.7(6)	N2–N1–C1	116.5(4)
C3–C4–C5	118.7(6)	O1–Mo1–N1	81.21(16)	N1–N2–C2	110.2(4)

N3–C4–C3	121.9(5)	O1–Mo1–O2	147.99(17)	C4–N3–H3B	116(5)
N3–C4–C5	119.4(5)	O1–Mo1–O4	99.4(2)	C4–N3–H3A	119(6)
C4–C5–C6	121.8(6)	O1–Mo1–O5	103.99(19)	H3A–N3–H3B	97(8)
C5–C6–C7	120.4(6)	O1–Mo1–O6	79.55(16)	Mo1–O1–C10	134.2(4)
C6–C7–C8	118.2(6)	O2–Mo1–N1	71.65(16)	Mo1–O2–C2	120.5(4)
C3–C8–C7	122.5(6)	O2–Mo1–O4	98.6(2)	C13–O3–C15	117.4(5)
C1–C9–C10	122.7(5)	O2–Mo1–O5	96.28(18)	C00O–O6–H6	109(2)
C1–C9–C14	117.6(5)	O2–Mo1–O6	78.32(17)	Mo1–O6–C00O	125.0(4)
C10–C9–C14	119.6(5)	O4–Mo1–N1	93.0(2)	Mo1–O6–H6	127(2)
C9–C10–C11	119.2(5)	O4–Mo1–O5	105.6(2)		
**3**					
Atoms	Bond angle/°	Atoms	Bond angle/°	Atoms	Bond angle/°
N2–C1–C9	120.5(3)	C1–C9–C14	118.1(3)	O2–Mo1–O5	96.85(9)
O2–C1–C9	117.0(2)	C10–C9–C14	118.9(3)	O4–Mo1–N1	94.60(9)
O2–C1–N2	122.4(2)	C9–C10–C11	118.9(3)	O4–Mo1–N3_a	172.18(8)
N1–C2–C3	123.9(3)	N3–C10–C9	122.6(2)	O4–Mo1–O5	106.45(10)
C2–C3–C4	122.6(2)	N3–C10–C11	118.3(2)	O5–Mo1–N1	156.83(10)
C2–C3–C8	117.1(3)	C10–C11–C12	121.2(3)	O5–Mo1–N3_a	79.87(9)
C4–C3–C8	120.2(3)	C11–C12–C13	120.4(3)	Mo1–N1–C2	129.44(18)
C3–C4–C5	118.9(3)	C12–C13–C14	119.0(3)	Mo1–N1–N2	115.41(15)
O1–C4–C3	122.5(3)	N1–Mo1–N3_a	78.43(8)	N2–N1–C2	115.0(2)
O1–C4–C5	118.6(3)	O1–Mo1–N1	81.32(8)	N1–N2–C1	110.0(2)
C4–C5–C6	120.5(3)	O1–Mo1–N3_a	82.28(8)	C10–N3–Mo1_a	118.94(18)
C5–C6–C7	120.6(3)	O1–Mo1–O2	149.92(8)	Mo1–O1–C4	139.10(18)
C6–C7–C8	119.2(3)	O1–Mo1–O4	100.31(10)	Mo1–O2–C1	119.55(15)
O3–C7–C6	124.6(3)	O1–Mo1–O5	103.74(9)	C7–O3–C15	117.8(2)
O3–C7–C8	116.2(3)	O2–Mo1–N1	71.45(8)		
C3–C8–C7	120.5(3)	O2–Mo1–N3_a	80.00(7)		
C1–C9–C10	123.0(2)	O2–Mo1–O4	94.49(9)		

Table S8. Hydrogen bond parameters in the crystal structure of **1a, 2a, 3a** and **3.**

**1a**					
D–H···A	D-H	H···A	D···A	∠D–H···A	Symmetry code
O6–H6···N3	0.88(3)	1.82(3)	2.677(4)	163(3)	–*x*, 1/2+*y*, 1/2–*z*
C1–H1···O4	0.95	2.37	3.307(4)	170	*x*, 1/2–*y*, 1/2+*z*
C6–H6A···O5	0.95	2.50	3.409(4)	159	–*x*, 1–*y*, –*z*
C10–H10···O3	0.95	2.41	3.319(3)	161	1–*x*, 1/2+*y*, 3/2–*z*
**2a**					
D–H···A	D-H	H···A	D···A	∠D–H···A	Symmetry code
O6–H6···N3	0.88(11)	1.82(11)	2.684(11)	168(9)	*x*, *y*, –1+*z*
C1–H1···O4	0.95	2.41	3.319(12)	160	1–*x*, 1/2+*y*, 1–*z*
C5–H5···O5	0.95	2.35	3.225(12)	153	–*x*, 1/2+*y*, 2–*z*
C11–H11···O4	0.95	2.55	3.303(11)	136	*x*, *y*, –1+*z*
C14–H14C···O5	0.98	2.55	3.358(12)	140	1–*x*, 1/2+*y*, –*z*
**3a**					
D–H···A	D-H	H···A	D···A	∠D–H···A	Symmetry code
O6–H6···N3	0.88(3)	1.82(3)	2.677(4)	163(3)	–*x*, 1/2+*y*, 1/2–*z*
C1–H1···O4	0.95	2.37	3.307(4)	170	*x*, 1/2–*y*, 1/2+*z*
C6–H6A···O5	0.95	2.50	3.409(4)	159	–*x*, 1–*y*, –*z*
C10–H10···O3	0.95	2.41	3.319(3)	161	1–*x*, 1/2+*y*, 3/2–*z*
**3**					
D–H···A	D-H	H···A	D···A	∠D–H···A	Symmetry code
N3–H3A···O5	0.91	2.22	3.129(3)	177	*x*, 1+*y*, *z*
C11–H11···O3	0.95	2.55	3.307(4)	137	–1+*x*, 3/2–*y*, –1/2+*z*
C15–H15B···O4	0.98	2.56	3.363(4)	139	*x*, 1/2–*y*, 1/2+*z*

IR-ATR spectra

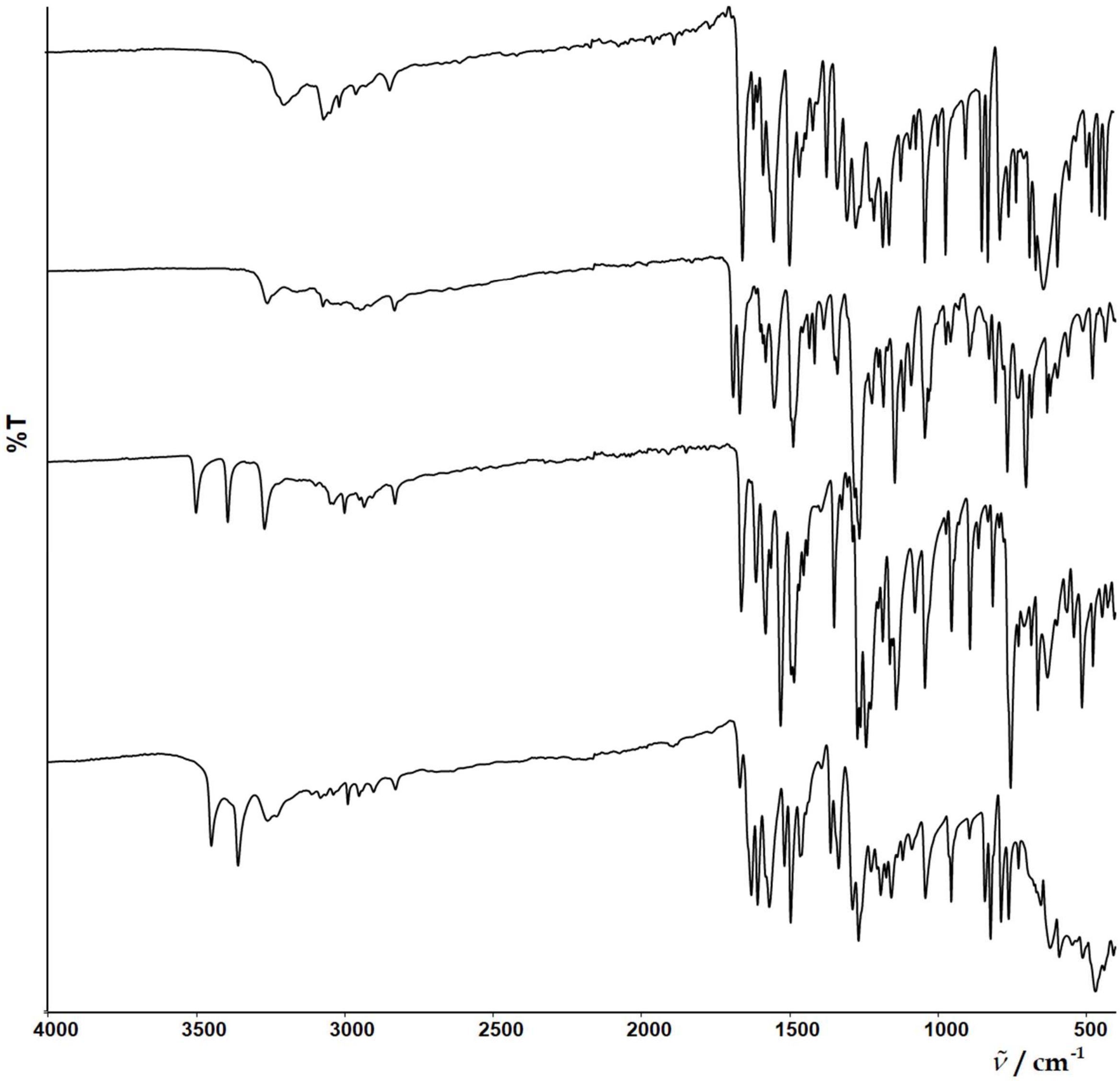

Figure S7. IR-ATR spectra of hydrazones (H_2L^1- H_2L^4, from top to bottom).

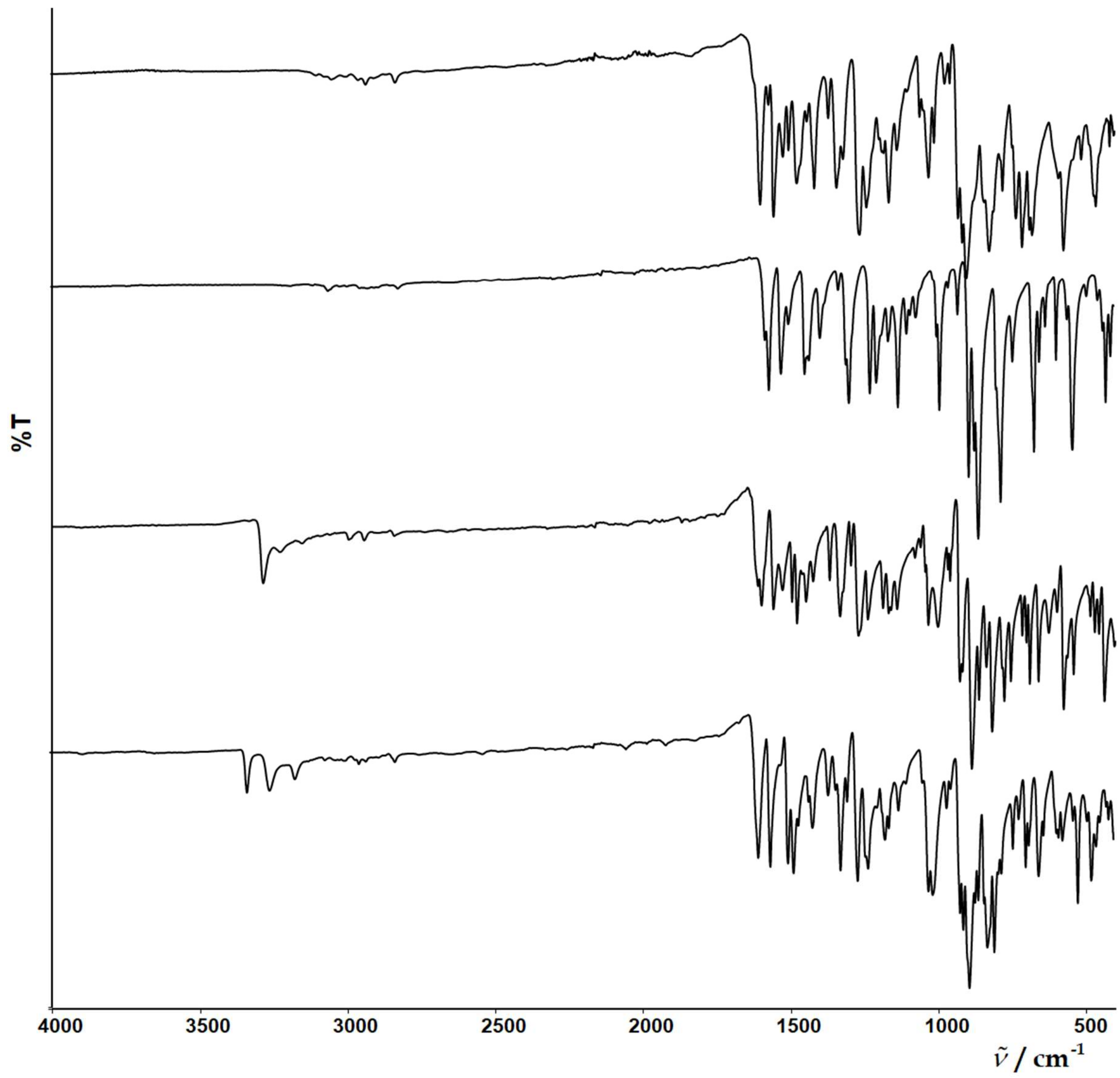

Figure S8. IR-ATR spectra of Mo(VI) metallosupramolecular complexes (**1-4**, from top to bottom).

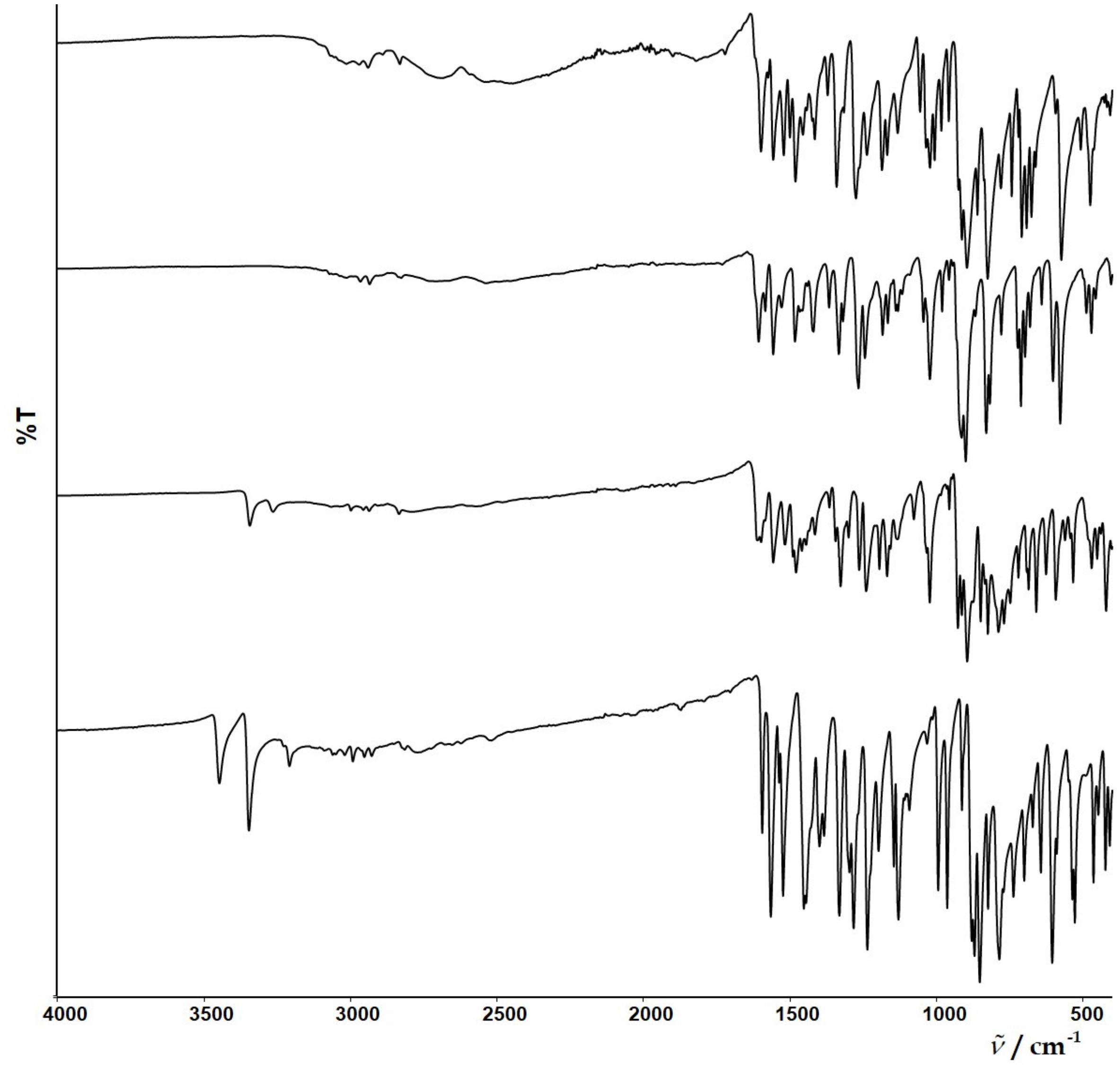

Figure S9. IR-ATR spectra of the mononuclear Mo(VI) complexes (**1a**-**4a**, from top to bottom).

NMR spectroscopy

Table S9 ^{1}H and ^{13}C chemical shifts (ppm) of $\mathbf{H_2L^1}$ and **1**.

Atom	$\mathbf{H_2L^1}$		**1**	
	δ / ppm (^{1}H)	δ / ppm (^{13}C)	δ / ppm (^{1}H)	δ / ppm (^{13}C)
**1**	8.75	148.07	8.98	156.73
**4**	–	160.94	–	166.63
**5**	–	139.53	–	136.90
**6**	7.92	121.06	7.88	120.94
**7**	8.85	149.83	8.77	149.96
**8**	–	–	–	–
**9**	8.85	149.83	8.77	149.96
**10**	7.92	121.98	7.88	120.94
**11**	–	118.41	–	119.57
**12**	–	151.17	–	153.36
**13**	6.94	116.87	6.93	118.78
**14**	6.97	118.15	7.18	121.54
**15**	–	151.71	–	153.02
**16**	7.22	111.50	7.39	116.51
**OH**	10.66	–	–	–
**NH**	12.37	–	–	–
**OMe**	3.77	54.94	3.78	55.14

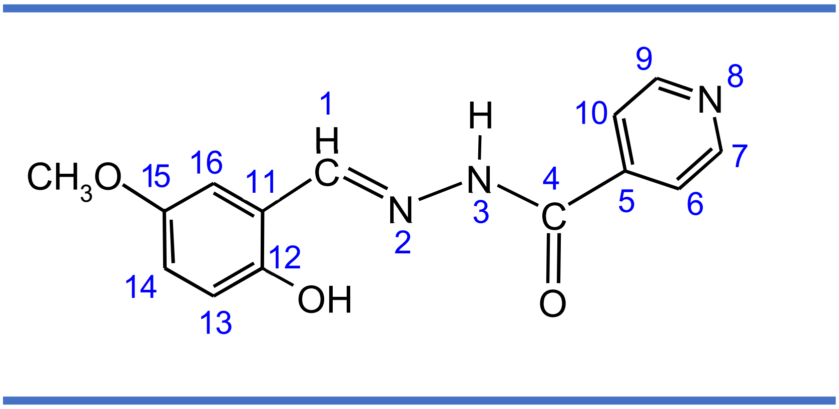

Scheme S2. The structural formula of $\mathbf{H_2L^1}$ with the NMR numbering scheme

Table S10 ^{1}H and ^{13}C chemical shifts (ppm) of $\mathbf{H_2L^2}$ and **2**.

Atom	$\mathbf{H_2L^2}$		**2**	
	δ / ppm (^{1}H)	δ / ppm (^{13}C)	δ / ppm (^{1}H)	δ / ppm (^{13}C)
**1**	8.73	147.66	8.95	156.94
**4**	–	161.06	–	167.80
**5**	–	128.25	–	126.64
**6**	9.19	148.21	9.15	149.24
**7**	–	–	–	–
**8**	8.83	151.90	8.76	152.86
**9**	7.61	123.07	7.57	124.44
**10**	8.36	134.98	8.31	135.90
**11**	–	118.40	–	120.72
**12**	–	151.70	–	154.30
**13**	6.94	116.84	6.92	119.79
**14**	6.97	117.98	7.16	122.31
**15**	–	151.13	–	154.08
**16**	7.21	111.66	7.38	117.48
**OH**	10.72	–	–	–
**NH**	12.33	–	–	–
**OMe**	3.77	54.94	3.78	56.20

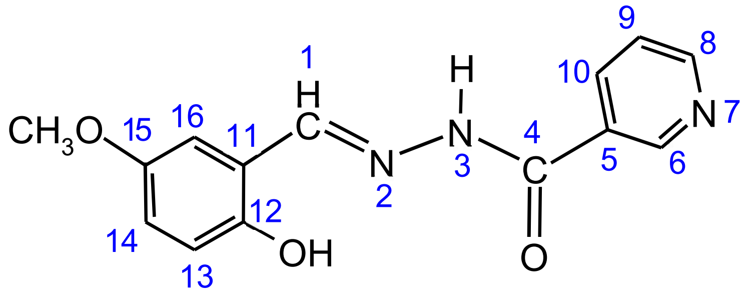

Scheme S3. The structural formula of $\mathbf{H_2L^2}$ with the NMR numbering scheme

Table S11 ^{1}H and ^{13}C chemical shifts (ppm) of **H_2L^3** and **3**.

Atom	H_2L^3		3	
	δ / ppm (^{1}H)	δ / ppm (^{13}C)	δ / ppm (^{1}H)	δ / ppm (^{13}C)
**1**	8.63	146.73	8.93	154.04
**4**	–	164.59	–	169.14
**5**	–	112.09	–	108.85
**6**	–	149.79	–	148.89
**7**	6.84	116.03	6.79	115.40
**8**	7.25	132.06	7.20	132.06
**9**	6.63	114.17	6.57	114.31
**10**	7.67	127.81	7.69	129.38
**11**	–	118.37	–	119.83
**12**	–	151.04	–	153.06
**13**	6.92	116.73	6.90	118.67
**14**	6.92	117.37	7.13	120.66
**15**	–	151.59	–	152.83
**16**	7.12	112.23	7.27	115.91
**OH**	11.02	–	–	–
**NH**	11.96	–	–	–
**OMe**	3.74	54.90	3.77	55.05
**NH2**	6.54	–	7.00	–

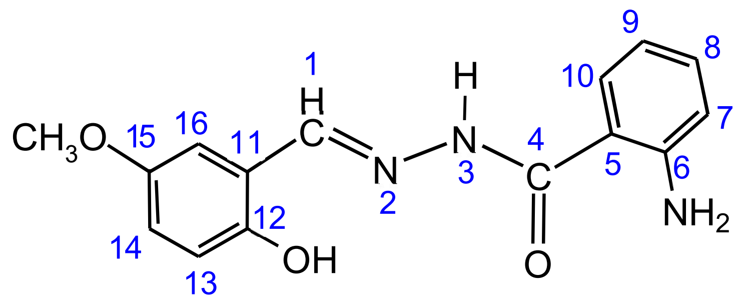

Scheme S4. The structural formula of **H_2L^3** with the NMR numbering scheme

Table S12 ^{1}H and ^{13}C chemical shifts (ppm) of **H_2L^4** and **4**.

Atom	H_2L^4		4	
	δ / ppm (^{1}H)	δ / ppm (^{13}C)	δ / ppm (^{1}H)	δ / ppm (^{13}C)
**1**	8.64	145.95	8.73	152.14
**4**	–	162.45	–	169.04
**5**	–	118.33	–	115.14
**6**	6.71	112.25	6.60	112.49
**7**	7.80	128.98	7.68	129.30
**8**	–	152.03	–	152.09
**9**	7.80	128.98	7.68	129.30
**10**	6.71	112.25	6.60	112.49
**11**	–	118.51	–	120.13
**12**	–	150.91	–	152.87
**13**	6.91	116.69	6.86	118.47
**14**	6.91	117.18	7.08	120.02
**15**		151.57	–	152.79
**16**	7.12	112.10	7.28	115.79
**OH**	11.03	–	–	–
**NH**	11.88	–	–	–
**OMe**	3.73	54.89	3.76	55.08
**NH2**	5.89	–	5.93	–

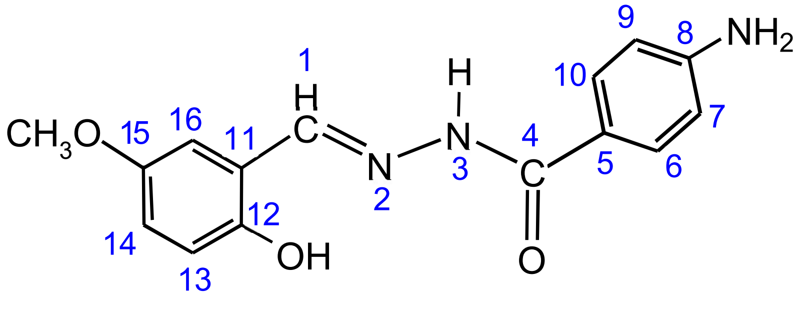

Scheme S5. The structural formula of **H_2L^4** with the NMR numbering scheme

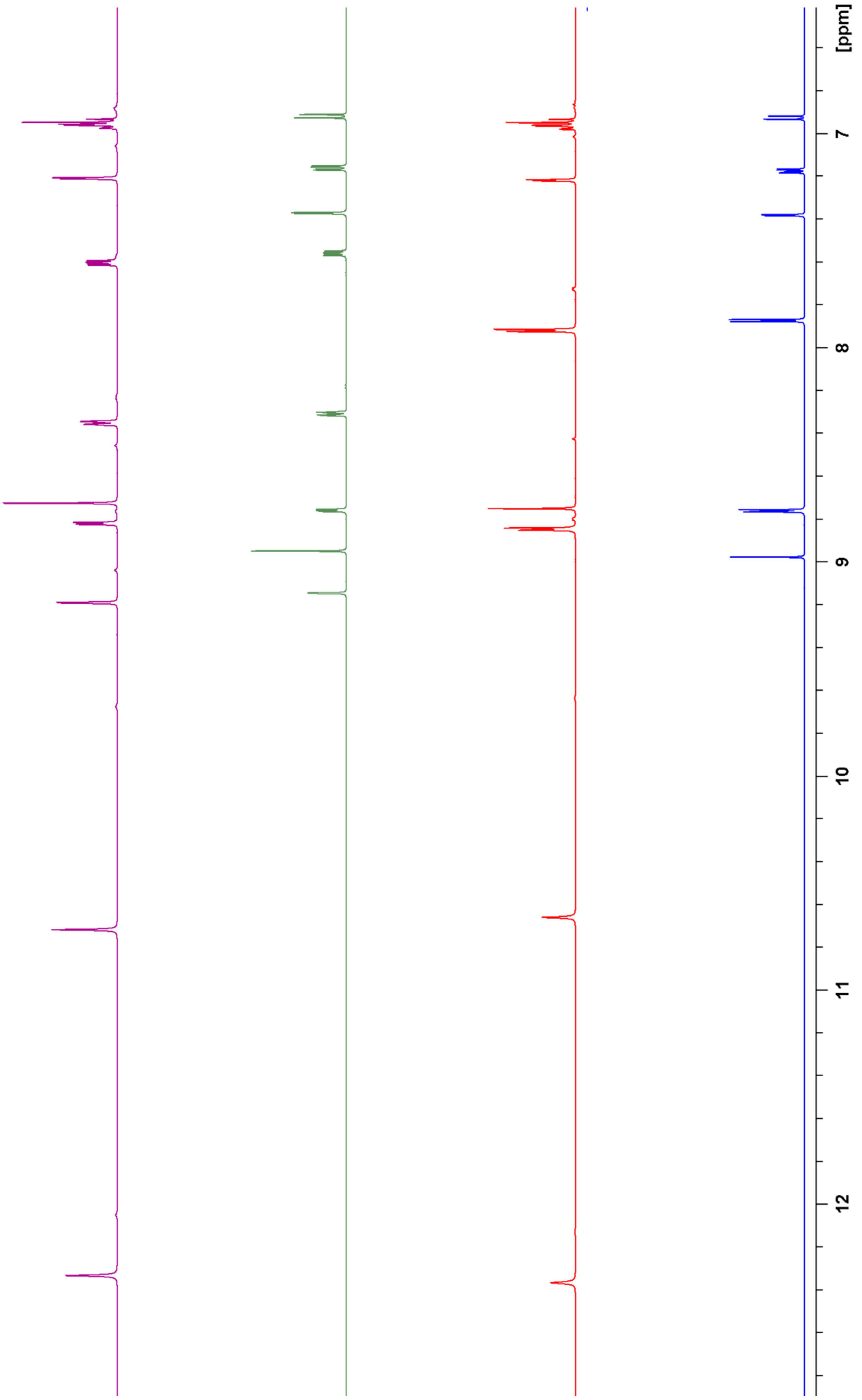

Figure S10. A portion of the ^{1}H NMR spectra in dmso-d_6 of: $[MoO_2(L^1)]_n$ (**1**) - blue line; H_2L^1 - red line, $[MoO_2(L^1)]_n$ (**2**) – green line , $H_2L^{\mathbf{2}}$ – purple line

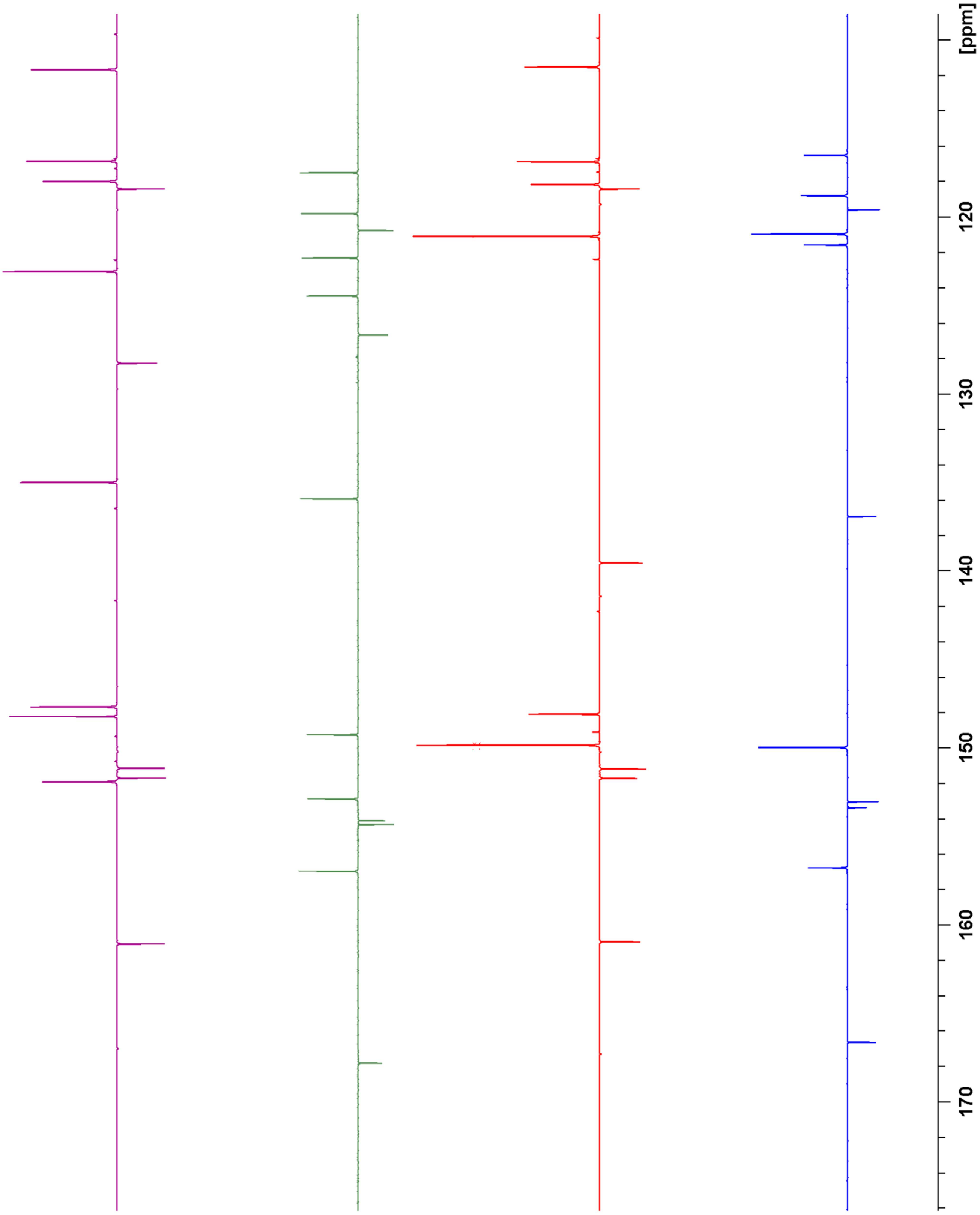

Figure S11. A portion of the ^{13}C NMR spectra in dmso-d_6 of: $[MoO_2(L^1)]_n$ (**1**) - blue line; H_2L^1 - red line, $[MoO_2(L^1)]_n$ (**2**) – green line , H_2L^2 – purple line

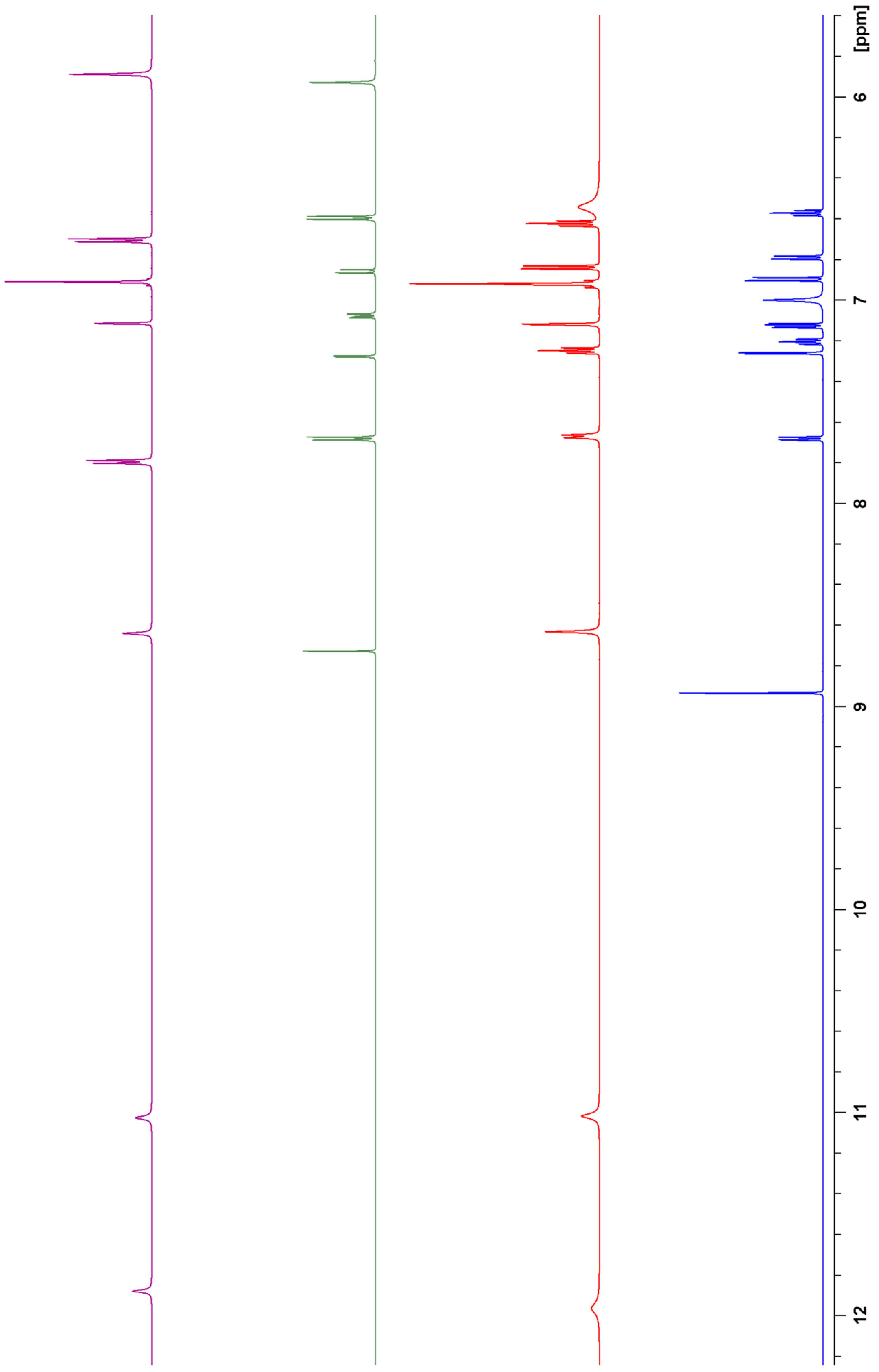

Figure S12. A portion of the ^{1}H NMR spectra in dmso-d_6 of: $[MoO_2(L^3)]_2$ (**3**) - blue line; H_2L^3 - red line, $[MoO_2(L^4)]_2$ (**4**) – green line, $H_2L^{\mathbf{4}}$ – purple line

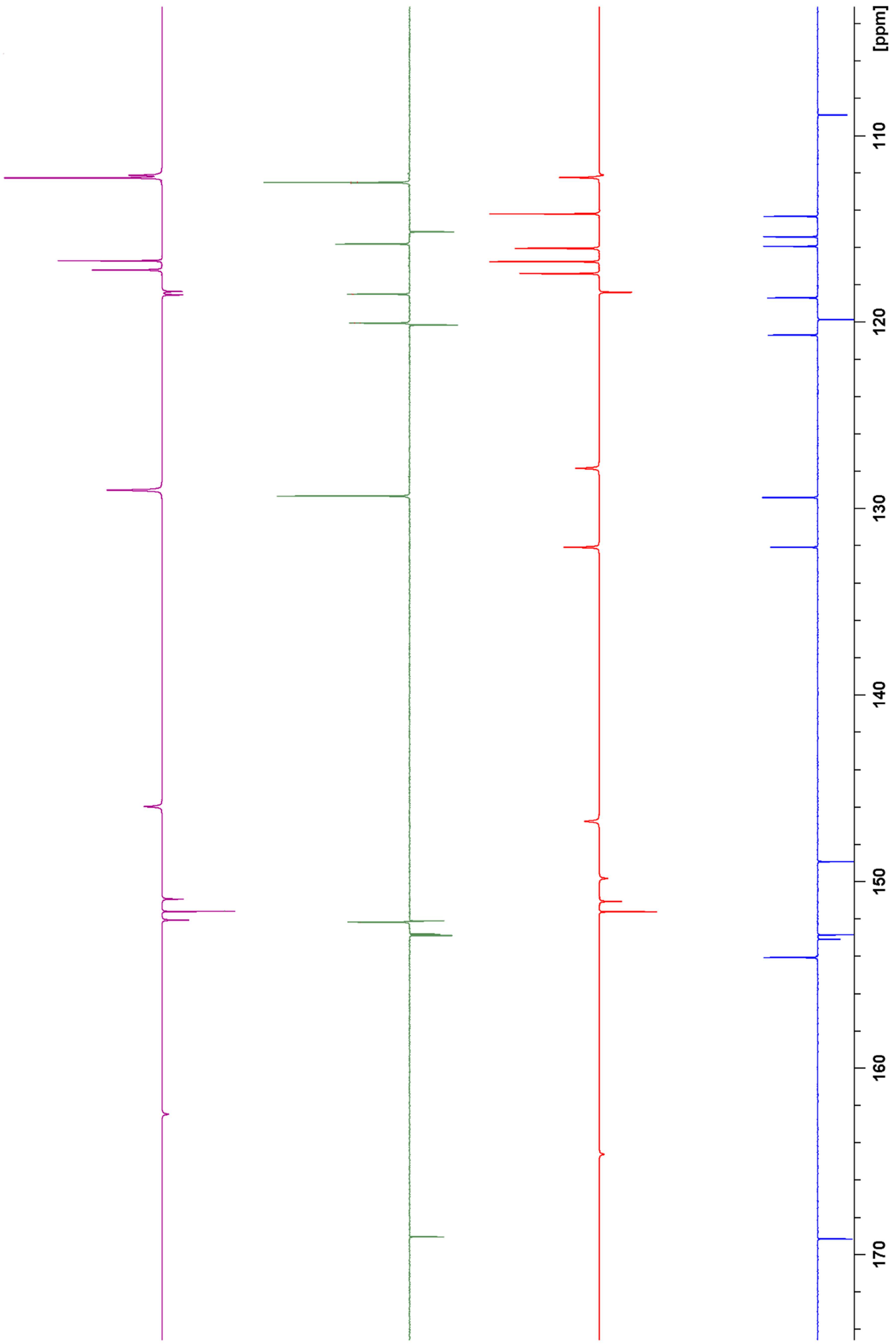

Figure S13. A portion of the ^{13}C NMR spectra in dmso-d_6 of: $[MoO_2(L^3)]_2$ (**3**) - blue line; H_2L^3 - red line, $[MoO_2(L^4)]_2$ (**4**) – green line, $H_2L^{\mathbf{4}}$ – purple line

Table S13. Imaginary frequency (cm^{-1}) of the calculated transition states shown in Table S14.

	L1	L2	L3	L4
TS	471i	470i	473i	476i

Table S14. DFT Coordinates of all species.

A - DFT coordinates of organic molecules

A0-MeOH

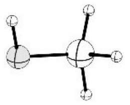

8	-0.748606000	-0.122482000	0.000001000
6	0.661940000	0.019648000	0.000035000
1	1.036807000	0.543842000	0.893075000
1	1.036822000	0.543722000	-0.893232000
1	1.079608000	-0.990888000	-0.000013000
1	-1.136031000	0.765290000	-0.000051000

A1 – C_2H_4

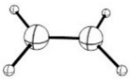

6	0.000000000	0.665616000	0.000000000
1	0.923789000	1.239603000	0.000000000
1	-0.923751000	1.239647000	0.000000000
6	0.000000000	-0.665616000	0.000000000
1	-0.923789000	-1.239603000	0.000000000
1	0.923751000	-1.239647000	0.000000000

A2– epo

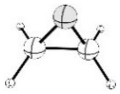

6	0.000000000	0.823033000	0.000000000
6	0.663277000	-0.487998000	0.000000000
1	-0.044284000	1.406100000	0.920513000
1	1.105429000	-0.869573000	0.920803000
8	-0.762744000	-0.385408000	0.000000000
1	-0.044284000	1.406100000	-0.920513000
1	1.105429000	-0.869573000	-0.920803000

A3 – TBHP

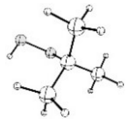

6	0.386063000	0.000211000	0.035516000
8	-0.724877000	-0.051131000	-0.892876000
8	-1.972121000	0.107335000	-0.155683000
6	1.585594000	-0.148364000	-0.908710000
6	0.308477000	-1.169459000	1.023961000
6	0.408392000	1.352799000	0.755437000
1	-2.332288000	-0.792908000	-0.245178000
1	1.280879000	1.423828000	1.414956000
1	1.184972000	-1.179544000	1.681110000
1	2.518340000	-0.109351000	-0.336278000
1	-0.583795000	-1.085739000	1.651566000
1	0.273436000	-2.124477000	0.486863000
1	1.545636000	-1.103632000	-1.442442000
1	1.596327000	0.660559000	-1.645981000
1	0.455220000	2.169404000	0.027401000
1	-0.493901000	1.481099000	1.359235000

A4 – tBuOH

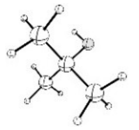

6	-0.005589000	-0.000026000	0.014420000
8	0.015118000	-0.000431000	1.451985000
1	0.945639000	0.001902000	1.728630000
6	-1.490947000	-0.005338000	-0.356041000
6	0.694937000	-1.262772000	-0.510972000
6	0.685524000	1.268259000	-0.510112000
1	0.646742000	1.325318000	-1.604317000
1	0.656167000	-1.319650000	-1.605180000
1	-1.623187000	-0.004815000	-1.443350000
1	1.752964000	-1.271531000	-0.216357000
1	0.219546000	-2.158915000	-0.098630000
1	-1.981082000	-0.894002000	0.055230000
1	-1.987701000	0.878867000	0.056897000
1	0.203120000	2.160551000	-0.097564000
1	1.743307000	1.284985000	-0.215007000

B- DFT coordinates for TBHP as oxidant – Gas phase

B1 - With Ligand L[1]

MoO_2L^1(MeOH)

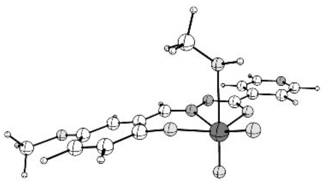

42	0.287666000	-1.514676000	-0.504120000
8	2.144014000	-0.752365000	-0.294954000
8	-1.565353000	-1.411090000	0.082883000
8	0.645363000	-1.424014000	2.021257000
1	1.022133000	-2.319978000	2.057454000
8	-5.624978000	2.342528000	-0.094947000
8	0.108712000	-1.406015000	-2.185325000
8	0.703090000	-3.125229000	-0.109334000
7	0.142879000	0.712702000	-0.023676000
7	6.385133000	1.929815000	0.010732000
7	1.345001000	1.380290000	0.076735000
6	3.724151000	1.024081000	-0.060427000
6	-2.272172000	0.906463000	0.014416000
6	-2.539511000	-0.488793000	-0.007227000
6	-4.921762000	-0.010062000	-0.100932000
1	-5.937399000	-0.385934000	-0.149143000
6	-4.666595000	1.371263000	-0.070131000
6	2.328295000	0.539090000	-0.096256000
6	-3.867804000	-0.921211000	-0.066366000
1	-4.060855000	-1.988940000	-0.081053000
6	4.790253000	0.138240000	-0.253309000
1	4.603904000	-0.914183000	-0.434553000
6	-3.346895000	1.813422000	-0.007801000
1	-3.157402000	2.882601000	0.012215000
6	4.018770000	2.375365000	0.167166000
1	3.219202000	3.091178000	0.319357000
6	-0.931308000	1.434271000	0.079755000
1	-0.800238000	2.509051000	0.212184000
6	6.090244000	0.642697000	-0.208336000
1	6.938353000	-0.023687000	-0.356031000
6	5.354420000	2.768014000	0.193007000
1	5.612969000	3.810804000	0.368842000
6	-6.984409000	1.943800000	-0.175304000
1	-7.184752000	1.374025000	-1.092380000
1	-7.565691000	2.867247000	-0.191324000
1	-7.282326000	1.344269000	0.695383000
6	-0.414593000	-1.326707000	2.981485000
1	-1.206470000	-2.056779000	2.782677000
1	-0.825328000	-0.320143000	2.892063000
1	-0.022459000	-1.462911000	3.995748000

MoO_2L^1

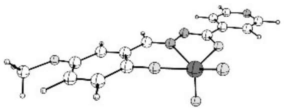

42	0.300712000	1.662152000	0.087558000
8	2.131126000	0.868342000	-0.157018000
8	-1.543841000	1.455504000	-0.481142000
8	-5.630245000	-2.209257000	0.156519000
8	0.225834000	1.990496000	1.749401000
8	0.669789000	3.074243000	-0.778435000
7	0.145197000	-0.600811000	-0.069358000
7	6.392482000	-1.800863000	-0.007601000
7	1.342419000	-1.288922000	-0.067994000
6	3.727298000	-0.912974000	-0.059047000
6	-2.270159000	-0.815108000	-0.092462000
6	-2.530092000	0.566999000	-0.284022000
6	-4.911996000	0.114421000	-0.177960000
1	-5.926944000	0.493371000	-0.212643000
6	-4.664984000	-1.258787000	0.006929000
6	2.330083000	-0.438522000	-0.086999000
6	-3.855864000	1.009770000	-0.324720000
1	-4.042961000	2.067151000	-0.479305000
6	4.788157000	-0.000879000	-0.095367000
1	4.596485000	1.064921000	-0.144699000
6	-3.348662000	-1.710094000	0.042008000
1	-3.163758000	-2.770657000	0.183887000
6	4.028122000	-2.280661000	0.003791000
1	3.232849000	-3.016664000	0.033977000
6	-0.932089000	-1.334077000	-0.063664000
1	-0.790437000	-2.414537000	-0.028710000
6	6.091498000	-0.498081000	-0.066703000
1	6.936485000	0.187537000	-0.093181000
6	5.366646000	-2.663593000	0.026201000
1	5.631457000	-3.718319000	0.074453000
6	-6.990511000	-1.802689000	0.126139000
1	-7.220071000	-1.099047000	0.937103000
1	-7.575795000	-2.713181000	0.263920000
1	-7.253897000	-1.345764000	-0.836904000

MoO_2L^1(TBHP)

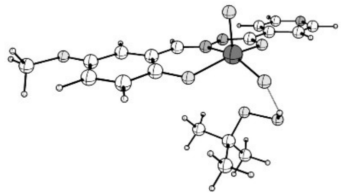

42	-0.200539000	0.318714000	1.597070000
8	-2.024500000	-0.201903000	0.966892000
8	1.608686000	0.708141000	1.024988000
8	5.803066000	-2.129558000	-1.192853000
8	0.041564000	-0.744004000	2.890367000
8	-0.669602000	1.848953000	2.211773000
7	-0.013600000	-1.090092000	-0.170877000
7	-6.229456000	-2.188571000	-0.943908000
7	-1.197745000	-1.590814000	-0.669697000
6	-3.584013000	-1.467361000	-0.331038000
6	2.403221000	-1.113035000	-0.353952000
6	2.624092000	-0.012739000	0.515019000
6	5.019590000	-0.312918000	0.259861000
1	6.022983000	0.010651000	0.511591000
6	4.811631000	-1.400696000	-0.606453000
6	-2.197538000	-1.081243000	-0.005342000
6	3.935696000	0.370678000	0.806967000
1	4.091723000	1.217242000	1.467378000

6	-4.662057000	-0.870418000	0.332002000
1	-4.490959000	-0.114728000	1.089856000
6	3.507130000	-1.784758000	-0.909567000
1	3.353729000	-2.624875000	-1.580252000
6	-3.857347000	-2.433641000	-1.308963000
1	-3.047878000	-2.917216000	-1.843669000
6	1.077558000	-1.543009000	-0.714226000
1	0.966198000	-2.300338000	-1.490558000
6	-5.954430000	-1.266983000	-0.012965000
1	-6.812487000	-0.819200000	0.485038000
6	-5.187156000	-2.751683000	-1.571865000
1	-5.430702000	-3.498357000	-2.325648000
6	7.151290000	-1.782421000	-0.914035000
1	7.379881000	-1.878801000	0.155530000
1	7.762936000	-2.488655000	-1.477697000
1	7.381652000	-0.760722000	-1.243916000
1	-1.608235000	2.793260000	0.905892000
8	-1.969909000	2.861220000	-0.006449000
8	-0.978379000	2.057130000	-0.708141000
6	-0.224636000	2.926920000	-1.606441000
6	-1.194558000	3.604470000	-2.580376000
1	-1.771308000	2.852762000	-3.129558000
1	-1.892277000	4.251078000	-2.043607000
1	-0.639555000	4.213424000	-3.302930000
1	-0.085268000	4.592289000	-0.219613000
6	0.582107000	3.947216000	-0.797521000
6	0.689837000	1.944232000	-2.344124000
1	0.098899000	1.170252000	-2.844729000
1	1.268678000	2.481437000	-3.103008000
1	1.391556000	1.465065000	-1.655898000
1	1.174608000	4.581387000	-1.466535000
1	1.264143000	3.438893000	-0.107737000

TS

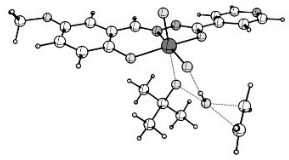

42	0.442669000	1.041296000	1.399288000
8	-1.297058000	0.222217000	0.762924000
8	2.262441000	1.562163000	0.968882000
8	0.823365000	-0.103332000	2.587051000
8	-0.265388000	2.377284000	2.246976000
7	-0.213692000	-1.109378000	-0.784762000
6	-2.606265000	-1.262673000	-0.573088000
6	4.608964000	1.336513000	1.037660000
1	4.643792000	2.243597000	1.632155000
6	4.492533000	-0.972672000	-0.510705000
1	4.462302000	-1.873645000	-1.116291000
7	0.882208000	-0.471353000	-0.246799000
6	3.358701000	0.854101000	0.639821000
8	6.821893000	-1.204704000	-0.505143000
6	3.296795000	-0.328428000	-0.148220000
6	-4.986441000	-1.447360000	-0.304802000
6	2.050737000	-0.887780000	-0.620818000
1	2.088540000	-1.719204000	-1.325891000
7	-5.104317000	-2.335884000	-1.298898000
6	-3.976699000	-2.692807000	-1.930935000
1	-4.090186000	-3.416930000	-2.735913000
6	-1.297445000	-0.691919000	-0.185850000
6	8.107435000	-0.751035000	-0.113122000

1	8.319942000	0.251977000	-0.507035000
1	8.217006000	-0.740544000	0.979589000
1	8.817666000	-1.462082000	-0.538671000
6	5.784395000	0.677881000	0.681299000
1	6.733272000	1.082213000	1.014725000
6	-3.775440000	-0.883048000	0.095606000
1	-3.732266000	-0.169194000	0.910130000
6	-2.715238000	-2.195964000	-1.612891000
1	-1.831916000	-2.517190000	-2.152629000
6	5.732862000	-0.487931000	-0.099831000
1	-1.040647000	3.265236000	1.071115000
8	-1.471127000	3.520047000	0.206803000
8	-0.114032000	2.395103000	-0.494736000
1	2.002054000	4.048337000	-0.298402000
6	1.226805000	4.363867000	-1.004057000
6	0.480970000	3.138795000	-1.557296000
6	-0.616658000	3.560768000	-2.550804000
1	-0.177164000	4.056566000	-3.424403000
1	-1.171912000	2.681278000	-2.894007000
1	-1.320022000	4.251900000	-2.079638000
1	0.529640000	5.024249000	-0.479242000
1	1.701931000	4.934702000	-1.811085000
6	1.464223000	2.201559000	-2.289119000
1	2.293474000	1.910033000	-1.642160000
1	0.948163000	1.300933000	-2.635751000
1	1.880153000	2.718628000	-3.162021000
1	-2.248779000	5.059022000	2.283639000
6	-2.671760000	4.974449000	1.287686000
1	-2.642405000	5.853075000	0.653307000
6	-3.172748000	3.775675000	0.824385000
1	-3.316919000	2.936361000	1.497123000
1	-3.710342000	3.725682000	-0.115929000
1	-5.910399000	-1.170967000	0.200777000

MoO(OR)(OH)L[1]

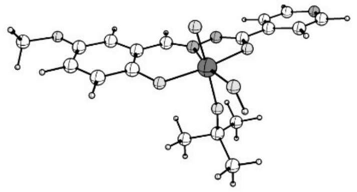

42	0.186435000	0.907250000	-1.131216000
8	2.016452000	0.090270000	-0.896490000
8	-1.662138000	1.091154000	-0.580431000
8	-5.779218000	-2.366780000	0.691713000
8	-0.240146000	-0.092879000	-2.437374000
8	0.731849000	2.373588000	-2.215740000
7	0.014876000	-0.913235000	0.187339000
7	6.192126000	-2.427989000	0.329053000
7	1.201857000	-1.534986000	0.515042000
6	3.565384000	-1.467964000	0.039680000
6	-2.408186000	-1.031837000	0.282622000
6	-2.655587000	0.226622000	-0.327243000
6	-5.040132000	-0.233480000	-0.274131000
1	-6.048430000	0.091682000	-0.503548000
6	-4.806801000	-1.476606000	0.336563000
6	2.188009000	-0.953915000	-0.114115000
6	-3.973898000	0.605252000	-0.594932000
1	-4.151223000	1.571066000	-1.056440000
6	4.628303000	-0.871621000	-0.647981000
1	4.452637000	-0.028853000	-1.306896000
6	-3.495608000	-1.859176000	0.612975000
1	-3.323602000	-2.824210000	1.080364000

6	3.845114000	-2.558015000	0.874868000
1	3.047144000	-3.046983000	1.421721000
6	-1.074942000	-1.498336000	0.578718000
1	-0.963810000	-2.425013000	1.142035000
6	5.910989000	-1.389807000	-0.467621000
1	6.756152000	-0.946336000	-0.991273000
6	5.164436000	-2.990608000	0.981289000
1	5.411477000	-3.834112000	1.623626000
6	-7.129120000	-2.034979000	0.408785000
1	-7.294291000	-1.898003000	-0.668238000
1	-7.724017000	-2.880172000	0.759187000
1	-7.443373000	-1.126979000	0.940969000
1	1.086908000	3.092968000	-1.663392000
8	0.596799000	1.983677000	0.432459000
6	0.667506000	2.571289000	1.718172000
6	1.787028000	1.868304000	2.504726000
1	1.571753000	0.799117000	2.601772000
1	2.745561000	1.980206000	1.987003000
1	1.883789000	2.295529000	3.509582000
1	1.946977000	4.185338000	1.009410000
6	0.991801000	4.064075000	1.533006000
6	-0.688612000	2.397955000	2.422652000
1	-0.924096000	1.336073000	2.547631000
1	-0.670123000	2.865762000	3.413921000
1	-1.487549000	2.858870000	1.833593000
1	1.066166000	4.572131000	2.501239000
1	0.206776000	4.555736000	0.947478000

B2 - With Ligand L[2]

MoO_2L^2(MeOH)

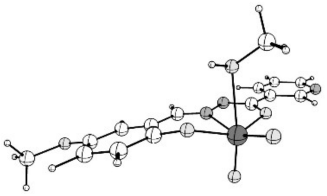

42	0.227751000	-1.433078000	-0.567858000
8	2.073895000	-0.662662000	-0.318689000
8	-1.591271000	-1.405056000	0.157720000
8	-0.053075000	-1.132358000	-2.212166000
8	0.479929000	-1.466194000	1.918391000
1	-0.406082000	-1.789601000	2.152923000
8	0.652908000	-3.076516000	-0.403993000
7	1.256893000	1.450529000	0.143995000
6	3.633354000	1.127048000	-0.056774000
6	-3.904607000	-0.957226000	0.030592000
1	-4.072336000	-2.028624000	-0.010928000
6	-3.446520000	1.783795000	0.159802000
1	-3.282333000	2.856316000	0.206703000
7	0.061775000	0.774329000	0.043272000
6	-2.586729000	-0.496055000	0.091742000
8	-5.736849000	2.262990000	0.103728000
6	-2.350167000	0.903958000	0.147546000
7	5.990865000	0.642239000	-0.272890000
6	-1.020859000	1.466334000	0.210944000
1	-0.915152000	2.536532000	0.394778000
6	6.241812000	1.934165000	-0.028667000
1	7.291627000	2.223447000	-0.023684000
6	5.245937000	2.886457000	0.209673000
1	5.511010000	3.922010000	0.400397000
6	2.246717000	0.628314000	-0.080816000
6	-7.086816000	1.835467000	0.018133000
1	-7.367120000	1.205717000	0.873289000
1	-7.278390000	1.285959000	-0.913172000
1	-7.689505000	2.745236000	0.029487000
6	-4.981085000	-0.071689000	0.028955000
1	-5.987930000	-0.470045000	-0.021797000
6	4.710765000	0.256689000	-0.285892000
1	4.520160000	-0.793499000	-0.489643000
6	1.473600000	-2.315568000	2.519210000
1	1.366720000	-3.349131000	2.176459000
1	1.405553000	-2.259946000	3.611151000
1	2.440001000	-1.929082000	2.195018000
6	3.918334000	2.477206000	0.195791000
1	3.104358000	3.171576000	0.374173000
6	-4.756598000	1.312954000	0.095484000

MoO_2L^2

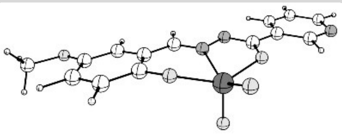

42	-0.305616000	-1.655195000	0.089458000
8	-2.133738000	-0.859062000	-0.155329000
8	1.538174000	-1.453051000	-0.483503000
8	-0.227376000	-1.979417000	1.752115000
8	-0.676160000	-3.069255000	-0.772685000
7	-1.341901000	1.297720000	-0.068623000
6	-3.724191000	0.924208000	-0.057964000
6	3.851473000	-1.013441000	-0.328394000
1	4.035379000	-2.071179000	-0.484508000
6	3.351444000	1.706642000	0.042341000
1	3.169617000	2.767562000	0.185720000
7	-0.146186000	0.608238000	-0.069657000
6	2.526950000	-0.567145000	-0.286183000
8	5.634626000	2.200171000	0.156283000
6	2.270458000	0.815100000	-0.092879000
7	-6.076144000	0.376414000	-0.081431000
6	0.933175000	1.337902000	-0.063715000
1	0.795226000	2.418855000	-0.028494000
6	-6.343911000	1.686308000	-0.016287000
1	-7.398449000	1.957115000	-0.001236000
6	-5.359803000	2.678904000	0.031781000
1	-5.639077000	3.726756000	0.084864000
6	-2.332564000	0.448533000	-0.085966000
6	6.993452000	1.789627000	0.125278000
1	7.255608000	1.333213000	-0.838392000
1	7.221019000	1.083937000	0.935075000
1	7.581709000	2.698050000	0.264430000
6	4.910395000	-0.121346000	-0.181109000
1	5.924301000	-0.502963000	-0.216961000
6	-4.790651000	0.011790000	-0.101728000
1	-4.586378000	-1.053995000	-0.155763000
6	-4.025681000	2.292670000	0.011234000
1	-3.220960000	3.019277000	0.047036000
6	4.666669000	1.251891000	0.005908000

MoO_2L^2(TBHP)

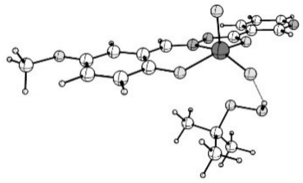

42	-0.207381000	0.305002000	1.593394000
8	-2.029337000	-0.212509000	0.958198000
8	1.602777000	0.700210000	1.027465000
8	0.034918000	-0.764556000	2.881248000
8	-0.678043000	1.831871000	2.215004000
7	-1.199943000	-1.588418000	-0.688520000
6	-3.583455000	-1.470806000	-0.348822000
6	3.930465000	0.364367000	0.813717000
1	4.084343000	1.207090000	1.479551000
6	3.506568000	-1.780738000	-0.916094000
1	3.355239000	-2.616987000	-1.592096000
7	-0.017278000	-1.091607000	-0.184282000
6	2.619719000	-0.017555000	0.515979000
8	5.803536000	-2.123932000	-1.195610000
6	2.400995000	-1.112560000	-0.359885000
7	-5.944158000	-1.186887000	0.073384000
6	1.075873000	-1.540323000	-0.726814000
1	0.967556000	-2.292620000	-1.508457000
6	-6.185415000	-2.102752000	-0.872531000
1	-7.233640000	-2.333498000	-1.056220000
6	-5.182695000	-2.747665000	-1.603483000
1	-5.440884000	-3.482511000	-2.359927000
6	-2.202681000	-1.085229000	-0.020866000

6	7.150613000	-1.778415000	-0.910465000
1	7.382077000	-0.754661000	-1.233276000
1	7.375953000	-1.881081000	0.159247000
1	7.764332000	-2.481107000	-1.476387000
6	5.016182000	-0.315706000	0.265696000
1	6.018827000	0.006524000	0.522063000
6	-4.667062000	-0.882296000	0.322876000
1	-4.483604000	-0.133531000	1.088220000
6	-3.857368000	-2.427682000	-1.337293000
1	-3.038713000	-2.897051000	-1.872283000
6	4.810278000	-1.398255000	-0.607301000
1	-1.617373000	2.780067000	0.910837000
8	-1.971204000	2.857565000	-0.003794000
8	-0.974284000	2.060823000	-0.705976000
1	1.267035000	3.430102000	-0.072621000
6	0.590490000	3.948486000	-0.760293000
6	-0.212437000	2.940081000	-1.587776000
6	-1.173718000	3.632125000	-2.560144000
1	-0.611880000	4.247751000	-3.271753000
1	-1.749042000	2.888644000	-3.121919000
1	-1.873162000	4.274495000	-2.020569000
1	-0.080002000	4.586697000	-0.178401000
1	1.188414000	4.590620000	-1.416831000
6	0.705243000	1.964849000	-2.331320000
1	1.399836000	1.474333000	-1.643979000
1	0.116094000	1.199319000	-2.846858000
1	1.291761000	2.510394000	-3.078340000

TS

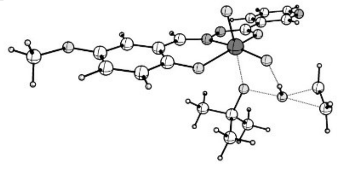

42	0.455243000	1.040208000	1.400969000
8	-1.285749000	0.222523000	0.770933000
8	2.273682000	1.560220000	0.965164000
8	0.836832000	-0.096869000	2.595945000
8	-0.251758000	2.383278000	2.237539000
7	-0.208514000	-1.111874000	-0.779002000
6	-2.600691000	-1.244880000	-0.573792000
6	4.620400000	1.334207000	1.029025000
1	4.656510000	2.242889000	1.621025000
6	4.499715000	-0.978340000	-0.513086000
1	4.468105000	-1.880701000	-1.116572000
7	0.889521000	-0.478335000	-0.241342000
6	3.369246000	0.850832000	0.635444000
8	6.829324000	-1.210725000	-0.513405000
6	3.304927000	-0.333465000	-0.149383000
7	-4.996727000	-1.268115000	-0.252037000
6	2.057212000	-0.893304000	-0.618819000
1	2.094021000	-1.723620000	-1.325288000
6	-5.082094000	-2.184727000	-1.224175000
1	-6.084801000	-2.537519000	-1.460761000
6	-3.975575000	-2.684820000	-1.917049000
1	-4.106886000	-3.430193000	-2.695693000
6	-1.292008000	-0.689453000	-0.180584000
6	8.115637000	-0.755513000	-0.126469000
1	8.326552000	0.246692000	-0.523391000
1	8.228630000	-0.742084000	0.965897000
1	8.825111000	-1.467198000	-0.552330000

6	5.795215000	0.674873000	0.671440000
1	6.744911000	1.080107000	1.001416000
6	-3.777510000	-0.816524000	0.058972000
1	-3.719268000	-0.077031000	0.852792000
6	-2.712653000	-2.207896000	-1.587048000
1	-1.819162000	-2.559380000	-2.091624000
6	5.741237000	-0.492604000	-0.106598000
1	-1.059157000	3.238518000	1.049310000
8	-1.500845000	3.463120000	0.182786000
8	-0.109048000	2.375078000	-0.508849000
1	1.983992000	4.054471000	-0.267693000
6	1.212185000	4.367214000	-0.978344000
6	0.489906000	3.138534000	-1.554453000
6	-0.599630000	3.557840000	-2.558074000
1	-0.154633000	4.067803000	-3.420726000
1	-1.139822000	2.675362000	-2.917339000
1	-1.317389000	4.235429000	-2.089006000
1	0.499425000	5.011083000	-0.453766000
1	1.687949000	4.954899000	-1.772811000
6	1.494093000	2.220864000	-2.283006000
1	2.316544000	1.929908000	-1.627324000
1	0.992457000	1.318941000	-2.647170000
1	1.917168000	2.752313000	-3.143841000
1	-2.338151000	4.997000000	2.242214000
6	-2.758431000	4.882457000	1.248085000
1	-2.765853000	5.753422000	0.602559000
6	-3.211295000	3.658517000	0.801309000
1	-3.320625000	2.822402000	1.484479000
1	-3.747702000	3.574369000	-0.137212000

MoO(OR)(OH)L²

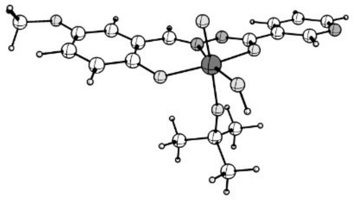

42	0.189384000	0.895975000	-1.132356000
8	2.015564000	0.073076000	-0.900397000
8	-1.656332000	1.093366000	-0.572913000
8	-0.254067000	-0.107160000	-2.430513000
8	0.739619000	2.353103000	-2.226504000
7	1.200615000	-1.538885000	0.526472000
6	3.561081000	-1.477920000	0.048753000
6	-3.969491000	0.613059000	-0.591192000
1	-4.143494000	1.579159000	-1.053415000
6	-3.499114000	-1.851397000	0.618695000
1	-3.330424000	-2.816592000	1.087018000
7	0.014942000	-0.918042000	0.194639000
6	-2.652514000	0.231181000	-0.321576000
8	-5.784311000	-2.353588000	0.694622000
6	-2.409052000	-1.027421000	0.289364000
7	5.901083000	-1.277039000	-0.517697000
6	-1.076988000	-1.497021000	0.588522000
1	-0.969718000	-2.421304000	1.156542000
6	6.145024000	-2.329757000	0.272339000
1	7.185415000	-2.643963000	0.340543000
6	5.153355000	-3.012686000	0.983246000
1	5.412202000	-3.862184000	1.608025000
6	2.188601000	-0.964950000	-0.109110000
6	-7.132591000	-2.018564000	0.408888000
1	-7.445781000	-1.109491000	0.939960000

1	-7.295406000	-1.881456000	-0.668518000
1	-7.730513000	-2.862056000	0.758384000
6	-5.038626000	-0.222548000	-0.271517000
1	-6.045752000	0.105109000	-0.502545000
6	4.632814000	-0.867546000	-0.621316000
1	4.447106000	-0.013888000	-1.267525000
6	3.837656000	-2.580601000	0.869959000
1	3.027433000	-3.072467000	1.397429000
6	-4.809003000	-1.465661000	0.340127000
1	1.107484000	3.070913000	-1.680544000
8	0.608652000	1.979179000	0.425075000
1	0.214119000	4.553207000	0.927413000
6	0.997892000	4.064905000	1.517393000
6	0.674250000	2.572629000	1.708042000
6	1.792222000	1.874179000	2.501030000
1	1.885723000	2.305801000	3.504376000
1	1.577320000	0.805222000	2.601702000
1	2.752054000	1.984738000	1.985425000
1	1.954350000	4.184616000	0.995845000
1	1.069067000	4.577310000	2.483613000
6	-0.683429000	2.401414000	2.410132000
1	-1.481343000	2.858985000	1.817186000
1	-0.918487000	1.339921000	2.539137000
1	-0.667497000	2.873652000	3.399391000

B3 - With Ligand L^3

[MoO$_2$L^3]$_2$

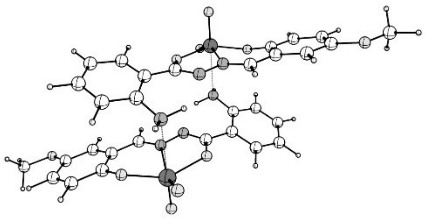

42	2.205517000	-2.731399000	-0.540364000
8	2.817790000	-3.208164000	1.240762000
8	1.156231000	-1.419242000	-1.671221000
7	2.323864000	-0.650453000	0.398865000
8	5.935074000	-0.881489000	5.174129000
8	3.679566000	-2.658741000	-1.378363000
7	1.724036000	0.359021000	-0.324667000
8	1.381050000	-4.144093000	-1.029451000
7	0.106432000	2.601149000	-0.827563000
1	0.234161000	3.609205000	-0.785899000
1	0.774089000	2.132123000	-0.212227000
6	1.173735000	-0.126540000	-1.411972000
6	0.584587000	0.770357000	-2.425859000
6	-0.341046000	2.409655000	-4.504859000
1	-0.691497000	3.057478000	-5.303652000
6	-0.284970000	2.899073000	-3.204145000
1	-0.611132000	3.912185000	-2.986899000
6	3.686716000	-1.217590000	2.319712000
6	0.495384000	0.287792000	-3.744946000
1	0.812337000	-0.731127000	-3.937586000
6	0.041150000	1.093682000	-4.781065000
1	-0.006610000	0.704774000	-5.793482000
6	2.976767000	-0.298689000	1.463105000
1	3.007126000	0.763725000	1.708114000
6	0.165815000	2.095705000	-2.146880000
6	4.476593000	-0.686004000	3.353840000
1	4.540907000	0.389271000	3.491671000
6	3.608303000	-2.626665000	2.160525000
6	5.124375000	-2.903983000	4.041795000
1	5.674605000	-3.573882000	4.692670000
6	4.335393000	-3.446086000	3.028332000
1	4.267621000	-4.521276000	2.897915000
6	5.198071000	-1.512258000	4.213312000
6	6.695832000	-1.677969000	6.067838000
1	7.446683000	-2.278097000	5.536575000
1	6.055672000	-2.344212000	6.661894000
1	7.201864000	-0.977887000	6.734852000
42	-2.205517000	2.731399000	0.540364000
8	-2.817790000	3.208164000	-1.240762000
8	-1.156231000	1.419242000	1.671221000
7	-2.323864000	0.650453000	-0.398865000
8	-5.935074000	0.881489000	-5.174129000
8	-3.679566000	2.658741000	1.378363000
7	-1.724036000	-0.359021000	0.324667000
8	-1.381050000	4.144093000	1.029451000
7	-0.106432000	-2.601149000	0.827563000
1	-0.234161000	-3.609205000	0.785899000
1	-0.774089000	-2.132123000	0.212227000
6	-1.173735000	0.126540000	1.411972000
6	-0.584587000	-0.770357000	2.425859000
6	0.341046000	-2.409655000	4.504859000
1	0.691497000	-3.057478000	5.303652000
6	0.284970000	-2.899073000	3.204145000
1	0.611132000	-3.912185000	2.986899000
6	-3.686716000	1.217590000	-2.319712000
6	-0.495384000	-0.287792000	3.744946000
1	-0.812337000	0.731127000	3.937586000
6	-0.041150000	-1.093682000	4.781065000
1	0.006610000	-0.704774000	5.793482000
6	-2.976767000	0.298689000	-1.463105000
1	-3.007126000	-0.763725000	-1.708114000
6	-0.165815000	-2.095705000	2.146880000
6	-4.476593000	0.686004000	-3.353840000
1	-4.540907000	-0.389271000	-3.491671000
6	-3.608303000	2.626665000	-2.160525000
6	-5.124375000	2.903983000	-4.041795000
1	-5.674605000	3.573882000	-4.692670000
6	-4.335393000	3.446086000	-3.028332000
1	-4.267621000	4.521276000	-2.897915000
6	-5.198071000	1.512258000	-4.213312000
6	-6.695832000	1.677969000	-6.067838000
1	-7.446683000	2.278097000	-5.536575000
1	-6.055672000	2.344212000	-6.661894000
1	-7.201864000	0.977887000	-6.734852000

MoO$_2$L^3(MeOH)

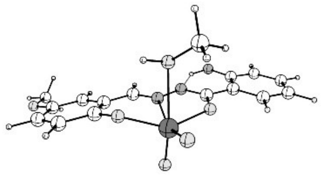

42	0.057873000	-1.618291000	-0.533534000
8	1.887717000	-0.844000000	-0.283766000
8	-1.771613000	-1.573380000	0.173124000
8	0.474209000	-3.259324000	-0.319938000
8	0.274583000	-1.592025000	1.960913000
1	-0.605100000	-1.936810000	2.188434000
7	2.940980000	3.240904000	0.489648000
8	-6.058498000	1.911472000	-0.004680000
7	1.078609000	1.275537000	0.111170000
7	-0.113800000	0.601375000	0.015236000
8	-0.213333000	-1.363912000	-2.188027000
6	-2.763951000	-0.666608000	0.082409000
6	2.089380000	0.453996000	-0.083638000
6	-3.632093000	1.618192000	0.075813000
1	-3.430059000	2.683603000	0.089460000
6	3.851219000	2.273659000	0.157449000
6	-2.530516000	0.728978000	0.094061000
6	-4.934570000	1.137343000	0.026807000
6	4.488477000	-0.032013000	-0.362460000
1	4.182820000	-1.055857000	-0.545085000
6	3.478238000	0.915011000	-0.084988000
6	5.223156000	2.608811000	0.087157000
1	5.512338000	3.641509000	0.269130000
6	-1.202807000	1.292319000	0.151289000
1	-1.104341000	2.368268000	0.305812000
6	-4.087039000	-1.136723000	0.037188000
1	-4.250437000	-2.209509000	0.029248000
6	-5.153353000	-0.253835000	0.007316000
1	-6.176411000	-0.614645000	-0.030483000

6	5.826418000	0.318519000	-0.419149000
1	6.581476000	-0.429270000	-0.639740000
6	1.282964000	-2.401941000	2.589797000
1	2.242359000	-1.993541000	2.271726000
1	1.209490000	-3.444088000	2.264205000
1	1.198636000	-2.329815000	3.679767000
6	-5.898842000	3.320075000	-0.001237000
1	-5.332759000	3.663679000	-0.877623000
1	-5.395775000	3.667113000	0.911656000
1	-6.906794000	3.737028000	-0.037173000
6	6.187110000	1.656013000	-0.194384000
1	7.231642000	1.954016000	-0.239503000
1	3.225160000	4.205496000	0.404762000
1	1.959310000	3.014916000	0.369868000

MoO_2L^3

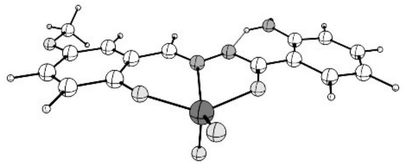

42	-0.115035000	-1.827918000	0.101699000
8	-1.924330000	-1.035092000	-0.176508000
8	1.732765000	-1.639083000	-0.475948000
8	-0.474294000	-3.267582000	-0.724204000
7	-3.024632000	3.108147000	-0.118635000
8	5.983885000	1.830543000	0.093248000
7	-1.141638000	1.116841000	-0.106359000
7	0.051364000	0.430582000	-0.091233000
8	-0.034828000	-2.109597000	1.772886000
6	2.721176000	-0.752928000	-0.293456000
6	-2.152179000	0.273987000	-0.117867000
6	3.560837000	1.525930000	0.010016000
1	3.345394000	2.580547000	0.141312000
6	-3.930535000	2.085294000	-0.052271000
6	2.471771000	0.627106000	-0.114800000
6	4.866946000	1.058291000	-0.026502000
6	-4.548005000	-0.285765000	-0.073117000
1	-4.231906000	-1.322078000	-0.105237000
6	-3.544556000	0.709090000	-0.087153000
6	-5.310547000	2.385952000	0.016512000
1	-5.611047000	3.430884000	0.044714000
6	1.136976000	1.153027000	-0.094416000
1	1.007326000	2.235763000	-0.076716000
6	4.049429000	-1.210591000	-0.336811000
1	4.225316000	-2.271208000	-0.481801000
6	5.101268000	-0.322354000	-0.202023000
1	6.129842000	-0.667923000	-0.231410000
6	-5.892502000	0.033934000	-0.012373000
1	-6.643357000	-0.749435000	0.000068000
6	5.814837000	3.227283000	0.274319000
1	5.261409000	3.449209000	1.196715000
1	5.294396000	3.683687000	-0.578500000
1	6.820509000	3.644284000	0.347907000
6	-6.267248000	1.386153000	0.036160000
1	-7.318432000	1.658202000	0.088267000
1	-3.332017000	4.033960000	0.138895000
1	-2.043242000	2.877714000	-0.015095000

$MoO_2L^3(TBHP)$

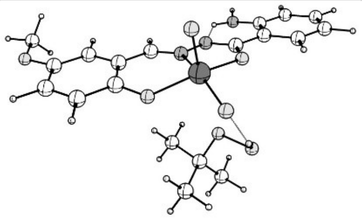

42	-0.074846000	-0.702266000	-1.570721000
8	-1.882163000	-0.127859000	-0.990991000
8	1.748064000	-0.979024000	-0.963726000
8	-0.512181000	-2.316630000	-1.952027000
7	-2.926458000	3.125829000	1.597143000
8	6.071045000	2.091713000	0.570473000
7	-1.071105000	1.522250000	0.377586000
7	0.112146000	0.969213000	-0.049351000
8	0.144258000	0.163385000	-3.008253000
6	2.756950000	-0.170713000	-0.597444000
6	-2.091233000	0.903294000	-0.179404000
6	3.642306000	1.830575000	0.492840000
1	3.448798000	2.762635000	1.012108000
6	-3.842622000	2.401966000	0.884804000
6	2.534882000	1.051321000	0.077360000
6	4.939793000	1.412545000	0.227061000
6	-4.491025000	0.566624000	-0.603375000
1	-4.189729000	-0.270798000	-1.221818000
6	-3.475681000	1.300050000	0.050707000
6	-5.215333000	2.722946000	0.994918000
1	-5.500903000	3.561860000	1.625532000
6	1.209332000	1.515062000	0.389269000
1	1.104983000	2.388317000	1.034229000
6	4.074608000	-0.586704000	-0.849929000
1	4.228543000	-1.530049000	-1.363231000
6	5.146196000	0.192363000	-0.448932000
1	6.166973000	-0.120165000	-0.645098000
6	-5.828346000	0.895012000	-0.472888000
1	-6.588789000	0.315719000	-0.986468000
6	5.928264000	3.333612000	1.240823000
1	5.373182000	4.058535000	0.630390000
1	5.422885000	3.213438000	2.208643000
1	6.941493000	3.702986000	1.407181000
6	-6.183611000	1.988677000	0.332707000
1	-7.228857000	2.265793000	0.444359000
1	-3.211507000	4.016815000	1.974859000
1	-1.944830000	2.974872000	1.395431000
8	-1.710506000	-3.103577000	0.409472000
8	-0.767673000	-2.153097000	0.983964000
6	0.021329000	-2.836333000	2.001866000
6	-0.919078000	-3.400408000	3.072716000
1	-1.579953000	-4.157622000	2.645136000
1	-1.536606000	-2.601364000	3.496283000
1	-0.338797000	-3.858490000	3.881826000
1	0.244954000	-4.694255000	0.901321000
6	0.879205000	-3.930441000	1.359401000
1	1.529559000	-3.505137000	0.587810000
1	1.507863000	-4.415528000	2.114839000
1	1.565397000	-1.307822000	1.819564000
6	0.884819000	-1.708347000	2.575973000
1	0.254893000	-0.895155000	2.951456000
1	1.485402000	-2.092323000	3.407523000
1	-1.364792000	-3.124047000	-0.511376000

TS

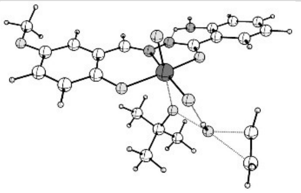

42	-0.064539000	-0.909551000	-1.057168000
8	-1.786167000	-0.047263000	-0.482234000
8	1.765330000	-1.380003000	-0.595267000
8	-0.775696000	-2.334582000	-1.742235000
7	-2.301748000	3.058415000	2.422216000
8	6.441924000	1.390878000	0.393360000
7	-0.708127000	1.427772000	0.917347000
7	0.384495000	0.758957000	0.422788000
8	0.304965000	0.097467000	-2.367427000
6	2.858727000	-0.634686000	-0.360998000
6	-1.815201000	0.956918000	0.378323000
6	4.003849000	1.314575000	0.568968000
1	3.937426000	2.270964000	1.076039000
6	-3.322245000	2.565926000	1.653245000
6	2.801707000	0.618796000	0.295695000
6	5.232649000	0.791428000	0.186085000
6	-4.245457000	1.039437000	-0.019853000
1	-4.071696000	0.257671000	-0.750363000
6	-3.127967000	1.529188000	0.688967000
6	-4.632802000	3.063187000	1.840181000
1	-4.785996000	3.851905000	2.573474000
6	1.559439000	1.211305000	0.733538000
1	1.607569000	2.105971000	1.356505000
6	4.110395000	-1.155635000	-0.730158000
1	4.138460000	-2.120803000	-1.225151000
6	5.275600000	-0.455469000	-0.467333000
1	6.243236000	-0.852904000	-0.757139000
6	-5.521094000	1.539045000	0.181700000
1	-6.361292000	1.148184000	-0.383674000
6	6.458548000	2.651913000	1.040352000
1	5.900898000	3.406045000	0.468478000
1	6.043502000	2.590313000	2.055702000
1	7.507848000	2.946699000	1.097922000
6	-5.706042000	2.563450000	1.122524000
1	-6.698050000	2.973735000	1.294475000
1	-2.450744000	3.936954000	2.896008000
1	-1.356473000	2.814929000	2.144869000
6	-3.272168000	-4.728872000	-0.482664000
1	-2.897637000	-4.914670000	-1.484262000
1	-3.255344000	-5.553407000	0.221081000
1	-4.182767000	-3.317287000	0.854677000
6	-3.684728000	-3.468944000	-0.096599000
1	-3.825681000	-2.681665000	-0.830207000
1	-1.551394000	-3.080944000	-0.463688000
8	-1.968932000	-3.231614000	0.430852000
8	-0.578863000	-2.073653000	0.992120000
6	0.048624000	-2.732264000	2.089728000
1	1.538087000	-3.734574000	0.865448000
6	1.048789000	-1.736761000	2.714461000
1	1.858940000	-1.500122000	2.022558000
1	0.537925000	-0.810869000	2.996742000
1	1.489858000	-2.178136000	3.616268000
1	1.278605000	-4.504939000	2.448099000
6	0.782082000	-3.996208000	1.612697000
6	-1.016724000	-3.076411000	3.146969000
1	-0.547511000	-3.491284000	4.047058000
1	-1.725434000	-3.811505000	2.757256000
1	-1.571622000	-2.174953000	3.428227000
1	0.072131000	-4.693853000	1.157639000

MoO(OR)(OH)L³

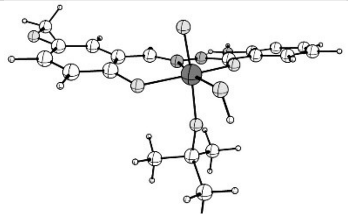

42	-0.047265000	-1.174109000	-1.064491000
8	-1.862657000	-0.353604000	-0.904957000
8	1.816962000	-1.278276000	-0.517754000
8	-0.590834000	-2.768514000	-1.956790000
7	-2.928795000	3.161288000	1.297101000
8	6.060316000	2.170850000	0.215643000
7	-1.063339000	1.422292000	0.303003000
7	0.125176000	0.789302000	0.019838000
8	0.380079000	-0.354842000	-2.491177000
6	2.801139000	-0.383392000	-0.378890000
6	-2.071128000	0.769332000	-0.238512000
6	3.640740000	1.817161000	0.275739000
1	3.426729000	2.825498000	0.612812000
6	-3.825430000	2.424006000	0.569129000
6	2.551796000	0.938195000	0.059560000
6	4.945598000	1.399891000	0.047695000
6	-4.448614000	0.510123000	-0.824129000
1	-4.140775000	-0.378956000	-1.362691000
6	-3.451481000	1.246436000	-0.148449000
6	-5.186067000	2.807298000	0.552551000
1	-5.477113000	3.703020000	1.096732000
6	1.218828000	1.424462000	0.314480000
1	1.110888000	2.411894000	0.764500000
6	4.127168000	-0.794587000	-0.596812000
1	4.304367000	-1.812710000	-0.927249000
6	5.179087000	0.081887000	-0.392296000
1	6.204599000	-0.228516000	-0.566465000
6	-5.776254000	0.902271000	-0.821969000
1	-6.522107000	0.320233000	-1.353822000
6	5.886159000	3.508338000	0.651090000
1	5.287749000	4.090471000	-0.062913000
1	5.409771000	3.552243000	1.640160000
1	6.887897000	3.936866000	0.714935000
6	-6.138180000	2.065902000	-0.126244000
1	-7.174465000	2.394323000	-0.115150000
1	-3.197628000	4.097226000	1.562653000
1	-1.943897000	2.972257000	1.145067000
8	-0.410656000	-2.069968000	0.622999000
6	-0.417755000	-2.520957000	1.963703000
6	-1.547030000	-1.785354000	2.705908000
1	-2.511797000	-1.987308000	2.228795000
1	-1.375832000	-0.704058000	2.686125000
1	-1.601573000	-2.109927000	3.751689000
1	-1.642611000	-4.254023000	1.471924000
6	-0.679538000	-4.037373000	1.948346000
1	0.110112000	-4.555804000	1.392908000
1	-0.704830000	-4.441899000	2.966763000
1	1.750025000	-2.709857000	2.049578000
6	0.946510000	-2.217006000	2.605375000
1	1.139561000	-1.139344000	2.602593000
1	0.971041000	-2.569489000	3.643304000
1	-0.935244000	-3.414682000	-1.314608000

B4- With Ligand L^4

$[MoO_2L^4]_2$

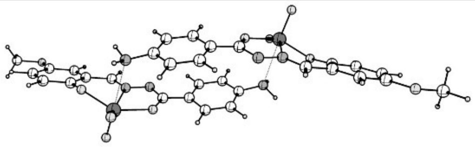

42	4.358866000	1.901492000	-0.482770000
7	4.292467000	-0.364827000	-0.818005000
7	3.091036000	-0.868481000	-1.261172000
8	2.521766000	1.359634000	-1.077971000
8	5.909285000	1.345180000	0.562423000
8	3.919565000	3.348911000	0.311757000
6	-1.921053000	-0.746599000	-1.962248000
6	2.217408000	0.102380000	-1.365389000
6	-0.983270000	-1.792889000	-1.947433000
1	-1.328798000	-2.822531000	-1.977980000
6	0.369228000	-1.513015000	-1.824629000
1	1.090645000	-2.320604000	-1.768779000
6	0.819906000	-0.185123000	-1.711709000
6	-0.115198000	0.857217000	-1.799706000
1	0.225965000	1.883588000	-1.726083000
6	-1.471074000	0.580545000	-1.933294000
1	-2.189397000	1.395864000	-1.965153000
6	5.279485000	-1.201790000	-0.715312000
1	5.100184000	-2.231057000	-1.029121000
6	6.590261000	-0.848276000	-0.221679000
6	6.863269000	0.411141000	0.375963000
6	8.153406000	0.672410000	0.844811000
1	8.348665000	1.637016000	1.302076000
6	9.167766000	-0.277995000	0.735557000
1	10.156090000	-0.035386000	1.108796000
6	8.904725000	-1.527986000	0.152796000
6	7.619443000	-1.801369000	-0.312454000
1	7.425534000	-2.770339000	-0.763046000
8	5.075791000	2.303196000	-1.965765000
7	-3.304197000	-1.037685000	-1.901274000
1	-3.553640000	-1.916572000	-2.347717000
1	-3.897427000	-0.290677000	-2.251134000
42	-4.358741000	-1.901470000	0.482429000
7	-4.292479000	0.364796000	0.818080000
7	-3.091015000	0.868518000	1.261083000
8	-2.521620000	-1.359532000	1.077635000
8	-5.909266000	-1.345059000	-0.562512000
8	-3.919350000	-3.348589000	-0.312606000
6	1.921165000	0.746971000	1.961629000
6	-2.217303000	-0.102306000	1.365093000
6	0.983295000	1.793178000	1.947004000
1	1.328724000	2.822850000	1.977679000
6	-0.369194000	1.513202000	1.824277000
1	-1.090686000	2.320736000	1.768605000
6	-0.819765000	0.185287000	1.711237000
6	0.115440000	-0.856981000	1.798993000
1	-0.225640000	-1.883374000	1.725272000
6	1.471305000	-0.580210000	1.932509000
1	2.189703000	-1.395468000	1.964212000
6	-5.279617000	1.201644000	0.715702000
1	-5.100391000	2.230900000	1.029598000
6	-6.590493000	0.848055000	0.222345000
6	-6.863457000	-0.411279000	-0.375549000
6	-8.153631000	-0.672673000	-0.844079000
1	-8.348920000	-1.637188000	-1.301520000
6	-9.168171000	0.277540000	-0.734291000
1	-10.156569000	0.034757000	-1.107197000
6	-8.905180000	1.527430000	-0.151365000
6	-7.619777000	1.800941000	0.313584000
1	-7.425959000	2.769858000	0.764328000
8	-5.075406000	-2.303720000	1.965402000
7	3.304301000	1.038193000	1.900692000
1	3.553600000	1.917076000	2.347229000
1	3.897570000	0.291233000	2.250603000
8	9.823054000	-2.526805000	0.000142000
6	11.152559000	-2.287938000	0.433550000
1	11.606105000	-1.442640000	-0.101035000
1	11.198513000	-2.098155000	1.514441000
1	11.709207000	-3.198463000	0.205152000
8	-9.823623000	2.526045000	0.002095000
6	-11.152657000	2.288277000	-0.433486000
1	-11.607847000	1.443447000	0.100405000
1	-11.708869000	3.199305000	-0.206047000
1	-11.196921000	2.098476000	-1.514436000

$MoO_2L^4(MeOH)$

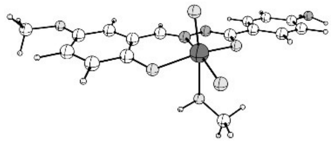

42	0.151482000	-1.516816000	-0.565604000
7	0.171958000	0.696921000	0.037475000
7	-1.061613000	1.291230000	0.140148000
7	-7.471105000	1.923329000	-0.055122000
1	-7.709414000	2.774401000	0.436562000
1	-8.151396000	1.186994000	0.078711000
8	-1.731427000	-0.873545000	-0.318065000
8	1.966377000	-1.371265000	0.160274000
8	-0.161952000	-3.185182000	-0.380942000
8	0.415247000	-1.215402000	-2.213443000
8	-0.085578000	-1.549077000	1.932953000
1	0.820459000	-1.826148000	2.149620000
6	-2.002095000	0.406525000	-0.082826000
6	-3.413079000	0.797674000	-0.063342000
6	-4.421697000	-0.150626000	-0.305366000
1	-4.141855000	-1.178181000	-0.511205000
6	-5.760249000	0.213156000	-0.285872000
1	-6.527976000	-0.531911000	-0.481452000
6	-6.137550000	1.544387000	-0.023302000
6	-5.125396000	2.496563000	0.219087000
1	-5.400526000	3.529815000	0.418206000
6	-3.790563000	2.128338000	0.197198000
1	-3.017169000	2.865306000	0.384363000
6	1.209720000	1.456933000	0.199313000
1	1.035521000	2.518838000	0.378294000
6	2.573527000	0.981596000	0.137669000
6	2.901409000	-0.399944000	0.092841000
6	4.246352000	-0.775606000	0.039456000
1	4.482375000	-1.834452000	0.006431000
6	5.264407000	0.176944000	0.034417000
1	6.294986000	-0.156285000	-0.009987000
6	4.949602000	1.543750000	0.088776000
6	3.611424000	1.929449000	0.145226000
1	3.378321000	2.989506000	0.183708000
6	-1.022105000	-2.455665000	2.537730000

1	-2.012066000	-2.120033000	2.227783000
1	-0.945233000	-2.406034000	3.629757000
1	-0.863270000	-3.479389000	2.185081000
8	5.867167000	2.556523000	0.092487000
6	7.240830000	2.215339000	0.012326000
1	7.559579000	1.610764000	0.872409000
1	7.469783000	1.671952000	-0.914343000
1	7.785066000	3.161495000	0.017799000

MoO_2L^4

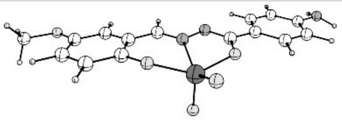

42	-0.102549000	1.737629000	0.090447000
7	-0.132709000	-0.530555000	-0.086424000
7	1.097127000	-1.147936000	-0.093519000
8	1.757703000	1.052806000	-0.163722000
8	-1.937840000	1.438669000	-0.469903000
8	0.176725000	3.174772000	-0.771526000
6	6.183099000	-1.345752000	-0.009227000
6	2.044540000	-0.244046000	-0.101989000
6	5.175009000	-2.332460000	0.034816000
1	5.456486000	-3.381417000	0.090837000
6	3.837911000	-1.975766000	0.004359000
1	3.068611000	-2.739499000	0.042042000
6	3.454539000	-0.623121000	-0.072080000
6	4.458758000	0.359964000	-0.117428000
1	4.173243000	1.404675000	-0.175305000
6	5.799159000	0.007798000	-0.085937000
1	6.563983000	0.779746000	-0.124523000
6	-1.171043000	-1.318176000	-0.083676000
1	-0.972862000	-2.389878000	-0.058208000
6	-2.537223000	-0.871232000	-0.101908000
6	-2.873804000	0.495458000	-0.277641000
6	-4.221440000	0.866696000	-0.305899000
1	-4.465082000	1.914310000	-0.448373000
6	-5.228610000	-0.084733000	-0.162722000
1	-6.262526000	0.239932000	-0.187823000
6	-4.905179000	-1.442814000	0.006458000
6	-3.565724000	-1.822591000	0.029688000
1	-3.323653000	-2.873109000	0.160288000
8	-0.191859000	2.052362000	1.755838000
7	7.520224000	-1.704464000	-0.027781000
1	7.749928000	-2.620368000	0.334055000
1	8.184778000	-0.991816000	0.242703000
8	-5.817391000	-2.447752000	0.152667000
6	-7.196047000	-2.113265000	0.135193000
1	-7.457124000	-1.429545000	0.953921000
1	-7.490812000	-1.661259000	-0.821343000
1	-7.732792000	-3.054148000	0.268155000

MoO_2L^4(TBHP)

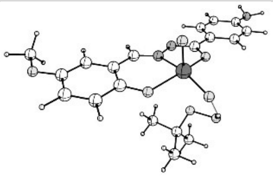

42	-0.127246000	0.619230000	-1.599940000
8	1.718261000	0.149769000	-1.035604000
8	-1.947710000	0.845367000	-0.966322000
8	0.236839000	2.238804000	-2.036556000
8	-6.106995000	-2.337777000	0.779057000
7	0.997096000	-1.475498000	0.423286000
7	-0.213664000	-0.991259000	-0.009036000
8	-0.332261000	-0.302785000	-3.004690000
6	-2.914996000	0.011057000	-0.547435000
6	1.968469000	-0.836342000	-0.179976000
6	-3.693268000	-1.979087000	0.639074000
1	-3.449908000	-2.880159000	1.190951000
6	3.712043000	-2.243987000	0.915660000
6	-2.628004000	-1.172151000	0.170422000
6	-5.012260000	-1.626319000	0.384127000
6	4.397969000	-0.429119000	-0.530569000
1	4.141318000	0.400450000	-1.180082000
6	3.366838000	-1.172526000	0.070153000
6	5.038555000	-2.561934000	1.151067000
1	5.290643000	-3.390387000	1.808998000
6	-1.277857000	-1.565750000	0.473839000
1	-1.117657000	-2.405105000	1.151063000
6	-4.253390000	0.361192000	-0.789417000
1	-4.456845000	1.275685000	-1.336663000
6	-5.283542000	-0.444736000	-0.335585000
1	-6.320210000	-0.183053000	-0.522671000
6	5.727839000	-0.744108000	-0.298416000
1	6.514160000	-0.156276000	-0.765882000
6	-5.897461000	-3.541331000	1.499683000
1	-5.325505000	-4.269625000	0.909136000
1	-5.376611000	-3.357432000	2.449104000
1	-6.890394000	-3.944426000	1.705864000
6	6.073613000	-1.816929000	0.546887000
8	1.446404000	3.170876000	0.264605000
8	0.571045000	2.197585000	0.904386000
6	-0.229987000	2.881297000	1.911351000
6	0.702785000	3.538262000	2.934991000
1	1.312965000	4.309518000	2.459527000
1	1.371014000	2.791081000	3.376086000
1	0.117834000	4.000105000	3.738589000
1	-0.578024000	4.673611000	0.736348000
6	-1.159636000	3.901271000	1.246749000
1	-1.808727000	3.408986000	0.514937000
1	-1.791166000	4.388471000	1.998483000
1	-1.698712000	1.272380000	1.828270000
6	-1.019941000	1.734463000	2.550589000
1	-0.339799000	0.968065000	2.936712000
1	-1.617331000	2.119112000	3.384240000
1	1.079212000	3.128774000	-0.647427000
1	2.921281000	-2.822358000	1.381112000
7	7.400709000	-2.103185000	0.821099000
1	7.603136000	-3.034524000	1.159093000
1	8.083408000	-1.764991000	0.156091000

TS

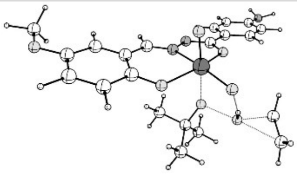

42	-0.071661000	-0.941016000	-1.085163000
8	-1.812020000	-0.100033000	-0.530456000
8	1.757641000	-1.386883000	-0.601503000
8	-0.760086000	-2.386935000	-1.753133000
8	6.398054000	1.428687000	0.432087000
7	-0.755601000	1.412718000	0.859080000

7	0.345464000	0.743778000	0.383067000
8	0.289713000	0.053127000	-2.407224000
6	2.842389000	-0.631039000	-0.357570000
6	-1.841736000	0.917958000	0.315771000
6	3.958297000	1.332559000	0.575595000
1	3.876728000	2.291054000	1.076397000
6	-3.277618000	2.554786000	1.544645000
6	2.765745000	0.625495000	0.290736000
6	5.196430000	0.817447000	0.211772000
6	-4.316248000	0.997691000	0.017919000
1	-4.229644000	0.184950000	-0.694964000
6	-3.153152000	1.492675000	0.630318000
6	-4.517479000	3.099034000	1.837519000
1	-4.596833000	3.916390000	2.550764000
6	1.512122000	1.209897000	0.706850000
1	1.537544000	2.109824000	1.322959000
6	4.102567000	-1.143392000	-0.708493000
1	4.145044000	-2.110989000	-1.197761000
6	5.258716000	-0.432109000	-0.434412000
1	6.233338000	-0.822958000	-0.709434000
6	-5.560465000	1.541266000	0.304144000
1	-6.450704000	1.146211000	-0.180103000
6	6.394462000	2.692553000	1.073417000
1	5.837840000	3.439217000	0.490808000
1	5.965999000	2.631964000	2.083257000
1	7.440218000	2.997191000	1.144012000
6	-5.684306000	2.602410000	1.221080000
6	-3.180146000	-4.849219000	-0.529628000
1	-2.787120000	-5.018345000	-1.527010000
1	-3.147940000	-5.676301000	0.170547000
1	-4.145150000	-3.470120000	0.803406000
6	-3.627120000	-3.600958000	-0.140411000
1	-3.784866000	-2.816301000	-0.873608000
1	-1.512209000	-3.144510000	-0.482468000
8	-1.937658000	-3.312213000	0.406383000
8	-0.596767000	-2.101261000	0.981625000
6	0.028877000	-2.728520000	2.097138000
1	1.562404000	-3.711049000	0.911825000
6	0.994354000	-1.699607000	2.722588000
1	1.810277000	-1.454763000	2.040365000
1	0.457422000	-0.781440000	2.980358000
1	1.430278000	-2.116129000	3.638678000
1	1.297507000	-4.463778000	2.502079000
6	0.801488000	-3.980688000	1.651214000
6	-1.043973000	-3.084427000	3.143120000
1	-0.579375000	-3.477113000	4.055553000
1	-1.729595000	-3.840629000	2.752112000
1	-1.623814000	-2.192638000	3.404341000
1	0.116706000	-4.702805000	1.195738000
1	-2.383740000	2.944995000	2.018905000
7	-6.932345000	3.114321000	1.551376000
1	-6.940789000	4.049766000	1.936724000
1	-7.666351000	2.968942000	0.870401000

MoO(OR)(OH)L^4

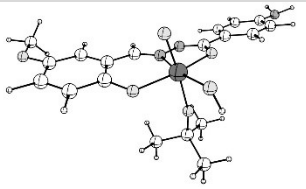

42	-0.151828000	1.107799000	-1.094943000
8	1.709969000	0.404144000	-0.897008000
8	-2.019895000	1.133112000	-0.546413000
8	0.327224000	2.682017000	-2.062338000
8	-6.062222000	-2.515150000	0.344586000
7	1.004547000	-1.365417000	0.391095000
7	-0.214155000	-0.808381000	0.079782000
8	-0.540806000	0.211106000	-2.485179000
6	-2.952323000	0.190376000	-0.369963000
6	1.963635000	-0.679411000	-0.180290000
6	-3.666897000	-2.019996000	0.389763000
1	-3.396505000	-2.996686000	0.775918000
6	3.712711000	-2.234034000	0.692457000
6	-2.629160000	-1.091912000	0.132361000
6	-4.992663000	-1.689912000	0.139549000
6	4.384610000	-0.354142000	-0.671304000
1	4.124055000	0.524589000	-1.251412000
6	3.362283000	-1.091676000	-0.050659000
6	5.036238000	-2.623915000	0.812348000
1	5.291982000	-3.506759000	1.393838000
6	-1.271062000	-1.488087000	0.410142000
1	-1.102289000	-2.442823000	0.908738000
6	-4.298526000	0.513196000	-0.611067000
1	-4.532432000	1.502109000	-0.991345000
6	-5.299744000	-0.411263000	-0.365549000
1	-6.340575000	-0.169247000	-0.556627000
6	5.711582000	-0.741852000	-0.556458000
1	6.491102000	-0.159376000	-1.042007000
6	-5.812052000	-3.816188000	0.847208000
1	-5.178335000	-4.398181000	0.164174000
1	-5.337095000	-3.782317000	1.837394000
1	-6.787373000	-4.298744000	0.932453000
6	6.062432000	-1.884508000	0.188119000
8	0.146009000	2.114544000	0.544294000
6	0.083347000	2.651450000	1.850710000
6	1.233248000	2.037437000	2.668232000
1	2.197321000	2.268811000	2.203040000
1	1.128037000	0.948482000	2.713245000
1	1.237936000	2.430039000	3.692003000
1	1.214014000	4.423059000	1.276951000
6	0.252058000	4.177328000	1.741528000
1	-0.550530000	4.607136000	1.131576000
1	0.221013000	4.648872000	2.730620000
1	-2.093578000	2.711167000	1.874375000
6	-1.277075000	2.304548000	2.478605000
1	-1.405024000	1.219021000	2.539664000
1	-1.350765000	2.718635000	3.491246000
1	0.639593000	3.368805000	-1.446705000
1	2.927977000	-2.810184000	1.170637000
7	7.392133000	-2.246749000	0.348465000
1	7.565724000	-3.211876000	0.596832000
1	8.036778000	-1.901085000	-0.3503070
